# Associations of modifiable preconception, pregnancy and postpartum factors with health outcomes for women with type 2 diabetes and their children: A systematic review and meta‐analysis of observational studies

**DOI:** 10.1111/dme.70183

**Published:** 2025-12-07

**Authors:** Danielle Schoenaker, Eleanor Dyer, Nicola Heslehurst, Grainne Kent, Sowmiya Gunabalasingam, Lily Hopkins, Artemis Kyrka, Rivka Lebrett, Angela C. Flynn, Sara L. White, Claire L. Meek, Rita Forde

**Affiliations:** ^1^ School of Human Development and Health, Faculty of Medicine University of Southampton Southampton UK; ^2^ MRC Lifecourse Epidemiology Centre University of Southampton Southampton UK; ^3^ NIHR Southampton Biomedical Research Centre University of Southampton, and University Hospital Southampton NHS Foundation Trust Southampton UK; ^4^ Population Health Sciences Institute Newcastle University Newcastle upon Tyne UK; ^5^ Fuse, the Centre for Translational Research in Public Health Newcastle University Newcastle upon Tyne UK; ^6^ School of Population Health Royal College of Surgeons in Ireland Dublin Ireland; ^7^ Department of Women and Children's Health, School of Life Course and Population Sciences King's College London London UK; ^8^ Department of non‐Communicable Disease Epidemiology, Faculty of Epidemiology and Population Health London School of Hygiene and Tropical Medicine London UK; ^9^ Clalit Health Services Rehovot Israel; ^10^ Salford Royal Hospital Northwest of England Foundation School Salford UK; ^11^ Department of Diabetes and Endocrinology Guy's and St Thomas' Hospital NHS Foundation Trust London UK; ^12^ Leicester Diabetes Centre and Leicester NIHR Biomedical Research Centre University of Leicester, Leicester General Hospital Leicester UK; ^13^ Faculty of Nursing, Midwifery and Palliative Care King's College London London UK; ^14^ School of Nursing and Midwifery University College Cork Cork Ireland

**Keywords:** perinatal, postpartum, preconception, pregnancy, reproductive health, type 2 diabetes, women's health

## Abstract

**Aim:**

Type 2 diabetes (T2D) in pregnancy is increasingly common and associated with suboptimal outcomes for these women and their children. We aimed to synthesize observational evidence on associations of modifiable preconception, pregnancy and postpartum risk factors with perinatal outcomes among women with pregestational T2D.

**Methods:**

Searches were conducted in six databases (September 2023). Observational studies among women with pregestational T2D were included if they reported associations of modifiable risk factors with maternal and/or child outcomes. Screening, data extraction and quality assessments were conducted by two reviewers. Findings were synthesized through random effects meta‐analysis or narrative synthesis when results were too few or heterogeneous to pool.

**Results:**

Searches identified 15,578 results; 58 studies were included. Meta‐analysis showed excessive gestational weight gain (GWG) was associated with large for gestational age (LGA) (OR 2.39, 95%CI 1.74–3.29) but not small for gestational age (SGA). Meta‐analysis demonstrated no associations between preconception care or metformin use with adverse pregnancy, birth and neonatal outcomes. However, narrative synthesis showed preconception care was associated with increased use of folic acid and vitamin D, and reduced GWG. Further narrative synthesis findings showed that higher BMI was associated with multiple suboptimal pregnancy, birth and neonatal outcomes. Excessive GWG was associated with increased insulin requirements and increased likelihood of neonatal hypoglycaemia. The use of metformin/oral hypoglycaemic medications was associated with reduced GWG and fewer caesarean deliveries. There was mixed or no evidence of association for other reported exposures and outcomes.

**Conclusion:**

Based on observational evidence, increasing access to preconception care could be beneficial to optimize maternal nutrition and weight‐related outcomes, and addressing obesity and GWG has the potential to improve maternal and neonatal outcomes in pregnancies affected by T2D.

**What's new?**

**What is already known?**

- T2D prevalence in pregnancy is increasing internationally.
- Women with T2D have suboptimal pregnancy outcomes, not fully attributable to hyperglycaemia alone.

**What this study has found?**

- This systematic review of 58 observational studies identified access to preconception care, maternal glycaemia, BMI and gestational weight gain as key modifiable risk factors for suboptimal outcomes.
- Minimal evidence was available in preconception and postnatal periods.

**Implications of the study**

- Future research is needed in the preconception and postnatal periods, prioritizing the development of interventions to address access to preconception care, maternal BMI, glycaemia and weight gain to improve outcomes.

## 1 INTRODUCTION

Type 2 diabetes (T2D) in women of reproductive age is increasingly common globally.^1^ In England and Wales, for example, pregnancies in women with preexisting T2D now outnumber pregnancies in women with type 1 diabetes (T1D) annually.^2^ Women with T2D have an increased risk of perinatal complications, including miscarriage, stillbirth, preeclampsia and large for gestational age (LGA) infants.^3^, ^4^ Their children are at increased risk of neonatal hypoglycaemia and cardiometabolic disorders, including insulin resistance and obesity in later life.^5^ Recent UK National Pregnancy in Diabetes (NPID) audit data identified glycaemia as a key modifiable factor in determining perinatal outcomes of women with T2D.^4^ However, some adverse pregnancy outcomes are more common in women with T2D compared to T1D, despite lower HbA1c levels at conception.^4^, ^6^ Other modifiable (i.e. through behavioural or clinical intervention) and non‐modifiable factors must therefore also play a role in determining outcomes in T2D pregnancy.

Most women with T2D in pregnancy have early‐onset T2D (EOT2D), diagnosed before the age of 40 years, which is under‐represented in research.^7^ EOT2D is more medically complex than T2D that develops later in life and is associated with higher rates of microvascular and macrovascular complications, already evident at the time of diagnosis.^8^, ^9^ People with EOT2D are also at increased cardiovascular risk, resulting in premature mortality, particularly notable among women.^10^, ^11^ There is an urgent need to identify modifiable risk factors before, during and after pregnancy to inform new intervention opportunities and help improve short‐ and longer‐term outcomes for women with T2D and their children.

Current treatment approaches for pregnant women with T2D focus on reducing maternal hyperglycaemia using behaviour change measures, hypoglycaemic agents such as metformin and/or insulin.^12^ However, there is minimal evidence about the efficacy of these approaches or optimal delivery of care to women prior to, during and post pregnancy. To inform interventions, comprehensive evidence synthesis on potential modifiable risk factors is needed, including treatments (e.g. type of pharmacotherapy), healthcare (e.g. preconception care [PCC] attendance), health conditions (e.g. obesity and hypertension) and health behaviours (e.g. physical activity, diet, smoking). This systematic review therefore aimed to synthesize available evidence from observational studies among women with T2D to identify modifiable risk factors during the preconception, pregnancy and postpartum periods associated with maternal and child health outcomes.

## 2 METHODS

This systematic review is part of a series of reviews assessing the evidence on optimal management of women with T2D in the preconception, pregnancy and postpartum periods based on intervention, observational and qualitative studies. This systematic review includes evidence from observational studies and the other reviews are published separately.^13^, ^14^ The protocol for the series was prospectively registered with PROSPERO (CRD42021292405) and the Preferred Reporting Items for Systematic reviews and Meta‐Analyses (PRISMA 2020) guideline was used to ensure transparent reporting.^15^

### 2.1 Search strategy and selection criteria

A search strategy was developed, and final searches conducted in September 2023 in six databases: MEDLINE (Ovid), EMBASE (Ovid), CINAHL, PsycINFO, ASSIA and the Cochrane Library (File S1). The searches were restricted to studies among human participants and conducted using Medical Subject Headings (MeSH) terms. Backwards and forwards citation chaining was undertaken for all included studies using R Shiny, a citation chaser tool.^16^

Articles were selected based on inclusion and exclusion criteria developed using the PECOS framework (Table 1). Studies were included if they: (1) were conducted among women of reproductive age (18–50 years) with preexisting T2D (i.e. diagnosed prior to pregnancy); (2) reported findings on an association of a modifiable risk factor or exposure in the preconception, pregnancy and/or postpartum period (e.g. treatments, healthcare, health conditions and health behaviours) with an outcome during preconception, pregnancy, birth or beyond (e.g. preconception folic acid supplement use, stillbirth, preeclampsia, preterm birth, neonatal hypoglycaemia and child growth); (3) had an observational study design and (4) reported data collected from 2000 onwards based on substantial changes in the prevalence and management options for T2D. Articles were not included if they: (1) were conference abstracts or reviews; (2) were not published in the English language; (3) included women with other types of diabetes and did not report findings separately for women with T2D.

**TABLE 1:** PECOS framework.

Population	Women of reproductive age living with type 2 diabetes (diagnosed prior to pregnancy) (human participants)
Exposure	Modifiable treatments (pharmacotherapy, e.g. oral agents, insulin types; supplementation, e.g. folic acid supplement use; technology, e.g. sensors, apps, remote monitoring, accelerometer and insulin pumps), health conditions (e.g. HbA1c, blood pressure and obesity) and health behaviours (e.g. diet, physical activity, smoking, pregnancy planning and treatment adherence) during the preconception, pregnancy and/or postpartum period
Comparison	Not exposed or comparison group as defined by each study, or not applicable for studies that examine a continuous exposure
Outcome	Outcomes related to health conditions and health behaviours (see interventions/exposures) and clinical outcomes related to the mother (e.g. miscarriage, stillbirth, preeclampsia, mode of delivery and maternal mortality) and the offspring (e.g. birthweight, preterm birth, congenital anomaly, neonatal hypoglycaemia and perinatal mortality) during the preconception, pregnancy, birth or beyond
Study design	Observational studies (published in English)

### 2.2 Study selection

Search results were collated in EndNote and duplicates removed before uploading to the Covidence systematic review software.^17^ Titles and abstracts, followed by full‐text articles, were screened independently by two reviewers for inclusion. Disagreements or uncertainties were resolved through discussion.

### 2.3 Data extraction

A standardized data extraction form was created and piloted. Data were extracted on study design, participant characteristics and results on associations between exposures and outcomes. Where papers reported T2D and other groups (e.g. T1D, gestational diabetes (GDM) or no diabetes), only data for the T2D group were extracted. Data extraction was conducted by one reviewer and validated by a second reviewer. Disagreements were resolved between the two reviewers or through discussion with the wider authorship team.

### 2.4 Data analysis and synthesis

All extracted data on associations were grouped by exposure and outcome and assessed for suitability to pool in a meta‐analysis. Meta‐analysis was performed when at least three studies reported data suitable for pooling (i.e. comparable exposure and outcome) or if comparable effect estimates could be calculated from the data reported in each paper. Summary odds ratios (OR) and mean differences (MD) were calculated using random effects models. The *I* ^*2*^ statistic was used to describe variability in effect estimates due to heterogeneity, with a threshold of >75% indicating significant heterogeneity.^18^ No test for publication bias was performed, and no sensitivity or subgroup analyses were conducted, as each meta‐analysis contained fewer than 10 studies.^19^ Statistical analyses were conducted using Stata SE 17.0.

When meta‐analysis was not possible, a narrative synthesis was performed following recommendations by Popay et al.^20^ Across four overall categories of exposure (treatments, healthcare, health conditions, health behaviours), data from each study were transformed to facilitate comparisons across studies and outcomes if required and possible (i.e. using frequency data reported to calculate ORs and 95% CIs). Tables were used to group results into thematic categories to aid synthesis; these were based on the exposure being reported, with further thematic sub‐grouping by outcome. Tables were supplemented by a narrative description of patterns in the results.

### 2.5 Quality assessment

The quality of included studies was assessed using the Newcastle‐Ottawa Scale (NOS) for cohort studies (cross‐sectional and longitudinal studies). The scale was used to assess bias related to (1) selection (representativeness of the exposed cohort, selection of the non‐exposed cohort, ascertainment of exposure), (2) comparability (comparability of exposed and non‐exposed groups on the basis of the design or analysis, e.g. confounder adjustments) and (3) outcome (assessment of outcome, length of follow‐up, adequacy of follow‐up).^21^ The NOS item ‘demonstration that outcome of interest was not present at start of study’ was not included as this was not relevant for the outcomes of interest. Study quality was assessed by one reviewer and validated by a second reviewer. Disagreements were resolved between the two reviewers. Studies were classified as low (0–2 stars), medium (3–5 stars) or high (6–8 stars) quality.

## 3 RESULTS

**FIGURE 1:**
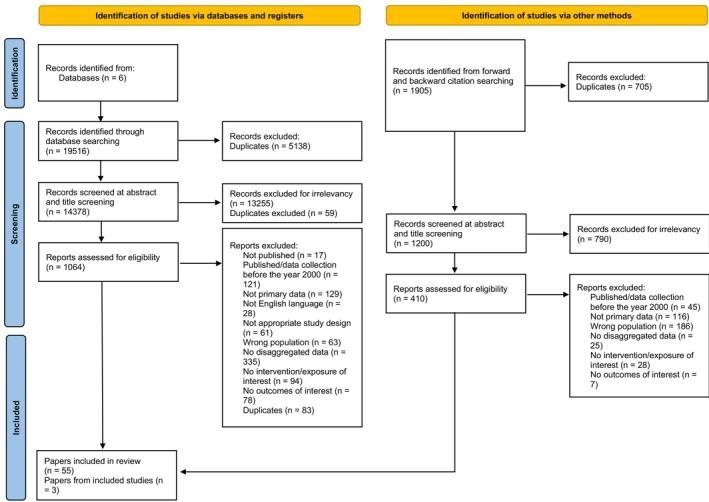
PRISMA flow diagram.

From 19,516 records identified from database searching and 1905 from citation searching, 5843 duplicates were removed. After title and abstract screening (*n* = 15,578), 1474 full‐text articles were evaluated for eligibility (Figure 1). Fifty‐eight observational articles met the inclusion criteria for this review.

### 3.1 Study characteristics

All included articles reported findings from cohort studies, with sample sizes ranging from 11 to 51,826 women with T2D (Table 2; most studies (78%) had *n* < 500). Studies were conducted in Europe (*n* = 22), North America (*n* = 16), South America (*n* = 3), Asia (*n* = 4), Australia or New Zealand (*n* = 7), Africa (*n* = 1) or across multiple continents (*n* = 5). Participants had a mean age between 29 and 38 years (Table S1). A wide range of modifiable exposures were examined related to treatments, healthcare, health conditions and behaviours during the preconception (*n* = 15), pregnancy (*n* = 49) and postpartum period (*n* = 1) (Table S2). Twenty‐two studies included only women with T2D, while the other studies included additional population groups (e.g., T1D, GDM, no diabetes).

**TABLE 2:** Summary of characteristics of included cohort studies (*N* = 58).

First author, year	Country	Total sample size^a^	Relevant exposures reported^b^	Timeframe(s) of outcomes reported^c^
Abell, 2017^81^	Australia	124	Health conditions	Pregnancy, birth, delivery, neonatal
Ahmed, 2022^68^	Pakistan	200	Health conditions	Neonatal
Allen, 2018^47^	The United States	33,777	Health care	Pregnancy, birth, delivery
Alexander, 2019^52^	Canada	253	Health conditions	Birth
Alrais, 2022^82^	The United States	215	Health care	Birth, neonatal
Arendt, 2021^67^	Denmark	397	Health conditions	Neonatal
Asbjornsdottir, 2013^34^	Denmark	58	Treatment and medication, health conditions	Pregnancy, birth, neonatal
Ásbjörnsdóttir, 2019^83^	Denmark	189	Health care	Pregnancy, birth, delivery, neonatal
Asbjornsdottir, 2021^84^	Denmark	90	Health conditions	Pregnancy, birth, delivery, neonatal
Bartal, 2019^29^	The United States	233	Treatment and medication	Pregnancy, birth, delivery, neonatal
Cesta, 2023^36^	Finland, Iceland, Norway, Sweden, The United States, Israel	51,826	Treatment and medication	Neonatal
Cordero, 2022^72^	The United States	294	Health behaviours	Postpartum
Colatrella, 2009^54^	Italy	76	Health conditions, health behaviours	Pregnancy, birth
Cyganek, 2011^43^	Poland	63	Health care	Pregnancy
Do, 2021^40^	Denmark	153	Treatment and medication	Pregnancy, birth
Egan, 2016^44^	Ireland	146	Health care	Pregnancy, birth, delivery, neonatal
Endo, 2018^62^	Japan	11	Health conditions	Pregnancy
Feghali, 2017^35^	The United States	198	Treatment and medication	Pregnancy, birth, delivery, neonatal
Feig, 2022^26^	Canada, Australia	460	Treatment and medication, health conditions, health behaviours	Birth
Gaudio, 2020^85^	The United Kingdom	4325	Health conditions	Preconception
Isabey, 2021^60^	Canada	192	Health conditions	Delivery
Kallas‐Koeman, 2012^42^	Canada	267	Health care	Pregnancy
Kapustin, 2022^33^	Russia	229	Treatment and medication	Pregnancy, birth
Kapustin, 2023^56^	Russia	214	Health conditions	Pregnancy, birth, neonatal
Kekki, 2021^70^	Finland	548	Health behaviours	Birth
Kjerpeseth, 2023^27^	Finland, Iceland, Norway, Sweden	4023	Treatment and medication	Neonatal
Ladfors, 2017^53^	Sweden	87	Health conditions, health behaviours	Birth
Lin, 2020^25^	Taiwan	848	Treatment and medication	Pregnancy, birth, delivery, neonatal
Longmore, 2022^71^	Australia	57	Health behaviours	Infancy
Maple‐Brown, 2019^23^	Australia	272	Treatment and medication	Birth, delivery
McLean, 2023^66^	Australia	41	Health conditions	Birth, neonatal
Morikawa, 2020^32^	Japan	109	Treatment and medication, health conditions	Pregnancy
Murphy, 2010^46^	The United Kingdom	271	Health care	Pregnancy, neonatal
Murphy, 2017^57^	The United Kingdom	1386	Health conditions	Pregnancy, birth
Murphy, 2021^4^	The United Kingdom	2320	Health care, health conditions, health behaviours	Pregnancy, birth, neonatal
Nadeau, 2021^30^	The United States	160	Treatment and medication, health conditions	Pregnancy, birth, delivery, neonatal
Nørgaard, 2016^55^	Denmark	105	Health conditions	Pregnancy, birth, delivery, neonatal
Olmos, 2009^61^	Chile	51	Health conditions	Birth
Oppermann, 2020^65^	Brazil	128	Health conditions, health behaviours	Pregnancy
Owens, 2015^64^	Ireland	108	Health conditions	Pregnancy, delivery, birth, neonatal
Parellada, 2014^50^	Denmark	142	Health conditions	Pregnancy, delivery, birth, neonatal
Perichart‐Perera, 2009^49^	Mexico	96	Health care	Pregnancy, birth, neonatal
Persson, 2016^86^	Sweden	886	Health conditions	Pregnancy
Racine, 2021^22^	The United States	254	Treatment and medication	Pregnancy, birth, neonatal
Rais, 2019^63^	The United States	136	Health conditions	Birth
Rasmussen, 2010^59^	Denmark	80	Treatment and medication, health conditions, health behaviours	Pregnancy
Roland, 2005^37^	The United Kingdom	146	Treatment and medication, health conditions, health behaviours	Neonatal
Rowan, 2009^24^	New Zealand	212	Health conditions, health behaviours	Birth
Rowe, 2021^31^	Australia	40	Treatment and medication	Pregnancy, neonatal
Shannon, 2016^38^	The United States	4166	Treatment and medication, health care	Birth
Soepnel, 2019^28^	South Africa	408	Treatment and medication	Pregnancy
Søholm, 2021^39^	Denmark	86	Treatment and medication, health conditions, health behaviours	Birth
Stafl, 2023^48^	Canada	902	Health care, health conditions, health behaviours	Pregnancy, birth, delivery, neonatal
Sushko, 2023^87^	Canada	56	Treatment and medication, health behaviours	Pregnancy
Wong, 2013^45^	Australia	117	Health care	Preconception, pregnancy, birth, neonatal
Yamamoto, 2018^41^	The United Kingdom	318	Health care	Preconception, pregnancy, birth, neonatal
Yamamoto, 2020^69^	Canada	267	Health conditions	Neonatal
Yee, 2011^51^	The United States	2310	Health conditions	Birth, delivery, neonatal

### 3.2 Quality assessment

The quality assessment guide is shown in Table S3. Quality scores of the included studies were rated between five and eight (Table S4). The majority were rated as high quality (89.7%) with four studies scoring the maximum of 8 stars. The remaining studies were rated as medium quality (10.3%); no studies were rated as poor quality. There was consistency across all included studies in their duration of follow‐up being long enough for outcomes to occur (100%). Almost all studies also rated high quality for their methods of assessing the outcomes (98.3%), selection and representativeness of the cohorts and ascertainment of the exposure (96.6%), and adequacy of follow‐up (86.2%). The lowest scoring component related to the lack of controlling for confounding factors in their design or analysis (50%).

### 3.3 Associations between modifiable risks and maternal and child health outcomes

#### 3.3.1 Treatment and medication

**FIGURE 2:**
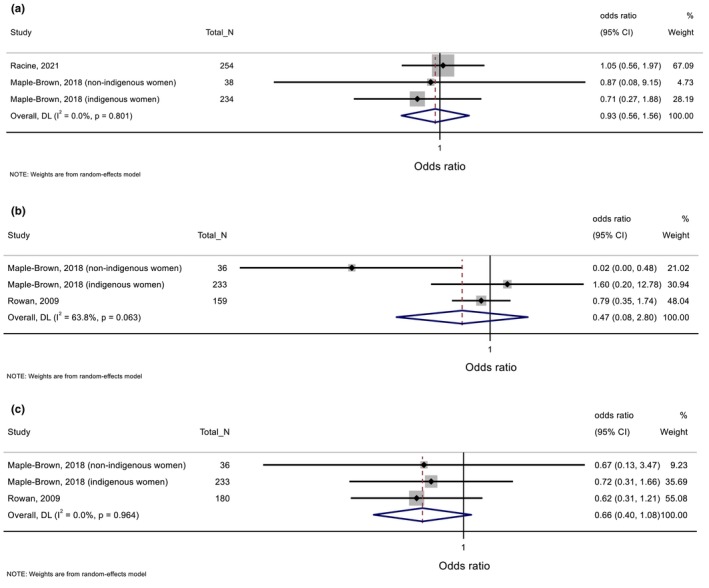
Metformin use (vs. no metformin use) in relation with (a) delivery <37 weeks, (b) small for gestational age and (c) large for gestational age.

Included studies examined metformin (*n* = 6 studies), insulin (*n* = 7), other oral hypoglycaemic medication (*n* = 4) and other non‐diabetes medication (*n* = 2) as exposures. Meta‐analysis could be conducted for three studies reporting associations between metformin use and delivery <37 weeks, small for gestational age (SGA) and LGA (Figure 2).^22^, ^23^, ^24^ All other findings are summarized in Table 3, with detailed extracted data in Tables S5–S8.

**TABLE 3:** Summary of findings on associations between treatment and medication and maternal and child health outcomes.

Outcomes	Metformin	Insulin	Other oral hypoglycaemic medications	Other non‐diabetes medications
Weight/BMI at booking				
Excessive gestational weight gain	↓		↓	
Preeclampsia	↓; X	X		X
Hypertensive disorder of pregnancy	X	↑; X	X	X
Insulin dose				
Teratogenic medication use				
Glycaemia (achieving target control)				
Early booking appointment				
Stillbirth or miscarriage	↓		X	
Birth weight	X		X	X
High birth weight (macrosomia, LGA)	X		↑; X	X
Low birth weight (SGA)	X		X	X
Gestational age at delivery, preterm	X		X	X
Respiratory distress syndrome	X			
Caesarean section	↓; X	↓; X	↓	
Congenital anomaly	↓; X		↑; X	
Neonatal hypoglycaemia	X		X	
Neonatal jaundice				
APGAR score	X			
Special care nursery/NICU admission	X		X	
Shoulder dystocia			X	
Perinatal mortality	X			

*Note*: ↑, significant positive association; ↓, significant negative association; X, no significant association.

Abbreviations: APGAR (refers to the scoring system used by medical professionals to assess the health of a newborn baby shortly after birth and stands for Appearance, Pulse, Grimace, Activity, and Respiration); BMI, body mass index; LGA, large for gestational age; NICU, neonatal intensive care unit; SGA, small for gestational age.

##### Metformin

Six studies reported on associations between metformin and pregnancy, birth, delivery and neonatal outcomes.^22^, ^23^, ^25^, ^26^, ^27^, ^28^ Outcomes reported were related to neonatal intensive care unit (NICU) admission (*n* = 2), caesarean delivery (*n* = 2), hypertension/preeclampsia (*n* = 2), gestational age (*n* = 3), birthweight and growth‐related outcomes (*n* = 5) and other adverse fetal outcomes (*n* = 5).

###### Pregnancy and birth outcomes

Findings from meta‐analysis showed metformin use (vs. no metformin use) was not associated with delivery <37 weeks, SGA and LGA (Figure 2a–c).^22^, ^23^, ^24^ Similarly, no associations were reported for delivery <32 and < 34 weeks,^22^, ^25^ or for low or high birth weight.^25^

Findings from Lin et al. suggest metformin use may be associated with lower odds of stillbirth (OR 0.23; 95% CI 0.06–0.92), but no other studies examined this outcome.^25^ Racine et al. found metformin use was associated with lower odds of preeclampsia (OR 0.38; 95% CI 0.18–0.81),^22^ but this was not confirmed in any other study.^25^ Findings from one study suggested metformin (vs. insulin) was associated with lower GWG (median 3.5 kg vs. 7.1 kg, *p* = 0.008).^28^

###### Delivery outcomes

Lin et al. found metformin (vs. insulin) was associated with lower odds of caesarean delivery (OR 0.57; 95% CI 0.40–0.82),^25^ but this was not confirmed elsewhere.^23^

###### Neonatal outcomes

Lin et al. found metformin (vs. insulin) was associated with lower odds of congenital anomalies (OR 0.51; 95% CI 0.27–0.94),^25^ but no association was found in another study.^27^ No associations were found between metformin use and NICU admission, respiratory distress syndrome, neonatal hypoglycaemia, APGAR score and neonatal death.

##### Insulin

Seven studies reported on insulin treatment in relation to a wide range of outcomes.^25^, ^29^, ^30^, ^31^, ^32^, ^33^, ^34^ Outcomes reported related to caesarean delivery (*n* = 3), congenital anomalies (*n* = 2), GWG (*n* = 1), hypertensive disorders (*n* = 4), hypoglycaemia (*n* = 3), hyperglycaemia (*n* = 2), hyperbilirubinemia (*n* = 1), SGA/LGA (*n* = 4), perinatal death (*n* = 3), preterm (*n* = 3), respiratory distress (*n* = 2), shoulder dystocia (*n* = 2), NICU admission (*n* = 2), neonatal morbidity (*n* = 1), Apgar score (*n* = 1). However, only two significant associations were reported. Bartal et al. found basal insulin Neutral Protamine Hagedorn (NPH) vs. glargine, or detemir was associated with lower odds of caesarean delivery (OR 0.44; 95% CI 0.25–0.78)^29^; however, no association was observed in two other studies.^25^, ^30^

##### Other oral hypoglycaemic medications

Four studies reported on the use of oral hypoglycaemic medications other than metformin and insulin.^35^, ^36^, ^37^, ^38^ Outcomes reported were macrosomia (*n* = 2), congenital anomaly (*n* = 3) and a wide range of other maternal and infant outcomes including gestational age, birthweight, gestational weight gain, neonatal morbidities and mortality outcomes (*n* = 2). Findings from one study suggested glyburide use vs. no glyburide use may increase the odds of macrosomia (OR 1.93; 95% CI 1.51–2.47).^38^ Similarly, findings from one study showed oral hypoglycaemic use vs. no oral hypoglycaemic use was associated with higher odds of congenital anomaly (OR 1.8; 95% CI 1.0–3.3),^37^ but this was not confirmed in other studies.^35^, ^36^

Feghali et al. reported significant associations between the use of glyburide and metformin vs. insulin with caesarean delivery (OR 0.44; 95% CI 0.24–0.80) and excessive GWG (OR 0.44; 95% CI 0.24–0.80).^35^

No associations were reported for hypertensive disorders of pregnancy, preterm birth, birth weight, SGA, LGA, stillbirth, shoulder dystocia, NICU admission and neonatal hypoglycaemia.

##### Other non‐diabetes medications

Two studies reported on other medications, including aspirin, antidepressants, asthma and thyroid medications, for outcomes relating to gestational age (*n* = 2), birthweight and hypertensive disorders (*n* = 1).^39^, ^40^ No significant associations were reported.^39^, ^40^

#### 3.3.2 Healthcare

**FIGURE 3:**
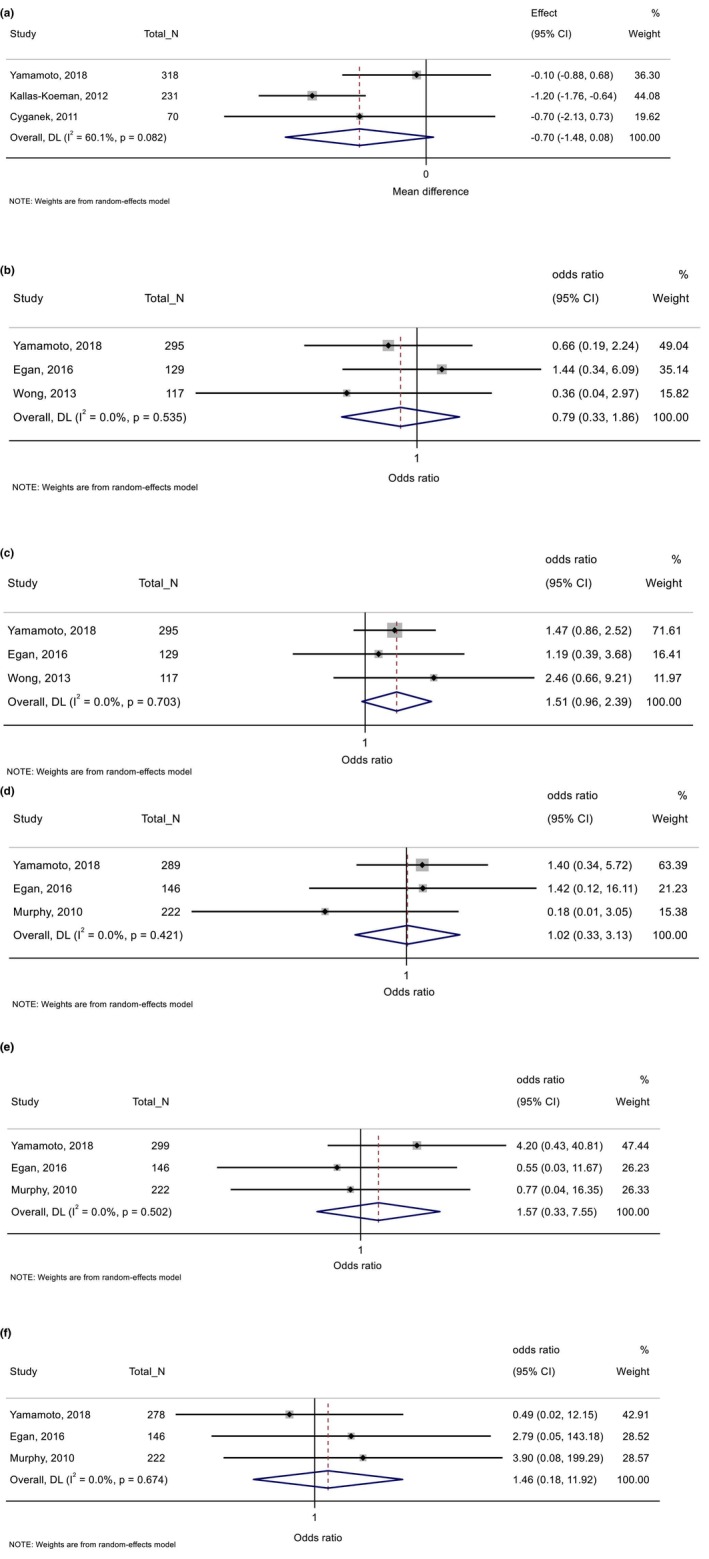
Preconception care (vs. no preconception care) in relation with (a) First‐trimester HbA1c (%), (b) delivery <34 weeks, (c) delivery <37 weeks, (d) congenital anomaly, (e) stillbirth and (f) neonatal death.

Studies examined healthcare‐related factors, including exposure to a PCC programme or specialist care (*n* = 8 studies), timing of presenting for antenatal care and exposure to additional antenatal programmes (*n* = 6) and planned caesarean section (*n* = 1). Meta‐analysis could only be conducted for six studies that reported on associations between PCC and first trimester HbA1c, delivery <34 and <37 weeks, congenital anomaly, stillbirth and neonatal death (Figure 3).^41^, ^42^, ^43^, ^44^, ^45^, ^46^ All other findings are summarized in Table 4, with detailed extracted data in Tables S9–S11.

**TABLE 4:** Summary of findings on associations between healthcare and maternal and child health outcomes.

Outcomes	Preconception care (attendance, specialist care)	Antenatal care (early attendance, additional intervention)	Planned caesarean section
Preconception folic acid supplement use	↑		
Vitamin D level	↑		
Smoking	X		
Weight/BMI at booking	X		
Gestational weight gain	↓	X	
Preeclampsia (term delivery)		↓; X	
Hypertensive disorder of pregnancy	X		
Insulin dose	X		
Teratogenic medication use	X		
Glycaemia (achieving target control)	↑; X	↑; X	
Early booking appointment	X		
Stillbirth or miscarriage	X		
Birth weight	X	X	
High birth weight (macrosomia, LGA)	X	↓; X	X
Low birth weight (SGA)	X	X	
Gestational age at delivery, preterm	X	↓; X	
Respiratory distress syndrome			↑
Caesarean section	X	X	
Congenital anomaly	X	X	
Neonatal hypoglycaemia	X		X
Neonatal jaundice		X	
Special care nursery/NICU admission	X	↓; X	X
Shoulder dystocia	X	X	X
Perinatal mortality	X	↓; X	

*Note*: ↑, significant positive association; ↓, significant negative association; X, no significant association.

Abbreviations: BMI, body mass index; LGA, large for gestational age; NICU, neonatal intensive care unit; SGA, small for gestational age.

##### Preconception care

Eight studies reported on associations between PCC and a range of outcomes related to HbA1c (*n* = 6), folic acid supplement use (*n* = 4), hypertensive disorders (*n* = 2), birthweight (*n* = 3), gestational age (*n* = 3), congenital anomalies (*n* = 4), mortality (*n* = 4), maternal BMI (*n* = 2) and other outcomes such as optimal pregnancy preparation and gestational age at booking (*n* = 5). Related exposures included attending PCC,^32^, ^42^, ^43^, ^44^, ^46^ exposure to community‐based PCC (vs. before introduction of the programme),^41^ and specialist diabetes care prior to pregnancy.^45^

###### Pregnancy and birth outcomes

Pooled findings showed no significant associations between PCC and first‐trimester HbA1c (*n* = 3 studies), although results pointed towards lower HbA1c among women who attended PCC (MD −0.70%, 95% CI −1.48, 0.08; *I* ^*2*^ = 60.1%, *p* = 0.08) (Figure 3a).^41^, ^42^, ^43^ This was observed in two studies which showed higher odds of meeting first‐trimester glycaemic targets among women who attended PCC.^41^, ^44^ PCC was also associated with higher odds of taking any dose of preconception folic acid,^41^, ^44^ and taking the UK‐recommended 5 mg dose.^41^, ^46^ Wong et al. found higher vitamin D levels and lower GWG among women who received specialist diabetes care during the 12 months before pregnancy.^45^ There were no significant associations reported for the timing of booking appointment, weight/BMI at booking, smoking, insulin or oral hypoglycaemic agent use, teratogenic medication use, hypertensive disorders of pregnancy and miscarriage.

Findings from the meta‐analysis showed no significant associations between PCC and preterm delivery <34 or < 37 weeks (*n* = 3 studies) (Figure 3b,c). There were also no significant associations reported between PCC and macrosomia, LGA, SGA, birthweight, shoulder dystocia or caesarean delivery, based on a limited number of small studies.

###### Neonatal outcomes

Pooled findings showed no significant associations between PCC and congenital anomaly, stillbirth and neonatal death (*n* = 3 studies) (Figure 3d–f). Other outcomes examined included neonatal hypoglycaemia and NICU admission, but no significant associations were reported.

##### Antenatal care

Six studies reported heterogeneous antenatal care‐related factors for outcomes related to preeclampsia (*n* = 3), caesarean (*n* = 3), preterm delivery (*n* = 4), birthweight (*n* = 4), shoulder dystocia (*n* = 2), mortality (*n* = 3), glycaemic control (*n* = 3), maternal weight (*n* = 1), NICU (*n* = 2), neonatal growth, insulin use, perinatal health and gestational age (*n* = 1). Allen et al. reported that no antenatal care (vs. presenting in the first trimester) was associated with higher odds of preeclampsia with delivery at term (OR 1.96; 95% CI 1.12–3.43), preterm delivery (OR 1.55; 95% CI 1.03–2.32) and intrauterine fetal death (OR 11.37; 95% CI 6.10–21.16).^47^ Similarly, Murphy et al. found women who attended antenatal care before vs. after 10 weeks' gestation had lower odds of late pregnancy HbA1c <6.5% (48 mmol/mol) (OR 0.77; 95% CI 0.66–0.91).^46^ Stafl et al. found women with one or more missed appointments (vs. no missed appointments) had higher odds of having an LGA infant (OR 1.61; 95% CI 1.13–2.28).^48^ Studies that compared intervention cohorts with historical reference cohorts found that motivational interviewing was associated with better glycaemic control outcomes,^34^ and medical nutrition therapy with lower odds of NICU admission (OR 0.21; 95% CI 0.03–0.51).^49^

##### Planned caesarean section

One study examined the association between planned caesarean section (vs. attempted vaginal delivery) and a range of birth and neonatal outcomes. Higher odds were observed for a composite neonatal outcome (OR 1.37; 95% CI 1.07–1.75) and respiratory distress syndrome (OR 6.13; 95% CI 1.76–21.32), but not for other outcomes such as neonatal hypoglycaemia and NICU admission.

#### 3.3.3 Health conditions

There were 21 studies reporting on maternal BMI (including higher BMI, underweight, overweight and obesity), 11 on GWG, 20 on glycaemia, three on blood pressure and one on anxiety. Meta‐analysis could only be conducted for five studies that reported on associations between GWG and SGA and LGA.^26^, ^50^, ^51^, ^52^, ^53^ All other findings are summarized in Table 5, with detailed extracted data presented in Tables S12–S16.

**TABLE 5:** Summary of findings on associations between health conditions and maternal and child health outcomes.

Outcomes	BMI				GWG		Glycaemic control	Blood pressure	Anxiety or depressive symptoms
	Higher BMI	Underweight	Overweight	Obesity	Excessive/above guideline	Below guideline			
Gestational weight gain			X	↑; X					X
Preeclampsia			X	↑; X	X		X		
Hypertension	↑		X	↑			↑; X		
Insulin dose	X		X	X	↑			X	X
Glycaemia (achieving target control)	↓	X	X	↓	X				↑
Missing ≥1 antenatal appointments	↑								
Progression of retinopathy	X						X	X	
Stillbirth or miscarriage							↑; X		
Birth weight			X	↑	↑; X				
High birth weight (macrosomia, LGA)	↑; X		X	↑; X	↑; X		↑; X		X
Low birth weight (SGA)	X		X	X	X		X		X
Gestational age at delivery, preterm			X	X	↑; X		↑; X	X	X
Caesarean section	X		X	X	↑; X	↑; X	X		X
Congenital anomaly	↑		X	X			↑; X		X
Neonatal hypoglycaemia				↑	↑		↔; X		X
Excessive fetal adiposity				↑					
Neonatal jaundice			X	X	X		X		X
Special care nursery/NICU admission			↑	↑	X		X		
Perinatal mortality					X		X		

*Note*: ↑, significant positive association; ↓, significant negative association; X, no significant association.

Abbreviations: BMI, body mass index; GWG, gestational weight gain; LGA, large for gestational age; NICU, neonatal intensive care unit; SGA, small for gestational age.

##### Maternal BMI

###### Pregnancy and birth outcomes

Among two studies reporting on obesity and GWG, one reported no association,^54^ and one reported higher odds of excessive GWG for women with obesity compared to those with normal weight (OR 4.92; 95% CI 1.43–16.94).^55^ Similarly, one of three studies reporting on obesity found increased risk of preeclampsia for women with vs. without obesity (RR 2.25; 95% CI 1.20–4.22).^56^ Only one small study reported higher odds of hypertension in pregnancy (OR 9.7; 95% CI 2.3–40.8) and pregnancy‐induced hypertension (OR 9.3; 95% CI 1.1–80.9) for women with obesity compared to those with normal weight.^54^

Three studies reported on glycaemia‐related outcomes,^4^, ^55^, ^57^ showing higher BMI among women not within target HbA1c compared to women within target (34.0 vs. 31.9 kg/m^2^, *p* < 0.001).^57^ While underweight and overweight were not associated with target HbA1c, women with obesity were less likely to achieve optimal glycaemia (OR ranging from 0.52 to 0.65 depending on obesity class).^57^

Associations between maternal BMI (or weight) and treatment and care‐related outcomes were examined in six studies.^30^, ^48^, ^55^, ^58^, ^59^, ^60^ Findings from Stafl et al. suggested women with a preconception weight ≥ 91 kg vs. <91 kg had higher odds of missing one or more antenatal appointments (OR 1.53; 95% CI 1.16–2.03).^48^ No associations were found between maternal BMI and insulin dose, caesarean section and progression of retinopathy.

Among seven studies reporting on birthweight‐related outcomes,^24^, ^26^, ^52^, ^53^, ^54^, ^55^, ^61^ one found a higher birthweight among infants of women with obesity compared to women with a normal weight (3525 g vs. 2870 g, *p* = 0.01).^54^ Studies also reported higher odds of macrosomia (>4 kg)^54^ and LGA^53^ for women with obesity compared to normal weight, while no associations were found between BMI and SGA.

###### Neonatal outcomes

Seven studies reported on various neonatal health outcomes,^37^, ^54^, ^55^, ^56^, ^62^, ^63^ with one showing higher odds of congenital anomaly among women with a higher BMI (OR 1.09; 95% CI 1.01–1.18),^37^ although no associations were found for overweight and obesity in another study.^54^ Kapustin et al. found a higher risk of neonatal hypoglycaemia (RR 3.37; 95% CI 1.27–8.99) and excessive fetal adiposity (RR 1.83; 95% CI 1.07–2.12) for women with vs. without obesity,^56^ but no associations were found for neonatal jaundice.^55^ Abell et al. found higher odds of NICU admission for infants of women with overweight (OR 6.14; 95% CI 1.19–31.81) and obesity (OR 3.69; 95% CI 1.05–12.99) compared to women with normal weight.

##### Gestational weight gain

Associations between GWG and maternal and child health‐related outcomes included birthweight (*n* = 7), preeclampsia (*n* = 3), caesarean (*n* = 3), gestational age (*n* = 3), growth (*n* = 2), mortality (*n* = 3), NICU (*n* = 2), other perinatal outcomes (*n* = 3) and diabetes control‐related outcomes (*n* = 3).

###### Pregnancy and birth outcomes

**FIGURE 4:**
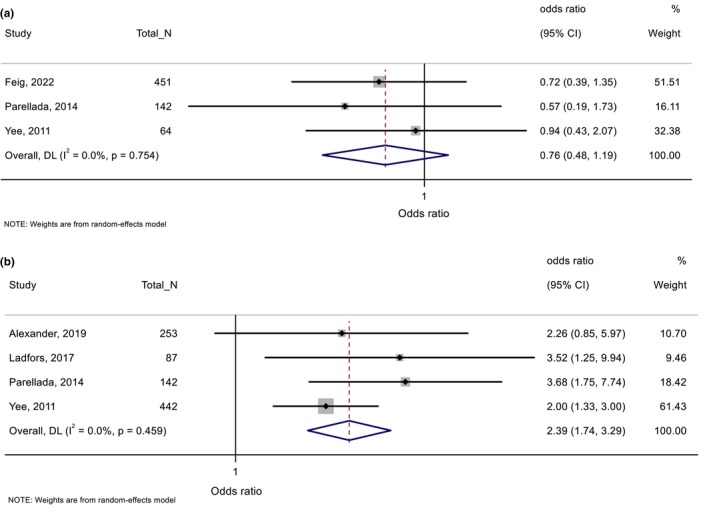
Excessive gestational weight gain (vs. no excessive gestational weight gain) in relation with (a) small for gestational age and (b) large for gestational age.

Pooled findings from three studies showed no significant association between excessive GWG (vs. no excessive GWG) and SGA (OR 0.76; 95% CI 0.48–1.19), but higher odds of LGA were observed based on pooled findings from four studies (OR 2.39; 95% CI 1.74–3.29) with no heterogeneity (*I* ^*2*^ = 0.0%, *p* = 0.46) (Figure 4a,b). Excessive or higher GWG was also associated with higher odds of macrosomia (>4 kg) or higher birthweight in some,^50^, ^51^ but not all studies.^34^, ^61^ Based on two studies examining the association between GWG and insulin dose in late pregnancy, findings suggest that excessive GWG was associated with a higher dose.^34^, ^50^ No significant associations were found between excessive or total GWG and preeclampsia or HbA1c in late pregnancy.

###### Delivery outcomes

Weight change less than or greater than the Institute of Medicine (IOM) guidelines (vs. within the guidelines) was associated with higher odds of caesarean section (OR 1.47; 95% CI 1.03–2.10 and 1.62; 1.03–2.57, respectively) based on one study,^51^ but this was not confirmed in other studies.^34^, ^50^ Excessive GWG was associated with higher odds of preterm delivery (<37 weeks)^39^ and earlier gestational age at delivery^34^ based on two studies, but not in a third study looking at similar outcomes.^50^

###### Neonatal outcomes

Two small studies reported that excessive vs. no excessive GWG was associated with higher odds of neonatal hypoglycaemia (OR 2.31; 95% CI 1.06–4.99 and 4.33; 1.06–17.57).^34^, ^50^ No significant associations were found between excessive or total GWG and length at birth or ponderal index, perinatal mortality, NICU admission or jaundice.

##### Glycaemic control

Associations between glycaemic control and maternal and child health outcomes included caesarean (*n* = 2), congenital anomaly (*n* = 5), hypertensive disorders (*n* = 5), induction and jaundice (*n* = 1), LGA (*n* = 7), neonatal hyperglycaemia (*n* = 4), NCU (*n* = 1), perinatal complications (*n* = 1) and perinatal death (*n* = 3), polyhydramnios (*n* = 1), preterm (*n* = 4), progression of retinopathy (*n* = 1) and SGA (*n* =  3).

###### Pregnancy and birth outcomes

Higher HbA1c in pregnancy was associated with gestational hypertension in some,^32^, ^58^ but not all studies,^54^, ^64^ and no significant associations were found with preeclampsia.^58^, ^64^, ^65^ The odds of preterm birth were higher for women with poor first trimester glycaemia based on findings from one study,^4^ but this was not observed in other studies.^39^, ^58^, ^64^

Ten studies reported inconsistent findings on the association between a range of glycaemia measures assessed at different time points before and during pregnancy and LGA. Higher preconception and early pregnancy HbA1c were associated with higher odds of LGA in some,^4^, ^56^, ^66^ but not all studies.^24^, ^52^, ^53^ Late pregnancy HbA1c was not associated with LGA in most studies, and no associations were found for SGA or the progression of retinopathy.

###### Delivery outcomes

No significant associations were reported between glycaemic control and caesarean section, or induction of labour.^58^

###### Neonatal outcomes

Five studies reported associations between measures of glycaemia and a range of congenital anomaly outcomes.^4^, ^58^, ^64^, ^67^, ^68^ Of these, two reported higher odds of congenital anomalies among neonates born to mothers with HbA1c above target,^4^, ^68^ while no association was found in other studies.^58^, ^64^, ^67^ Findings on glycaemia and neonatal hypoglycaemia reported in three studies were inconsistent, showing early and late pregnancy higher HbA1c were associated with higher or lower odds of neonatal hypoglycaemia,^58^, ^66^ or no association.^69^ While two studies found no association between glycaemic measures and perinatal death,^58^ or miscarriage,^64^ one study found first‐trimester HbA1c >53 mmol/mol was associated with a composite outcome of adverse outcomes including stillbirth, neonatal death and congenital anomalies.^4^ No significant associations were reported between glycaemic control and jaundice or NICU admission.

##### Blood pressure

Three studies reported no significant associations between maternal blood pressure and chronic hypertension and preterm birth,^39^ insulin dose at delivery,^30^ or progression of retinopathy.^59^

##### Anxiety or depressive symptoms

Findings from one study suggest late pregnancy HbA1c might be slightly higher among women with anxiety or depressive symptoms (43 mmol/mol) compared with women with no such symptoms (40 mmol/mol, *p* = 0.04).^34^

#### 3.3.4 Health behaviours

Health behaviours reported included smoking (*n* = 10 studies), folic acid supplement use (*n* = 2) and breastfeeding (*n* = 2). Meta‐analysis could not be conducted, and the findings are summarized in Table 6, with detailed extracted data presented in Tables S17–S19.

**TABLE 6:** Summary of findings on associations between health behaviours and maternal and child health outcomes.

Outcomes	Smoking	Folic acid supplement use	Breastfeeding
Preeclampsia	X		
Insulin dose	X		
Missing ≥1 antenatal appointments	↑		
Progression of retinopathy	X		
High birth weight (macrosomia, LGA)	X		
Low birth weight (SGA)	↑; X		
Gestational age at delivery, preterm	X		
Congenital anomaly		X	
Child weight/BMI			X

*Note*: ↑, significant positive association; ↓, significant negative association; X, no significant association.

Abbreviations: BMI, body mass index; LGA, large for gestational age; SGA, small for gestational age.

##### Smoking

Ten studies reported on associations between smoking during pregnancy and a range of pregnancy and birth outcomes including birthweight‐related outcomes (*n* = 3) and other outcomes (*n* = 8).^24^, ^26^, ^30^, ^39^, ^48^, ^53^, ^54^, ^59^, ^65^, ^70^ Smoking was associated with SGA in one study (OR 2.50; 95% CI 1.06–5.92),^24^ but not in another.^26^ Stafl et al. found higher odds of missing one or more antenatal appointments among women who smoked during pregnancy compared with those who did not (OR 2.15; 95% CI 1.35–3.41).^48^ No associations were found for LGA, preeclampsia, preterm birth, progression of retinopathy and insulin dose.

##### Folic acid supplement use

Among the two studies that reported on the association between folic acid supplement use (5 mg or no dose specified), no significant associations were found in relation to congenital anomaly.^4^, ^37^

##### Breastfeeding

One study examined the association between breastfeeding at 6 months and child weight and BMI at 14 months, but found no significant associations.^71^ Another study reported on breastfeeding intention during pregnancy and showed higher odds of breastfeeding initiation and exclusive breastfeeding at discharge among women who had these respective intentions during pregnancy.^72^

## 4 DISCUSSION

This study explored associations between modifiable treatment, healthcare, health conditions and behavioural exposures in the preconception, pregnancy and postnatal periods, and clinical outcomes for women with T2D and their children. Access to PCC, maternal BMI, glycaemia and GWG are important risk factors in pregnancies affected by T2D. Interventions targeting these priority areas have the greatest potential to improve outcomes. However, most data addressed risk factors in pregnancy; few studies examined PCC and research into postnatal care was virtually absent, calling for new well‐powered studies to improve care.

### 4.1 Strengths and limitations

Our systematic review addresses a clinically relevant question and was conducted by a multidisciplinary team providing complementary expertise. Comprehensive search strategies were used to identify eligible studies following a robust methodological approach and prospective protocol registration. However, our study results are limited by the paucity of high‐quality data in this field. While there was a high number of included studies overall, these were heterogeneous in their reporting of both exposures and outcomes. Meta‐analysis and narrative synthesis were both reliant on a limited number of studies for each combination of exposure and outcome, with frequent reporting from a single study. Therefore, patterns in results should be interpreted with caution, and more studies are required to explore whether the associations remain in more diverse populations drawn from varied geographical and healthcare contexts. Studies are conducted among largely white populations, and more diverse data and predefined sub‐analyses based on ethnicity are also needed to examine the impacts of risk factors on maternal and child health outcomes for minoritized ethnic groups who are disproportionately affected by T2D. Most studies were underpowered to identify associations with perinatal outcomes, especially less common outcomes such as stillbirth or congenital anomalies. Conflicting results from different studies limited our ability to make definitive conclusions. The observational nature of the included studies also has limitations, such as residual confounding, and findings cannot be interpreted as causal effects. Lastly, publication bias may have been introduced through the inclusion of studies only published in the English language.

### 4.2 Comparison with other studies

#### 4.2.1 Preconception

While PCC is widely considered to be beneficial, there is very little evidence available to guide optimal PCC in women with T2D. A previous systematic review of PCC in pregestational type 1 or 2 diabetes highlighted the lack of clear evidence of efficacy.^73^ The authors concluded that PCC was effective at reducing congenital anomalies by 71% but had little or no effect on other outcomes. They identified that PCC *may* reduce HbA1c concentrations in the first trimester and reduce risks for preterm delivery, perinatal mortality, NICU admission and SGA.^73^ The review did not identify preconception intervention studies conducted among women with T2D,^73^ similar to our more recent review.^13^

Our results provide more support for PCC. We found that PCC was associated with increased prescription of folic acid 5 mg and vitamin D and may lead to lower weight gain in pregnancy. PCC also provides opportunities to address maternal BMI, associated with multiple suboptimal outcomes in pregnancy, including congenital anomaly, preeclampsia, hypertensive disorders, LGA, neonatal hypoglycaemia and NICU admission. However, very little evidence exists about how to improve and maintain optimal maternal BMI before pregnancy, and how much weight should be lost. Although there are new medications such as semaglutide and tirzepatide to support weight loss in people with T2D, these have not been assessed in the preconception period due to concerns of use whilst attempting to conceive.

While more evidence around PCC would be desirable, effective implementation of accepted standard care is also key; measures such as using folic acid 5 mg and removing teratogenic medications are widely recommended^12^ and strongly beneficial at a population level, yet implementation is not widespread. A key area of unmet need therefore relates to access and uptake of PCC in women with T2D, especially in under‐represented groups.^4^ PCC is not equitably accessible to women from lower socioeconomic status, with lower education level, language barriers and from minoritized ethnic backgrounds. New studies to improve access to PCC, raise awareness of the benefits of family planning and address reasons for declining contraception are all urgently needed.

#### 4.2.2 Pregnancy

Our results suggest that in pregnancy, maternal HbA1c and GWG are the two most important modifiable risk factors for suboptimal outcomes in T2D pregnancies.

HbA1c was included in multiple studies in this systematic review, but was too heterogeneous for a meta‐analysis. Narrative synthesis identified probable associations between higher HbA1c and congenital anomaly, stillbirth, preeclampsia, preterm delivery, neonatal death, LGA and hypertensive disorders, with some inconsistencies between different studies. However, larger studies have shown strong and consistent associations between higher HbA1c and suboptimal outcomes. For example, UK NPID audit data in women with T1D and T2D identified third‐trimester HbA1c, living in the highest deprivation quintile and having T2D as key risk factors for perinatal death in pregnancy.^4^

Optimizing maternal HbA1c clearly remains crucial in the management of T2D in pregnancy, but there was insufficient evidence to recommend specific treatment strategies. Metformin was associated with reduced GWG but did not show consistent benefits upon preterm birth, SGA or LGA in this meta‐analysis of observational studies. However, interventional studies have shown some benefits of metformin use.^13^ For example, the metformin in women with T2D in pregnancy (MiTy) trial demonstrated that while metformin did not improve a composite primary outcome including fetal and neonatal outcomes when compared to placebo in individuals treated with insulin, it did reduce GWG, improve maternal glycaemia and reduce rates of LGA.^74^ Boggess and colleagues studied metformin use in a mixed cohort of women with T2D and women with GDM diagnosed early in pregnancy. Treatment with metformin did not affect rates of composite adverse neonatal outcomes, but the metformin group had fewer LGA infants.^75^ Meta‐analysis data showed that metformin use in pregnancies with T2D and GDM reduced GWG and rates of LGA, gestational hypertension, neonatal hypoglycaemia and NICU admission, but increased rates of SGA.^76^

GWG was the only risk factor with a definite association in this review, with higher GWG being associated with LGA and preterm delivery. However, there are no studies assessing interventions primarily designed to improve GWG in pregnancies with T2D or recommending targets to optimize outcomes in this group. Observational and interventional evidence suggest that metformin can reduce GWG, but the evidence was inconsistent for benefits on outcomes. Studies to assess other interventions to reduce GWG or to define optimal targets for GWG in women with T2D in pregnancy, are needed to improve evidence in this area.

### 4.3 Implications and future research

Our study highlights the importance of PCC, but there is virtually no evidence about what PCC should be offered for T2D, how maternal BMI and glycaemia should be improved and how services should be structured to improve access to women with T2D, who often come from under‐represented groups.^77^ There remains marked evidence gaps in this area that need to be urgently addressed.

In pregnancies affected by T2D, maternal BMI, GWG and glycaemia are key determinants of outcomes. Currently, pregnancy care for women with T2D focuses on improving glycaemia through behavioural measures and medications such as metformin and insulin^12^ and screening for complications such as preeclampsia. Our review suggests we need to broaden that perspective to optimize clinical care; for example, by focussing more on GWG management and long‐term measures to improve BMI.

Focussing more on GWG raises several important questions. Firstly, what is the optimal way to define excessive GWG in the context of T2D? Excessive GWG is typically defined according to the IOM guidelines, which are not tailored for the population of women with T2D. There have been historical barriers to considering weight loss in pregnancy, but recent work suggests that reduced weight gain or even weight loss, might be beneficial in women with obesity or GDM.^78^, ^79^, ^80^ Further research is needed to assess optimal GWG for women with T2D, and to assess if weight loss in pregnancy improves outcomes. Secondly, what interventions to target GWG can make a meaningful difference in outcomes? Our results suggest that metformin can reduce GWG in T2D, consistent with work in other populations of pregnant women with diabetes. However, although metformin has some clear benefits, it may not be sufficiently effective on GWG to improve outcomes. Other interventions to address GWG in women with T2D have rarely been attempted. Alongside pharmacological strategies, new, innovative and non‐stigmatizing approaches that address socioeconomic and societal barriers need to be co‐developed to support women optimize diet and physical activity before, during and after pregnancy.

## 5 CONCLUSION

Based on observational evidence synthesized in this systematic review, increasing access to PCC could be beneficial to optimize maternal nutrition and weight‐related outcomes, and addressing obesity and GWG has the potential to improve maternal and neonatal outcomes in pregnancies affected by T2D. To most effectively and efficiently achieve improved maternal and child outcomes, future research should address research gaps related to these three key areas.

## FUNDING INFORMATION

DS is supported by the National Institute for Health and Care Research (NIHR) through an NIHR Advanced Fellowship (NIHR302955) and the NIHR Southampton Biomedical Research Centre (NIHR203319). SLW is supported by a grant from the Medical Research Council (UK) (MR/W003740/1). CLM is supported by Diabetes UK through a Harry Keen Intermediate Clinical Fellowship (17/0005712) and the European Foundation for the Study of Diabetes—Novo Nordisk Foundation Future Leaders' Award (NNF19SA058974) and the NIHR Leicester Biomedical Research Centre. NH is supported by the National Institute for Health and Care Research (NIHR) Academy through an NIHR Career Development Fellowship (CDF‐2018‐11‐ST2‐011).

## CONFLICT OF INTEREST STATEMENT

The authors have no conflicts of interest to disclose.

## Supporting information


**Table S1.** Study participant characteristics of included studies (*N* = 58).
**Table S2.** Methodological characteristics of included studies (*N* = 58).
**Table S3.** Newcastle–Ottawa Scale (NOS) quality assessment guide.
**Table S4.** Quality assessment results using the Newcastle–Ottawa Scale (NOS).
**Table S5.** Findings on associations between metformin and maternal and child health outcomes.
**Table S6.** Findings on associations between insulin and maternal and child health outcomes.
**Table S7.** Findings on associations between other oral hypoglycaemic medications and maternal and child health outcomes.
**Table S8.** Findings on associations between other non‐diabetes medications and maternal and child health outcomes.
**Table S9.** Findings on associations between preconception care and maternal and child health outcomes.
**Table S10.** Findings on associations between antenatal care and maternal and child health outcomes.
**Table S11.** Findings on associations between planned c‐section and maternal and child health outcomes.
**Table S12.** Findings on associations between maternal BMI and maternal and child health outcomes.
**Table S13.** Findings on associations between gestational weight gain and maternal and child health outcomes.
**Table S14.** Findings on associations between glycaemic control and maternal and child health outcomes.
**Table S15.** Findings on associations between blood pressure and maternal and offspring health outcomes.
**Table S16.** Findings on associations between anxiety and/or depressive symptoms and maternal and child health outcomes.
**Table S17.** Findings on associations between smoking and maternal and child health outcomes.
**Table S18.** Findings on associations between folic acid supplement use and maternal and child health outcomes.
**Table S19.** Findings on associations between breastfeeding and maternal and child health outcomes.

**File S1.** Search strategies.

## References

[1] Lascar N , Brown J , Pattison H , Barnett AH , Bailey CJ , Bellary S . Type 2 diabetes in adolescents and young adults. Lancet Diabetes Endocrinol. 2018;6(1):69‐80. 28847479 10.1016/S2213-8587(17)30186-9

[2] NHS Digital . National Pregnancy in Diabetes Audit Dashboard 2023 (01 January 2021 to 31 December 2023). Accessed November 11, 2024. https://digital.nhs.uk/data‐and‐information/publications/statistical/national‐pregnancy‐in‐diabetes‐audit/2023

[3] Blankstein AR , Sigurdson SM , Frehlich L , et al. Pre‐existing diabetes and stillbirth or perinatal mortality: a systematic review and meta‐analysis. Obstet Gynecol. 2024;144(5):608‐619. 39088826 10.1097/AOG.0000000000005682

[4] Murphy HR , Howgate C , O'Keefe J , et al. Characteristics and outcomes of pregnant women with type 1 or type 2 diabetes: a 5‐year national population‐based cohort study. Lancet Diabetes Endocrinol. 2021;9(3):153‐164. 33516295 10.1016/S2213-8587(20)30406-X

[5] Meek CL . An unwelcome inheritance: childhood obesity after diabetes in pregnancy. Diabetologia. 2023;66(11):1961‐1970. 37442824 10.1007/s00125-023-05965-w PMC10541526

[6] Immanuel J , Flack J , Wong VW , et al. The ADIPS pilot National Diabetes in pregnancy benchmarking Programme. Int J Environ Res Public Health. 2021;18(9):4899. 34064492 10.3390/ijerph18094899 PMC8125192

[7] Sargeant JA , Brady EM , Zaccardi F , et al. Adults with early‐onset type 2 diabetes (aged 18‐39 years) are severely underrepresented in diabetes clinical research trials. Diabetologia. 2020;63(8):1516‐1520. 32483683 10.1007/s00125-020-05174-9 PMC7351852

[8] Lv X , Ran X , Chen X , et al. Early‐onset type 2 diabetes: a high‐risk factor for proliferative diabetic retinopathy (PDR) in patients with microalbuminuria. Medicine (Baltimore). 2020;99(19):e20189. 32384512 10.1097/MD.0000000000020189 PMC7220424

[9] Nanayakkara N , Curtis AJ , Heritier S , et al. Impact of age at type 2 diabetes mellitus diagnosis on mortality and vascular complications: systematic review and meta‐analyses. Diabetologia. 2021;64(2):275‐287. 33313987 10.1007/s00125-020-05319-w PMC7801294

[10] Barker MM , Davies MJ , Sargeant JA , et al. Age at type 2 diabetes diagnosis and cause‐specific mortality: observational study of primary care patients in England. Diabetes Care. 2023;46(11):1965‐1972. 37625035 10.2337/dc23-0834

[11] Dibato JE , Montvida O , Zaccardi F , et al. Association of Cardiometabolic Multimorbidity and Depression with Cardiovascular Events in early‐onset adult type 2 diabetes: a multiethnic study in the U.S. Diabetes Care. 2021;44(1):231‐239. 33177170 10.2337/dc20-2045

[12] National Institute for Health and Care Excellence (NICE) . Diabetes in pregnancy: management from preconception to the postnatal period. NICE guideline [NG3]. Published: 25 February 2015, last updated: 16 December 2020. Accessed November 11, 2024. https://www.nice.org.uk/guidance/ng3

[13] Gunabalasingam S , Kyrka A , Hopkins L , et al. Interventions in women with type 2 diabetes mellitus in the pre‐pregnancy, pregnancy and postpartum periods to optimise care and health outcomes: a systematic review. Diabet Med. 2025;42(1):e15474. 39527377 10.1111/dme.15474 PMC11635590

[14] Hopkins L , O'Leary N , Burton A , et al. Experiences of women with type 2 diabetes during the pre‐pregnancy, pregnancy, and postpartum periods: a systematic review of qualitative studies. [under review]. 10.1111/dme.70094 PMC12352727 40607686

[15] Moher D , Liberati A , Tetzlaff J , Altman DG . Preferred reporting items for systematic reviews and meta‐analyses: the PRISMA statement. PLoS Med. 2009;6(7):e1000097. 19621072 10.1371/journal.pmed.1000097 PMC2707599

[16] Haddaway NR , Grainger MJ , Gray CT . citationchaser: An R package and Shiny app for forward and backward citations chasing in academic searching (0.0.3). 2021. 10.1002/jrsm.1563 35472127

[17] Covidence Systematic Review Software, Veritas Health Innovation. www.covidence.org

[18] Higgins JP , Thomas J , Chandler J , et al. Cochrane Handbook for Systematic Reviews of Interventions. John Wiley & Sons; 2019. doi:10.1002/9781119536604 PMC10284251 31643080

[19] Rothstein HR , Sutton AJ , Borenstein M . Publication Bias in Meta‐Analysis: Prevention, Assessment and Adjustments. John Wiley & Sons; 2006. doi:10.1002/0470870168

[20] Popay J , Roberts H , Sowden A , et al. Guidance on the conduct of narrative synthesis in systematic reviews. A Product from the ESRC Methods Programme Version. 2006;1(1):b92.

[21] Wells G , Shea B , O'Connell D , et al. The Newcastle‐Ottawa Scale (NOS) for assessing the quality of nonrandomised studies in meta‐analyses. 2013 Accessed July 4, 2024. https://www.ohri.ca/programs/clinical_epidemiology/oxford.asp

[22] Racine JL , Adams JH , Antony KM , et al. Metformin exposure and risk of hypertensive disorders of pregnancy in patients with type 2 diabetes. Am J Perinatol. 2021;38(11):1103‐1108. 33940652 10.1055/s-0041-1728821

[23] Maple‐Brown LJ , Lindenmayer G , Barzi F , et al. Real‐world experience of metformin use in pregnancy: observational data from the Northern Territory diabetes in pregnancy clinical register. J Diabetes. 2019;11(9):761‐770. 30680949 10.1111/1753-0407.12905

[24] Rowan JA , Luen S , Hughes RC , Sadler LC , McCowan LM . Customised birthweight centiles are useful for identifying small‐for‐gestational‐age babies in women with type 2 diabetes. Aust N Z J Obstet Gynaecol. 2009;49(2):180‐184. 19432607 10.1111/j.1479-828X.2009.00975.x

[25] Lin SF , Chang SH , Kuo CF , Lin WT , Chiou MJ , Huang YT . Association of pregnancy outcomes in women with type 2 diabetes treated with metformin versus insulin when becoming pregnant. BMC Pregnancy Childbirth. 2020;20(1):512. 32887578 10.1186/s12884-020-03207-0 PMC7487639

[26] Feig DS , Zinman B , Asztalos E , et al. Determinants of small for gestational age in women with type 2 diabetes in pregnancy: who should receive metformin? Diabetes Care. 2022;45(7):1532‐1539. 35671033 10.2337/dc22-0013

[27] Kjerpeseth LJ , Cesta CE , Furu K , et al. Metformin versus insulin and risk of major congenital malformations in pregnancies with type 2 diabetes: a Nordic register‐based cohort study. Diabetes Care. 2023;46(8):1556‐1564. 37343541 10.2337/dc23-0256

[28] Soepnel LM , Nicolaou V , Huddle KRL , Klipstein‐Grobusch K , Levitt NS , Norris SA . Maternal and neonatal outcomes following a diabetic pregnancy within the context of HIV. Int J Gynaecol Obstet. 2019;147(3):404‐412. 31479156 10.1002/ijgo.12956

[29] Fishel Bartal M , Ward C , Refuerzo JS , et al. Basal insulin analogs versus neutral protamine Hagedorn for type 2 diabetics. Am J Perinatol. 2020;37(1):30‐36. 31430822 10.1055/s-0039-1694733

[30] Nadeau HCG , Maxted ME , Madhavan D , Pierce SL , Feghali M , Scifres C . Insulin dosing, glycemic control, and perinatal outcomes in pregnancies complicated by Type‐2 diabetes. Am J Perinatol. 2021;38(6):535‐543. 33065743 10.1055/s-0040-1718579

[31] Rowe CW , Watkins B , Brown K , et al. Efficacy and safety of the pregnancy‐IVI, an intravenous insulin protocol for pregnancy, following antenatal betamethasone in type 1 and type 2 diabetes. Diabet Med. 2021;38(4):e14489. 33277738 10.1111/dme.14489

[32] Morikawa M , Kato‐Hirayama E , Mayama M , et al. Glycemic control and fetal growth of women with diabetes mellitus and subsequent hypertensive disorders of pregnancy. PLoS One. 2020;15(3):e0230488. 32176740 10.1371/journal.pone.0230488 PMC7075561

[33] Kapustin RV , Kopteeva EV , Alekseenkova EN , Tsybuk EM , Arzhanova ON , Kogan IY . Risk factors for shoulder dystocia during labor in women with diabetes mellitus. Obstet Gynecol. 2022;(9):54‐63. doi:10.18565/aig.2022.9.54-63

[34] Asbjörnsdóttir B , Rasmussen SS , Kelstrup L , Damm P , Mathiesen ER . Impact of restricted maternal weight gain on fetal growth and perinatal morbidity in obese women with type 2 diabetes. Diabetes Care. 2013;36(5):1102‐1106. 23248191 10.2337/dc12-1232 PMC3631818

[35] Feghali MN , Caritis SN , Catov JM , Scifres CM . Glycemic control and pregnancy outcomes in women with type 2 diabetes treated with Oral hypoglycemic agents. Am J Perinatol. 2017;34(7):697‐704. 27984840 10.1055/s-0036-1597625

[36] Cesta CE , Rotem R , Bateman BT , et al. Safety of GLP‐1 receptor agonists and other second‐line antidiabetics in early pregnancy. JAMA Intern Med. 2023;184(2):144‐152. 10.1001/jamainternmed.2023.6663 PMC10714281 38079178

[37] Roland JM , Murphy HR , Ball V , Northcote‐Wright J , Temple RC . The pregnancies of women with type 2 diabetes: poor outcomes but opportunities for improvement. Diabet Med. 2005;22(12):1774‐1777. 16401329 10.1111/j.1464-5491.2005.01784.x

[38] Shannon MH , Wintfeld N , Liang M , Jovanovic L . Pregnancy snapshot: a retrospective, observational case‐control study to evaluate the potential effects of maternal diabetes treatment during pregnancy on macrosomia. Curr Med Res Opin. 2016;32(7):1183‐1192. 26958899 10.1185/03007995.2016.1164128

[39] Søholm JC , Vestgaard M , Ásbjörnsdóttir B , et al. Potentially modifiable risk factors of preterm delivery in women with type 1 and type 2 diabetes. Diabetologia. 2021;64(9):1939‐1948. 34146144 10.1007/s00125-021-05482-8

[40] Do NC , Vestgaard M , Ásbjörnsdóttir B , et al. Unchanged prevalence of preeclampsia after implementation of prophylactic aspirin for all pregnant women with preexisting diabetes: a prospective cohort study. Diabetes Care. 2021;44:2252‐2259. 10.2337/dc21-1182 34400481

[41] Yamamoto JM , Hughes DJF , Evans ML , et al. Community‐based pre‐pregnancy care programme improves pregnancy preparation in women with pregestational diabetes. Diabetologia. 2018;61(7):1528‐1537. 29744539 10.1007/s00125-018-4613-3 PMC6445478

[42] Kallas‐Koeman MM , Khandwala F , Donovan LE . Rate of preconception Care in Women with type 2 diabetes still lags behind that of women with type 1 diabetes. Can J Diabetes. 2012;36:170‐174.

[43] Cyganek K , Hebda‐Szydlo A , Skupien J , et al. Glycemic control and pregnancy outcomes in women with type 2 diabetes from Poland. The impact of pregnancy planning and a comparison with type 1 diabetes subjects. Endocrine. 2011;40(2):243‐249. 21528433 10.1007/s12020-011-9475-0

[44] Egan AM , Danyliv A , Carmody L , Kirwan B , Dunne FP . A Prepregnancy care program for women with diabetes: effective and cost saving. J Clin Endocrinol Metab. 2016;101(4):1807‐1815. 26918293 10.1210/jc.2015-4046

[45] Wong VW , Suwandarathne H , Russell H . Women with pre‐existing diabetes under the care of diabetes specialist prior to pregnancy: are their outcomes better? Aust N Z J Obstet Gynaecol. 2013;53(2):207‐210. 23452190 10.1111/ajo.12044

[46] Murphy HR , Roland JM , Skinner TC , et al. Effectiveness of a regional prepregnancy care program in women with type 1 and type 2 diabetes: benefits beyond glycemic control. Diabetes Care. 2010;33(12):2514‐2520. 21115765 10.2337/dc10-1113 PMC2992180

[47] Allen AJ , Snowden JM , Lau B , Cheng Y , Caughey AB . Type‐2 diabetes mellitus: does prenatal care affect outcomes? J Matern Fetal Neonatal Med. 2018;31(1):93‐97. 28076991 10.1080/14767058.2016.1276558

[48] Stafl L , Benham JL , Frehlich L , Donovan LE , Yamamoto JM . Missed antenatal diabetes care appointments and neonatal outcomes for pregnancies with type 1 and type 2 diabetes. Diabet Med. 2023;40(1):e14950. 36054517 10.1111/dme.14950

[49] Perichart‐Perera O , Balas‐Nakash M , Parra‐Covarrubias A , et al. A medical nutrition therapy program improves perinatal outcomes in Mexican pregnant women with gestational diabetes and type 2 diabetes mellitus. Diabetes Educ. 2009;35(6):1004‐1013. 19696205 10.1177/0145721709343125

[50] Parellada CB , Asbjörnsdóttir B , Ringholm L , Damm P , Mathiesen ER . Fetal growth in relation to gestational weight gain in women with type 2 diabetes: an observational study. Diabet Med. 2014;31(12):1681‐1689. 25081349 10.1111/dme.12558 PMC4257095

[51] Yee LM , Cheng YW , Inturrisi M , Caughey AB . Effect of gestational weight gain on perinatal outcomes in women with type 2 diabetes mellitus using the 2009 Institute of Medicine guidelines. Am J Obstet Gynecol. 2011;205(3):257.e1‐257.e6. 10.1016/j.ajog.2011.06.028 PMC3425437 22071055

[52] Alexander LD , Tomlinson G , Feig DS . Predictors of large‐for‐gestational‐age birthweight among pregnant women with type 1 and type 2 diabetes: a retrospective cohort study. Can J Diabetes. 2019;43(8):560‐566. 31677906 10.1016/j.jcjd.2019.08.015

[53] Ladfors L , Shaat N , Wiberg N , Katasarou A , Berntorp K , Kristensen K . Fetal overgrowth in women with type 1 and type 2 diabetes mellitus. PLoS One. 2017;12(11):e0187917. 29121112 10.1371/journal.pone.0187917 PMC5679529

[54] Colatrella A , Braucci S , Festa C , et al. Hypertensive disorders in normal/over‐weight and obese type 2 diabetic pregnant women. Exp Clin Endocrinol Diabetes. 2009;117(8):373‐377. 19536738 10.1055/s-0029-1220763

[55] Nørgaard SK , Do NC , Ásbjörnsdóttir B , et al. Prepregnancy body mass index and offspring birth weight in women with type 1 and type 2 diabetes. J Preg Child Health. 2016;3(2):244‐255.

[56] Kapustin R , Kopteeva E , Tiselko A , et al. Diabetes and pregnancy study (DAPSY): a 10‐year single‐center cohort study of pregnancies affected by diabetes. Arch Gynecol Obstet. 2023;309(6):2643‐2651. 37594491 10.1007/s00404-023-07187-2

[57] Murphy HR , Bell R , Cartwright C , et al. Improved pregnancy outcomes in women with type 1 and type 2 diabetes but substantial clinic‐to‐clinic variations: a prospective nationwide study. Diabetologia. 2017;60(9):1668‐1677. 28597075 10.1007/s00125-017-4314-3 PMC5552835

[58] Abell SK , Boyle JA , de Courten B , et al. Contemporary type 1 diabetes pregnancy outcomes: impact of obesity and glycaemic control. Med J Aust. 2016;205(4):162‐167. 27510344 10.5694/mja16.00443

[59] Rasmussen KL , Laugesen CS , Ringholm L , Vestgaard M , Damm P , Mathiesen ER . Progression of diabetic retinopathy during pregnancy in women with type 2 diabetes. Diabetologia. 2010;53(6):1076‐1083. 20225131 10.1007/s00125-010-1697-9

[60] Isabey EP , Pylypjuk CL . The relationship between fetal Abdominal Wall thickness and intrapartum complications amongst mothers with Pregestational type 2 diabetes. J Diabetes Res. 2021;2021:5544599. 34195292 10.1155/2021/5544599 PMC8184339

[61] Olmos PR , Araya‐Del‐Pino AP , González‐Carvello CA , et al. Near‐optimal glycemic control in Chilean women with pregestational type‐2 diabetes: persistent macrosomia relates to maternal pre‐pregnancy overweight. Diabetes Res Clin Pract. 2009;85(1):53‐60. 19446354 10.1016/j.diabres.2009.04.015

[62] Endo S , Saisho Y , Miyakoshi K , et al. Association of Maternal Factors with perinatal complications in pregnancies complicated with diabetes: a single‐center retrospective analysis. J Clin Med. 2018;7(1):5. 29301307 10.3390/jcm7010005 PMC5791013

[63] Rais R , Starikov R , Robert W , Has P , He M . Clinicopathological correlation of large‐for‐gestational age placenta in pregnancies with pregestational diabetes. Pathol Res Pract. 2019;215(3):405‐409. 30616883 10.1016/j.prp.2018.12.029

[64] Owens LA , Sedar J , Carmody L , Dunne F . Comparing type 1 and type 2 diabetes in pregnancy‐ similar conditions or is a separate approach required? BMC Pregnancy Childbirth. 2015;15:69. 25885892 10.1186/s12884-015-0499-y PMC4390076

[65] Oppermann M , Alessi J , Hirakata VN , Wiegand DM , Reichelt AJ . Preeclampsia in women with pregestational diabetes ‐ a cohort study. Hypertens Pregnancy. 2020;39(1):48‐55. 31875734 10.1080/10641955.2019.1704002

[66] McLean A , Barr E , Tabuai G , Murphy HR , Maple‐Brown L . Continuous glucose monitoring metrics in high‐risk pregnant women with type 2 diabetes. Diabetes Technol Ther. 2023;25(12):836‐844. 37902969 10.1089/dia.2023.0300 PMC10698759

[67] Arendt LH , Pedersen LH , Pedersen L , et al. Glycemic control in pregnancies complicated by pre‐existing diabetes mellitus and congenital malformations: a Danish population‐based study. Clin Epidemiol. 2021;13:615‐626. 34345185 10.2147/CLEP.S298748 PMC8325058

[68] Ahmed M , Mujeeb‐Ur‐Rehman Abro S , Khuhro BN , Bhanbhro FI , Shaikh A . Risk of congenital anomalies in pregnant women with type 2 diabetes. Pak J Med Health Sci. 2022;16(11):141.

[69] Yamamoto JM , Donovan LE , Mohammad K , Wood SL . Severe neonatal hypoglycaemia and intrapartum glycaemic control in pregnancies complicated by type 1, type 2 and gestational diabetes. Diabet Med. 2020;37(1):138‐146. 31529717 10.1111/dme.14137 PMC6916340

[70] Kekki M , Tihtonen K , Salonen A , et al. Severe birth injuries in neonates and associated risk factors for injury in mothers with different types of diabetes in Finland. Int J Gynaecol Obstet. 2022;159(1):195‐203. 34927725 10.1002/ijgo.14073 PMC9545198

[71] Longmore DK , Titmuss A , Barr E , et al. Breastfeeding and infant growth in offspring of mothers with hyperglycaemia in pregnancy: the pregnancy and neonatal diabetes outcomes in remote Australia study. Pediatr Obes. 2022;17(6):e12891. 35187835 10.1111/ijpo.12891

[72] Cordero L , Stenger MR , Landon MB , Nankervis CA . Impact of excessive gestational weight gain on exclusive breastfeeding among women with type 1 and type 2 diabetes and obesity. PLoS One. 2022;17(11):e0277599. 36395288 10.1371/journal.pone.0277599 PMC9682946

[73] Wahabi HA , Fayed A , Esmaeil S , et al. Systematic review and meta‐analysis of the effectiveness of pre‐pregnancy care for women with diabetes for improving maternal and perinatal outcomes. PLoS One. 2020;15(8):e0237571. 32810195 10.1371/journal.pone.0237571 PMC7433888

[74] Feig DS , Donovan LE , Zinman B , et al. Metformin in women with type 2 diabetes in pregnancy (MiTy): a multicentre, international, randomised, placebo‐controlled trial. Lancet Diabetes Endocrinol. 2020;8(10):834‐844. 32946820 10.1016/S2213-8587(20)30310-7

[75] Boggess KA , Valint A , Refuerzo JS , et al. Metformin plus insulin for preexisting diabetes or gestational diabetes in early pregnancy: the MOMPOD randomized clinical trial. JAMA. 2023;330(22):2182‐2190. 38085312 10.1001/jama.2023.22949 PMC10716718

[76] He K , Guo Q , Ge J , Li J , Li C , Jing Z . The efficacy and safety of metformin alone or as an add‐on therapy to insulin in pregnancy with GDM or T2DM: a systematic review and meta‐analysis of 21 randomized controlled trials. J Clin Pharm Ther. 2022;47(2):168‐177. 34363237 10.1111/jcpt.13503

[77] Goldney J , Alabraba V , Sarkar P , et al. Designing a regional clinical service for people with early‐onset type 2 diabetes in England. Diabet Med. 2024;42:e15479. 39587392 10.1111/dme.15479 PMC11929562

[78] Voerman E , Santos S , Inskip H , et al. Association of Gestational Weight Gain with Adverse Maternal and Infant Outcomes. JAMA. 2019;321(17):1702‐1715. 31063572 10.1001/jama.2019.3820 PMC6506886

[79] Johansson K , Bodnar LM , Stephansson O , Abrams B , Hutcheon JA . Safety of low weight gain or weight loss in pregnancies with class 1, 2, and 3 obesity: a population‐based cohort study. Lancet. 2024;403(10435):1472‐1481. 38555927 10.1016/S0140-6736(24)00255-1 PMC11097195

[80] Kusinski LC , Jones D , Atta N , et al. Reduced energy diet in women with gestational diabetes: the dietary intervention (DiGest) randomised clinical trial. Nat Med. 2025;31(2):514‐523. 39972237 10.1038/s41591-024-03356-1 PMC11839452

[81] Abell SK , Boyle JA , de Courten B , et al. Impact of type 2 diabetes, obesity and glycaemic control on pregnancy outcomes. Aust N Z J Obstet Gynaecol. 2017;57(3):308‐314. 27593528 10.1111/ajo.12521

[82] Alrais M , Ward C , Cornthwaite JAA , et al. Type 2 diabetes and neonatal hypoglycemia: role of route of delivery and insulin infusion. J Matern Fetal Neonatal Med. 2022;35(25):7445‐7451. 34344270 10.1080/14767058.2021.1949452

[83] Ásbjörnsdóttir B , Vestgaard M , Ringholm L , et al. Effect of motivational interviewing on gestational weight gain and fetal growth in pregnant women with type 2 diabetes. BMJ Open Diabetes Res Care. 2019;7(1):e000733. 10.1136/bmjdrc-2019-000733 PMC6861008 31798895

[84] Ásbjörnsdóttir B , Vestgaard M , Do NC , et al. Prevalence of anxiety and depression symptoms in pregnant women with type 2 diabetes and the impact on glycaemic control. Diabet Med. 2021;38(3):e14506. 33368557 10.1111/dme.14506

[85] Gaudio M , Dozio N , Feher M , et al. Trends in factors affecting pregnancy outcomes among women with type 1 or type 2 diabetes of childbearing age (2004‐2017). Front Endocrinol (Lausanne). 2020;11:596633. 33692751 10.3389/fendo.2020.596633 PMC7937966

[86] Persson M , Cnattingius S , Wikström AK , Johansson S . Maternal overweight and obesity and risk of pre‐eclampsia in women with type 1 diabetes or type 2 diabetes. Diabetologia. 2016;59(10):2099‐2105. 27369871 10.1007/s00125-016-4035-z PMC5016540

[87] Sushko K , Menezes HT , Butt M , et al. Trends and self‐management predictors of glycemic control during pregnancy in women with preexisting type 1 or type 2 diabetes: a cohort study. Diabetes Spectr. 2023;36(2):182‐192. 37193202 10.2337/ds22-0046 PMC10182963

