# Supplementary material for: Associations of modifiable preconception, pregnancy and postpartum factors with health outcomes for women with type 2 diabetes and their children: A systematic review and meta‐analysis of observational studies
Source: Diabet Med. 2025 Dec 7;43(2):e70183. doi: 10.1111/dme.70183 (PMC12857875; doi:10.1111/dme.70183)
Supplement: Supplementary file 1 — Table S1. Study participant characteristics of included studies (N = 58). Table S2. Methodological characteristics of included studies (N = 58). Table S3. Newcastle–Ottawa Scale (NOS) quality assessment guide. Table S4. Quality assessment results using the Newcastle–Ottawa Scale (NOS). Table S5. Findings on associations between metformin and maternal and child health outcomes. Table S6. Findings on associations between insulin and maternal and child health outcomes. Table S7. Findings on associations between other oral hypoglycaemic medications and maternal and child health outcomes. Table S8. Findings on associations between other non‐diabetes medications and maternal and child health outcomes. Table S9. Findings on associations between preconception care and maternal and child health outcomes. Table S10. Findings on associations between antenatal care and maternal and child health outcomes. Table S11. Findings on associations between planned c‐section and maternal and child health outcomes. Table S12. Findings on associations between maternal BMI and maternal and child health outcomes. Table S13. Findings on associations between gestational weight gain and maternal and child health outcomes. Table S14. Findings on associations between glycaemic control and maternal and child health outcomes. Table S15. Findings on associations between blood pressure and maternal and offspring health outcomes. Table S16. Findings on associations between anxiety and/or depressive symptoms and maternal and child health outcomes. Table S17. Findings on associations between smoking and maternal and child health outcomes. Table S18. Findings on associations between folic acid supplement use and maternal and child health outcomes. Table S19. Findings on associations between breastfeeding and maternal and child health outcomes. [file DME-43-e70183-s001.docx]

**Supplementary Tables**

|  |  | **Page** |
| --- | --- | --- |
| **Supplementary Table 1**. Study participant characteristics of included studies (N = 58) |  | 2 |
|  |  |  |
| **Supplementary Table 2**. Methodological characteristics of included studies (N = 58) |  | 7 |
|  |  |  |
| **Supplementary Table 3**. Newcastle - Ottawa Scale (NOS) quality assessment guide |  | 14 |
|  |  |  |
| **Supplementary table 4**. Quality assessment results using the Newcastle - Ottawa Scale (NOS) |  | 16 |
|  |  |  |
| **Supplementary Table 5**. Findings on associations between metformin and maternal and child health outcomes |  | 18 |
|  |  |  |
| **Supplementary Table 6**. Findings on associations between insulin and maternal and child health outcomes |  | 24 |
|  |  |  |
| **Supplementary Table 7**. Findings on associations between other oral hypoglycaemic medications and maternal and child health outcomes |  | 32 |
|  |  |  |
| **Supplementary Table 8**. Findings on associations between other non-diabetes medications and maternal and child health outcomes |  | 35 |
|  |  |  |
| **Supplementary Table 9**. Findings on associations between preconception care and maternal and child health outcomes |  | 37 |
|  |  |  |
| **Supplementary Table 10**. Findings on associations between antenatal care and maternal and child health outcomes |  | 52 |
|  |  |  |
| **Supplementary Table 11**. Findings on associations between planned c-section and maternal and child health outcomes |  | 62 |
|  |  |  |
| **Supplementary Table 12**. Findings on associations between maternal BMI and maternal and child health outcomes |  | 63 |
|  |  |  |
| **Supplementary Table 13**. Findings on associations between gestational weight gain and maternal and child health outcomes |  | 71 |
|  |  |  |
| **Supplementary Table 14**. Findings on associations between glycaemic control and maternal and child health outcomes |  | 79 |
|  |  |  |
| **Supplementary Table 15**. Findings on associations between blood pressure and maternal and offspring health outcomes |  | 94 |
|  |  |  |
| **Supplementary Table 16**. Findings on associations between anxiety and/or depressive symptoms and maternal and child health outcomes |  | 95 |
|  |  |  |
| **Supplementary Table 17**. Findings on associations between smoking and maternal and child health outcomes |  | 97 |
|  |  |  |
| **Supplementary Table 18**. Findings on associations between folic acid supplement use and maternal and child health outcomes |  | 99 |
|  |  |  |
| **Supplementary Table 19**. Findings on associations between breastfeeding and maternal and child health outcomes |  | 100 |
|  |  |  |
| Reference |  | 101 |

**Supplementary Table 1**. Study participant characteristics of included studies (N = 58)

| **First author, year** | **Data collection period** | **Age (years)** | **Ethnicity** | **Diabetes duration (years)** | **BMI (kg/m^2^)** |
| --- | --- | --- | --- | --- | --- |
| Colatrella, 2009 | 1998-2005 | Mean ± SD: 32.7 ± 4.6 | All White | Mean ± SD: 5.7 ± 4.5 | Mean ± SD: 28.3 ± 6.3 |
| Cyganek, 2011 | 1999-2009 | Mean; 95% CI: 33.1; 31.9-34.4 | NR | Mean; 95% CI: 3.3; 2.4-4.3 | Mean; 95% CI: 30.8; 29.2-32.5 |
| Murphy, 2017 | 2015 | Mean ± SD: 33.6 ± 5.2 | 46% White, 28% Asian, 9% Black, 5% Mixed, 12% Unknown | Mean ± SD: 4.8 ±4.3 | Mean ± SD: 33.3 ±7.3 |
| Morikawa, 2020 | 2011-2018 | Mean ± SD: 33.5 ± 0.5 | NR | NR | Mean ± SD: 28.9 ± 0.5 |
| Rasmussen, 2010 | 2003-2008 | Mean ± SD: 32.5 ± 5.3 in no progression group; 33.0 ± 5.8 in progression group | NR | Mean ± SD: 3.3 ± 2.8 in no progression group; 6.7 ± 4.6 in progression group. | Mean ± SD: 32.4 ± 7.3 in no progression group; 30.7 ± 4.6 in progression group |
| Roland, 2005 | 1999-2004 | Mean ± SD: 33.9 ± 5.2 | 58.9% White, 28.7% Asian, 10.9% other | NR | Mean ± SD: 34.2 ± 7.3 |
| Owens, 2015 | 2007-NR | Mean ± SD: 33.7 ± 4.8 | 70% White | Mean ± SD: 4.3 ± 3.8 | Mean ± SD: 34.9 ± 6.7 |
| Yamamoto, 2020 | 2007-2014 | Mean ± SD: 33.7 ± 5.0 | NR | Mean ± SD: 4.0 ± 4.2 | NR |
| Parellada, 2014 | 2008-2013 | Median (range): 34 (20-45) | 57% White Nordic; 22% Middle Eastern; 10% African; 8% Asian; 3% other | Median (range): 3 (0.2-11) | Median (range) 32 (19-48) in excessive weight gain group; 29.7 (20-53) in non-excessive weight gain group |
| Shannon, 2016 | 2005-2011 | Mean ± SD: 34.0 ± 4.5 | NR | NR | NR |
| Gaudio, 2020 | 2004-2017 | Mean ± SD: 31.0 ± 7.9 in year 2017 | 54% White in year 2017 | NR | NR |
| Isabey, 2021 | 2014-2018 | Mean ± SD: 31.1 ± 6.5 | NR | NR | Mean: 36.6. 96.8% overweight or obesity |
| Persson, 2016 | 1997-2012 | NR | NR | NR | NR |
| Ladfors, 2017 | 2006-2016 | Mean ± SD: 34.7 ± 4.9 | 65% White Nordic | NR | Median (IQR): 31.1 (27.1-36.1) |
| Maple-Brown, 2019 | 2012-2016 | Mean ± SD: 29.1 ± 7.0 in Indigenous women/no treatment group; 31.1 ± 5.7 in Indigenous women/treatment group; 30.0 ± 6.7 in non-Indigenous women/no treatment group; 35.4 ± 3.9 in non-Indigenous women/treatment group | NR | NR | Mean ± SD: 31.7 ± 5.3 in Indigenous women/no treatment group; 31.5 ± 5.8 in Indigenous women/treatment group; 30.9 ± 8.6 in non-Indigenous women/no treatment group; 30.5 ± 6.9 in non-Indigenous women/treatment group |
| Alexander, 2019 | 2009-2016 | Mean ± SD: 35.1 ± 5.1 | 26.3% White | Mean ± SD: 5.4 ± 5.1 | NR |
| Abell, 2017 | 2010-2013 | Mean ± SD: 33.6 ± 5.3 | NR | NR | Median (IQR): 32.9 (26.2-36.9) |
| Allen, 2018 | 1997-2006 | NR | NR | NR | NR |
| Arendt, 2021 | 2000-2015 | NR | NR | NR | NR |
| Endo, 2018 | 2008-2015 | Mean ± SD: 38.0 ± 2.7 | NR | Mean ± SD: 3.6 ± 4.2 | Mean ± SD: 27.5 ± 4.9 |
| Kekki, 2021 | 2004-2017 | Mean ± SD: 32.6 ± 5.7 | NR | NR | Mean ± SD: 31.8 ± 7.50 |
| Lin, 2020 | 2003-2014 | NR | NR | NR | NR |
| Kallas-Koeman,2012 | 2006-2010 | Mean ± SD: 32.7 ± 5.5 in no preconception care group; 33.7 ± 4.1 in preconception care group | NR | Mean ± SD: 3.4 ± 3.4 in no preconception care group; 3.3 ± 3.6 in preconception care group | NR |
| Murphy, 2021 | 2014-2018 | Median (10^th^- 90^th^ percentile): 34 (27-41) | 43.2% White; 35.6% Asian; 9.6% Black; 2.5% Mixed; 2.9% Other; 6.2% Unknown | Median (10^th^- 90^th^ percentile): 3 (0-10) | Median (10^th^- 90^th^ percentile): 32.5 (24.8-43.0) |
| Oppermann, 2020 | 2005-2015 | Mean ± SD: 33.1 ± 5.8 in women with no preeclampsia; 34.1 ± 4.4 in women with preeclampsia | 73% White in women with no preeclampsia  91% White in women with preeclampsia | Mean ± SD: 3.9 ± 5.0 in women with no preeclampsia; 5.3 ± 5.0 in women with preeclampsia | NR |
| Rais, 2019 | 2003-2011 | Mean ± SD: 32.9 ± 5.8 in appropriate for gestational age placenta group; 32.3 ± 6.0 in large for gestational age placenta group | NR | NR | Mean ± SD: 37.2 ± 7.7 in appropriate for gestational age placenta group; 38.1 ± 9.2 in large for gestational age placenta group |
| Rowan, 2009 | 1998-2003 | Mean ± SD: 32.8 ± 4.7 | 51% Pacific Islanders, 20% European, 11% Chinese/other Southeast Asian, 10% Indian and 8% Māori | NR | Mean ± SD: 34.3 ± 7.2 |
| Rowe, 2021 | 2014-2020 | Mean ± SD: 32 ± 5.8 | NR | Median (IQR): 4.0 (1- 8) | Median (IQR): 35.5 (31.5 - 42.6) |
| Søholm, 2021 | 2015-2018 | Mean ± SD: 34 ± 5 for term delivery group; 32 ± 6 for preterm delivery group | 57% North-European origin for term delivery group; 53% North-European origin for preterm delivery group | Mean ± SD: 4.2 ± 4 for term delivery group; 3.0 ± 2 for preterm delivery group | Mean ± SD: 32.9 ± 7 for term delivery group; 32 ± 4 for preterm delivery group |
| Yee, 2011 | 2001-2004 | NR | 13% White, 6% Black, 71% Latina, 10% Asian | NR | NR |
| Soepnel, 2019 | 2012-2018 | Mean ± SD: 34 ± 5.2 | Predominantly Black African | NR | Mean ± SD: 35.1 ± 8.1 |
| Asbjornsdottir, 2021 | 2015-2018 | Mean ± SD: 34 ± 5 | 58% North-European | Median (IQR): 3 (1.4-8) | Median (IQR): 32.1 (27.5-37.4) |
| Asbjornsdottir, 2013 | 2008-2011 | Median (range): 35 (23-44) in weight gain ≤ 5 kg group and 34 (20-45) in weight gain >5 kg group | 71% White Nordic | Median (range): 3 (0.25-10) in weight gain ≤ 5 kg group and 3 (0.5-10) in weight gain >5 kg group | Median (range): 33.5 (30-52.7) in weight gain ≤ 5 kg group and 36.8 (30-48.2) in weight gain >5 kg group |
| Feig, 2022 | 2011-2018 | Mean ± SD: 34.9 ± 4.8 | 79% non-European | NR | Mean ± SD: 33.8 ± 7.3 |
| Kapustin, 2023 | 2008-2017 | Mean; 95% CI: 33.7; 33.1-34.4 | NR | Median (IQR): 3 (1-7) | Mean; 95% CI: 30.4, 9.7-31.1 |
| Kapustin, 2022 | 2008-2017 | Mean ± SD: 33.6 ± 5.3 in diet group; 24.0 ± 5.2 in insulin group | NR | NR | Mean ± SD: 33.8 ±6.6 in diet group; 33.8 ±6.2 in insulin group |
| Kjerpeseth, 2023 | 1996-2020 | NR | NR | NR | NR |
| Longmore, 2022 | 2011-2017 | NR | Majority Aboriginal or Torres Strait Islanders | NR | NR |
| Ahmed, 2022 | 2021-2022 | Mean ± SD: 31 ± 5.5 | NR | NR | Mean ± SD: 35.1 ± 8.9 |
| Alrais, 2022 | 2012-2018 | NR | 28% Non-Hispanic White, 35% Non-Hispanic Black, 25% Hispanic, 1% Non-Hispanic Other, 10% Unknown | NR | NR |
| Cordero, 2022 | 2013-2020 | Median (IQR): 33 (29-37) | 39% White, 24% African American, Hispanic 26%, Other 11% | NR | Median (IQR): 39 (24-45) |
| Stafl, 2023 | 2008-2020 | Mean ± SD: 33.3 ± 5.4 in missed 1 or more appointments group; 34.5 ± 4.8 in no missed appointments group | NR | Mean ± SD: 4.1 ± 3.9 in missed 1 or more appointments group; 3.8 ± 3.8 in no missed appointments group | NR |
| Sushko, 2023 | 2014-2019 | Mean ± SD: 31.0 ± 5.2 | 74.1% European, 9.3% Indigenous, 7.4% South Asian | Mean ± SD: 5.1 ± 5.4 | NR |
| Cesta, 2023 | 2009-2021 | NR | NR | NR | NR |
| Nørgaard, 2016 | 2012-2014 | NR | 53% White Nordic | Mean ± SD: 4.5 ± 4.0 | Mean ± SD: 31.8±6.5 |
| McLean, 2023 | 2019-2021 | Mean ± SD: 33.2 ± 5.5 | 74% Aboriginal or Torres Strait Islanders | NR | Mean ± SD: 32.8 ± 5.9 |
| Olmos, 2009 | 1997-2007 | Mean ± SEM: 32.9 ± 0.7 | NR | Mean ± SEM: 2.1 ± 0.5 | Mean ± SEM: 31.5 ± 0.8 |
| Feghali, 2017 | 2009-2012 | Mean ± SD: 32.8 ± 5.1 in oral hypoglycaemic agents group; 33.3 ± 5.8 in insulin group | 58% White, 30% Black and 8% Other in oral hypoglycaemic agents group; 69% White, 28% Black and 5% Other in insulin group | NR | NR |
| Racine, 2021 | 2009-2019 | Mean ± SD: 33 ± 5.3 in metformin group; 32.2 ± 5.1 in no metformin group | 65.8% White, 16.1% Black, 9.4% Asian, 6.3% Multiracial | NR | Mean ± SD: 37.5 ± 10.9 in metformin group; 34.8 ±8.4 in no metformin group |
| Bartal, 2019 | 2012-2018 | NR | 27% Non-Hispanic White; 36.9% Non-Hispanic Black; 24.9% Hispanic; 1.3% non-Hispanic other; 10.3% unknown | NR | NR |
| Wong, 2013 | 2007-2011 | Mean ± SD: 34 ± 5.3 in under specialist care group; 34.7 ± 5 in no specialist care group | NR | Mean ± SD: 4.9 ± 3.9 in under specialist care group; 3.6 ± 3.8 in no specialist care group | Mean ± SD: 31.8 ± 5.6 in under specialist care group; 32.9 ± 7.6 in no specialist care group |
| Egan, 2016 | 2006-2014 | Mean ± SD: 33.3 ± 3.5 in pre-pregnancy care programme group; 33.7 ± 5.3 in no pre-pregnancy care programme group | 84.2% White, 15.8% Other in pre-pregnancy care programme group; 67.3% White, 32.7% Other in no pre-pregnancy care programme group | Mean ± SD: 2.8 ± 2.5 in pre-pregnancy care programme group; 2.8 ± 4.3 in no pre-pregnancy care programme group | Mean ± SD: 33.6 ± 5.8 in pre-pregnancy care programme group; 33.7 ± 7.1 in no pre-pregnancy care programme group |
| Yamamoto, 2018 | 2013-2017 | Mean ± SD: 34.7 ± 5.2 before pre-pregnancy care programme; 33.4 ± 5.4 during/after pre-pregnancy care programme | NR | NR | Mean ± SD: 34.3 ± 8.0 before pre-pregnancy care programme; 33.9 ± 7.2 during/after pre-pregnancy care programme |
| Murphy, 2010 | 2006-2009 | Median (10^th^- 90^th^ percentile): 35 (27-39) in pre-pregnancy care programme group; 34 (25-40) in no pre-pregnancy care programme group | 83.0% White in pre-pregnancy care programme group; 54.6% White in no pre-pregnancy care programme group | NR | Median (10^th^- 90^th^ percentile): 33.9 (26-40) in pre-pregnancy care programme group; 32.2 (24-43) in no pre-pregnancy care programme group |
| Ásbjörnsdóttir, 2019 | 2013-2018 | Mean ± SD: 34 ± 5 in intervention group; 34 ± 6 in reference group | 58% North European origin in intervention group; 48% in reference group | Median (IQR): 3(1-8) in intervention group; 2.5 (0.5-5) in reference group | Mean ± SD: 32.8 ± 6.9 in intervention group; 32.4 ± 7.4 in reference group |
| Do, 2021 | 2015-2020 | Mean ± SD: 32 ± 5 | 77% Northern European origin | Median (IQR): 11 (4-18) | Median (IQR): 26.3 (23.1-31.5) |
| Perichart-Perera, 2009 | 2001-2007 | NR | NR | NR | NR |
| Nadeau, 2021 | 2015-2017 | Mean ± SD: 29.1 ± 6.4 in >2 units insulin/kg dose at delivery group; 30.6 ± 5.5 in <2 units insulin/kg dose at delivery group | 29% White; 13% Black; 41% Hispanic; 15% Native American; 2% Asian | NR | NR |

Note: Study participant characteristics are described for women with type 2 diabetes within each study.

Abbreviations: BMI (Body Mass Index); IQR (inter-quartile range); NR (not reported); SD (standard deviation); SEM (standard error of the mean); T1D (type 1 diabetes); T2D (type 2 diabetes).

**Supplementary Table 2**. Methodological characteristics of included studies (N = 58)

| **First author, year** | **Inclusion criteria** | **Exclusion criteria** | **Study only includes T2D participants** | **Timeframe(s) of exposure(s)^a^** | **Timeframe(s) of outcome(s)^a^** |
| --- | --- | --- | --- | --- | --- |
| Colatrella, 2009 | Caucasian pregnant patients with T2D in outpatient clinic, with a diabetes duration longer than one year | Twin pregnancies | Yes | Pregnancy | Pregnancy |
| Cyganek, 2011 | Consecutive singleton pregnancies in Caucasian women with pregestational diabetes in South-Eastern Poland | Incomplete follow-up (i.e. not from booking until delivery), multiple pregnancy | No | Preconception | Pregnancy |
| Murphy, 2017 | Pregnancies with pregestational diabetes | Diabetes diagnosed during pregnancy, uncertain about type of diabetes, missing type of diabetes data | No | Pregnancy | Pregnancy |
| Morikawa, 2020 | Women who gave birth at or after 22 weeks gestation and had T2D; (including overt diabetes in pregnancy) | Fetal chromosome abnormality, and twins and triplets | No | Pregnancy | Pregnancy |
| Rasmussen, 2010 | Singleton pregnancies in women with T2D referred before 20 weeks | Multiple pregnancy, delivery before 22 weeks; referred after 20 weeks; no medical record; less than two ophthalmological examinations performed during pregnancy | Yes | Preconception, pregnancy | Pregnancy |
| Roland, 2005 | Pregnancies in women with T2D | Miscarriages and terminations for other reasons (not congenital anomaly) | No | Pregnancy | Pregnancy |
| Owens, 2015 | Women with T2D for more than six months prior to index pregnancy | Multiple pregnancy | No | Pregnancy | Pregnancy |
| Yamamoto, 2020 | Women with T2D who attended specialised interdisciplinary clinics | Delivered outside the study area, had an unclear definition of type of diabetes, had an unknown expected date of confinement, or delivered after the study period | No | Pregnancy | Pregnancy |
| Parellada, 2014 | Women with pregestational T2D, referred before 21 weeks, singleton pregnancy | Previous bariatric surgery, thyrotoxicosis, Crohn's disease and missing weight gain data | Yes | Pregnancy | Pregnancy, postpartum |
| Shannon, 2016 | Pregnant women with live birth & diagnosis of T2D on two distinct episodes | Unclear diagnosis | No | Pregnancy | Pregnancy |
| Gaudio, 2020 | Women with T2D aged 16 to 45 years registered with a GP in the Royal College of General Practitioners Research and Surveillance Centre network | Pregnant during the study period, menopausal (premature ovarian failure or surgical menopause) | No | Preconception | Preconception |
| Isabey, 2021 | Women with T2D delivering at single hospital in 5-year period | Cases of multiples, congenital anomalies, planned postnatal palliation, planned delivery by Caesarean section, and those delivering prior to 36 weeks, no stored fetal ultrasound images from a 35- to 36-week scan | Yes | Pregnancy | Pregnancy |
| Persson, 2016 | Pregnant women with T2D | Missing personal identification numbers, no information on country of birth, multiple births, births with unknown gestational age, women with gestational diabetes | No | Pregnancy | Pregnancy |
| Ladfors, 2017 | Women with T2D and singleton pregnancies attending a two-site maternity service for whom medical records for antenatal care and delivery were available | Multiple gestations, miscarriages, intrauterine fetal deaths, major congenital anomalies | No | Pregnancy | Pregnancy |
| Maple-Brown, 2019 | Pregnant women residing in the Northern Territory (Australia) with T2D | None reported. | No | Pregnancy | Pregnancy |
| Alexander, 2019 | Women with T2D with live singleton pregnancies, ≥28 weeks gestation, medical records available | Women with multiple gestations, spontaneous abortion (fetal loss before 20 weeks) or intrauterine fetal death | No | Pregnancy | Pregnancy |
| Abell, 2017 | Women with singleton births from 20 weeks gestation at Monash Health in Victoria, Australia | Women with T1D and gestational diabetes | Yes | Preconception, pregnancy | Pregnancy |
| Allen, 2018 | Women with diabetes or T2D, delivered singleton pregnancies between 24 and 41 weeks gestation | Diagnoses codes of T1D and gestational diabetes, multiple gestations | Yes | Pregnancy | Pregnancy |
| Arendt, 2021 | Women with singleton children born in the North Denmark and Central Denmark regions | Children with chromosomal abnormalities, and those born of women diagnosed with gestational diabetes in the index pregnancy | No | Pregnancy | Pregnancy |
| Endo, 2018 | Women with singleton pregnancies who received perinatal care at a hospital | Twins and steroid-induced diabetes | No | Preconception, pregnancy | Pregnancy |
| Kekki, 2021 | Pregnant women at gestational age 35+0 to 42.6, singleton live birth, born vaginally with cephalic presentation | Delivery by forceps, major congenital anomalies | No | Pregnancy | Pregnancy |
| Lin, 2020 | Women aged 20-44 years with pre-existing T2D and singleton pregnancies at ≥20 weeks gestation | Diagnosis of T1D | Yes | Preconception, pregnancy | Pregnancy |
| Kallas-Koeman,2012 | Women referred to Diabetes in Pregnancy programme | Women with impaired glucose tolerance, gestational diabetes, partial records referred prior to 2006 | No | Preconception | Pregnancy |
| Murphy, 2021 | Women with pre-existing diabetes who completed a pregnancy during the study period and who were included in the annual National pregnancy in Diabetes audit of 172 maternity clinics across England, Wales, and the Isle of Man | Women with monogenic diabetes and those who presented with diabetes first recognised during pregnancy | No | Preconception, pregnancy | Pregnancy, postnatal |
| Oppermann, 2020 | Women delivering at or above 23 weeks gestation | Women with fewer than two antenatal visits, multiple pregnancies, uncertain diagnosis of diabetes, or missing information on pre-eclampsia | No | Preconception, pregnancy | Pregnancy |
| Rais, 2019 | Women who received prenatal care at the Women and Infants Hospital of Rhode Island (WIHRI) | None reported. | No | Pregnancy | Pregnancy, postnatal |
| Rowan, 2009 | Women with pre-existing T2D and an ongoing pregnancy beyond the first trimester, singleton pregnancies | Twins and a miscarriage. | Yes | Pregnancy | Pregnancy |
| Rowe, 2021 | Women with pre-existing T1D pr T2D who received antenatal betamethasone | None reported. | No | Pregnancy | Pregnancy, postpartum |
| Søholm, 2021 | Women <20 weeks gestation with pre-existing diabetes and consecutive singleton pregnancies | Younger than 18 years, concomitant condition, insufficient Danish language skills, recurrent pregnancy during study period | No | Pregnancy | Pregnancy |
| Yee, 2011 | Women with overweight or obesity with T2D enrolled in the California Diabetes and Pregnancy Program (CDAPP) | Other forms of diabetes, multiple pregnancies or those with fetal anomalies | Yes | Pregnancy | Pregnancy, postpartum |
| Soepnel, 2019 | Women with T1D, T2D or gestational diabetes | Those who presented later than 36/40 or received <2/52 of medical intervention (when analysing the role of oral hypoglycaemic agents) | No | Pregnancy | Pregnancy |
| Asbjornsdottir, 2021 | Women with pre-existing T2D and singleton pregnancy before 20 weeks gestation | Insufficient Danish language skills | Yes | Pregnancy | Pregnancy |
| Asbjornsdottir, 2013 | Women with pre-existing T2D treated with diet or oral meds, referred <22 weeks gestation, singleton pregnancy, BMI ≥ 30 kg/m^2^, no concurrent illness that could affect weight gain | Women with BMI <30 kg/m^2^, former bariatric surgery, incomplete data on pregnancy weight gain or other medial issues | Yes | Pregnancy | Pregnancy |
| Feig, 2022 | Women with T2D diagnosed prior to pregnancy or in the first 20 weeks, 18-45 years of age, using insulin, and live singleton fetus between 6 and 22 weeks, 6 days gestation; gestational age known | Women with T1D, known intolerance or contraindications to metformin, previously participated in MiTy, serum creatinine >130 mmol/L or creatinine clearance <60 mL/min. | Yes | Pregnancy | Pregnancy |
| Kapustin, 2023 | Women with singleton pregnancies affected by T1D, T2D, gestational diabetes | Multiple pregnancies; chromosomal abnormalities | No | Pregnancy | Pregnancy |
| Kapustin, 2022 | Women with T2D on diet or insulin therapy, singleton pregnancy | Women with diseases associated with symptomatic diabetes: thyrotoxicosis, hyperadrenocorticism, pituitary adenoma, and pheochromocytoma; severe comorbidities; malignancy; multiple pregnancies; patient's refusal to participate in the study | No | Pregnancy | Pregnancy |
| Kjerpeseth, 2023 | Women with singleton live-born or stillborn infants with prenatal exposure to insulin or metformin in first trimester | Women with other indications, such as polycystic ovary syndrome and assisted reproductive treatment; terminations; infants diagnosed with a teratogenic infection, chromosomal anomaly, microdeletion, or genetic syndrome within 1 year of birth; infants with potential exposure to other glucose-lowering drugs or known teratogenic drugs in the first trimester; mother on insulin between 90 days before last menstrual period and end of first trimester and recorded diagnosis of T1D | Yes | Pregnancy | Pregnancy |
| Longmore, 2022 | Women with hyperglycaemia in pregnancy | (1) intrauterine fetal death, stillbirth, neonatal or infant death prior to 15 months; (2) born to mothers with T1D; (3) multiple pregnancy; (4) siblings of previous participant; (5) born at a gestation of 34 weeks or less; (6) born to mothers who declined follow-up after birth or withdrew from the study prior to 12 months post-partum; (7) infants who were not Aboriginal or Torres Strait Islander | No | Postpartum | Postpartum |
| Ahmed, 2022 | Women with T2D, ≥20 weeks gestation | Women with gestational diabetes; <20 weeks pregnancy | Yes | Pregnancy | Pregnancy |
| Alrais, 2022 | Women with insulin treated T2D who had non-anomalous singleton pregnancy | Women with chronic renal failure, proliferative retinopathy, or major congenital anomalies | Yes | Pregnancy | pregnancy |
| Cordero, 2022 | Women with T2D with obesity who intended to breastfeed during pregnancy | None reported. | No | Pregnancy | Postpartum |
| Stafl, 2023 | Women with pre-existing T2D | Women who delivered outside of the study area, had an unclear definition of type of diabetes, an unknown expected date of confinement, or delivered after the study period | No | Pregnancy | Pregnancy |
| Sushko, 2023 | Women with T1D and T2D, data collected in each trimester | Too medically complex | No | Pregnancy | Pregnancy |
| Cesta, 2023 | Pregnant women with T2D | Perinatal deaths, diagnosis of a fetal chromosomal abnormality or known exposure to teratogenic medicine, T1D, gestational diabetes | Yes | Pregnancy | Postpartum |
| Nørgaard, 2016 | Women with singleton pregnancies, T1D or T2D, giving birth after 22 weeks | Women with previous bariatric surgery, severe concomitant illness, without information on preconception BMI | No | Preconception | Pregnancy |
| McLean, 2023 | Women 18 years or older referred to the diabetes service with pre-existing T2D in pregnancy before 30 weeks gestation | Women with T1D, gestational diabetes, or a concomitant medical condition that could prevent study completion, stillbirth, birthed at another institution for medical reasons | Yes | Pregnancy | Pregnancy |
| Olmos, 2009 | NR | NR | No | Preconception, pregnancy | Pregnancy |
| Feghali, 2017 | Women with T2D diagnosed prior to pregnancy; singleton pregnancies | None reported. | Yes | Pregnancy | Pregnancy |
| Racine, 2021 | Women >18 years with singleton pregnancies complicated with T2D (preconception or first trimester) | <18 years old, multifetal gestations, women with T1D or gestational diabetes, and women who did not deliver at the institution | Yes | Pregnancy | Pregnancy |
| Bartal, 2019 | Women with pre-existing T2D requiring medical treatment or overt diabetes diagnosed prior to 20 weeks gestation | T1D and gestational diabetes, pregnancy loss, multifetal gestation, major anomalies, chronic renal failure), proliferative retinopathy | Yes | Pregnancy | Pregnancy |
| Wong, 2013 | Women with pre-existing diabetes attending a specialist antenatal diabetes clinic with singleton pregnancies | Women with gestational diabetes | No | Preconception | Pregnancy, postpartum |
| Egan, 2016 | Women with T1D and T2D referred to the pre-pregnancy care (PPC) programme | None reported | No | Preconception | Pregnancy, postpartum |
| Yamamoto, 2018 | Women with diabetes (T2D and other, including MODY who participated in a pre-pregnancy care (PPC) programme through their primary care practice or NHS specialist antenatal diabetes clinic | T1D and diabetes diagnosed during pregnancy | No | Preconception | Preconception, pregnancy |
| Murphy, 2010 | Women with T1D and T2D | Pregnancies from women who moved out of the area, pregnancies in women with no diabetes data | No | Preconception | Preconception, pregnancy, postpartum |
| Ásbjörnsdóttir, 2019 | Women ≥18 years with singleton pregnancy before 20 weeks gestation and with T2D at referral or in early pregnancy | Previous bariatric surgery, insufficient Danish language skills | Yes | Pregnancy | Pregnancy, postpartum |
| Do, 2021 | Pregnant women with pre-existing diabetes and a single living fetus <20 weeks gestation referred to Center for Pregnant Women with Diabetes | Ages <18 years, insufficient Danish language skills, participation in the study in a previous pregnancy, severe concomitant diseases | No | Pregnancy | Pregnancy, postpartum |
| Perichart-Perera, 2009 | Women with pre-existing T2D and gestational diabetes; gestational age ≤29 weeks | Gestational diabetes subclass A1; T1D; renal, hepatic, or thyroid disorders; diabetes complications | No | Pregnancy | Pregnancy |
| Nadeau, 2021 | Women with a singleton gestation who reported a diagnosis of T2D at their first prenatal visit; delivered after 20 weeks’ gestation | Women who did not require insulin therapy, elected to continue oral agents during pregnancy, and did not have a documented insulin dose at delivery | Yes | Pregnancy | Pregnancy, postpartum |

Abbreviations: BMI (Body Mass Index); GP (General Practitioner); NR (Not Reported); PPC (Pre-pregnancy Care); T1D (Type 1 diabetes).

^a^ Preconception, pregnancy and/or postpartum period.

**Supplementary Table 3**. Newcastle - Ottawa Scale (NOS) quality assessment guide

| **Bias assessment** | **Question (Q)** | **Response options** |
| --- | --- | --- |
| **Selection** (note: the population is only women with type 2 diabetes and the research will include multiple exposures) | 1) Representativeness of the exposed cohort (i.e. the list of exposures of interest in exposures/outcomes tab) – select one only | a) truly representative of the average maternal preconception/pregnancy/postpartum T2D population in the community (e.g. they recruited all women in a set time period) *  b) somewhat representative of the average maternal preconception/pregnancy/postpartum T2D population in the community (e.g. they compared the population they recruited with the local population and they have similar characteristics) *  c) Selected group of users e.g. over 35 years, restricted to one ethnic group, Poly Cystic Ovary Syndrome etc  d) no description of the derivation of the cohort |
|  | 2) Selection of the non-exposed cohort (i.e. the list of exposures of interest in exposures/outcomes tab) – select one only | a) drawn from the same community as the exposed cohort (e.g. recruited women from the same unit at the same time) *  b) drawn from a different source (e.g. recruited women from the same unit but at a different time, or from a different unit)  c) no description of the derivation of the non-exposed cohort |
|  | 3) Ascertainment of exposure – select one only | a) secure record (e.g. explicitly measured BMI, HbA1c, blood test etc) *  b) structured interview (e.g. self-report via validated questionnaire (e.g. diet 24 hour recall), validated self-report of smoking behaviour with carbon monoxide testing (but used the self-report as the exposure)) *  c) any self-report (e.g. unvalidated self-report of folic acid use, smoking, medication adherence etc)  d) no description |
| **Comparability** | 4) Comparability of cohorts on the basis of the design or analysis - a and b can both be selected | a) study controls for glycaemia (unless glycaemia is the exposure, then BMI would be the priority factor to control for) *  b) study controls for any additional factor *  c) no factors controlled for |
| **Outcome** | 5) Assessment of outcome – select one only | a) independent blind assessment (e.g. specifically measured for the research) *  b) record linkage (e.g. outcomes from routine medical records) *  c) self-report  d) no description |
|  | 6) Was follow-up long enough for outcomes to occur – select one only | a) yes (select an adequate follow up period for outcome of interest: followed up for long enough for the outcome to develop, e.g. all women followed up until delivery for preterm birth outcome etc) *  b) no |
|  | 7) Adequacy of follow up of cohorts – select one only | a) complete follow up - all subjects accounted for (i.e. no loss to follow up for prospective cohorts) *  b) subjects lost to follow up unlikely to introduce bias - small number lost (>80% follow up for prospective cohorts, or for retrospective cohorts >80% with the data required for analysis) or description provided of those lost) *  c) follow up rate < 80% or >20% excluded due to missing data for the analysis and no description of those lost  d) no statement |

Abbreviations: BMI (Body Mass Index); HBA1c (haemoglobin A1c, a blood test that measures the average blood sugar level over the past two to three months).

**Supplementary table 4**. Quality assessment results using the Newcastle - Ottawa Scale (NOS)

| **First author, year** | **Newcastle Ottawa Scale question number and score allocated ^a^** | | | | | | | | |
| --- | --- | --- | --- | --- | --- | --- | --- | --- | --- |
|  | **Q1** | **Q2** | **Q3** | **Q4** | **Q5** | **Q6** | **Q7** | **Total stars awarded^b^** | **Rating ^c^** |
| Colatrella*,* 2009 | b* | a* | a* | c | b* | a* | a* | 6 | High |
| Cyganek, 2011 | b* | a* | a* | c | b* | a* | d | 5 | Medium |
| Murphy, 2017 | a* | a* | a* | c | b* | a* | a* | 6 | High |
| Morikawa, 2020 | a* | a* | a* | c | b* | a* | a* | 6 | High |
| Rasmussen, 2010 | a* | a* | a* | c | b* | a* | a* | 6 | High |
| Roland, 2005 | a* | a* | a* | a*/b* | b* | a* | a* | 8 | High |
| Owens, 2015 | a* | a* | a* | c | b* | a* | a* | 6 | High |
| Yamamoto, 2020 | a* | a* | a* | b* | b* | a* | a* | 7 | High |
| Parellada, 2014 | a* | b | a* | a*/b* | b* | a* | c | 7 | High |
| Shannon, 2016 | b* | a* | a* | c | b* | a* | b* | 6 | High |
| Gaudio 2020 | a* | a* | a* | c | b* | a* | a* | 6 | High |
| Isabey, 2021 | b* | a* | a* | c | b* | a* | b* | 6 | High |
| Persson, 2016 | a* | a* | a* | b* | a* | a* | a* | 7 | High |
| Ladfors, 2017 | a* | a* | b* | b* | b* | a* | c | 6 | High |
| Maple-Brown, 2019 | a* | a* | a* | b* | b* | a* | a* | 7 | High |
| Alexander, 2019 | a* | a* | a* | b* | b* | a* | a* | 7 | High |
| Abell, 2017 | a* | a* | a* | a*/b* | b* | a* | b* | 8 | High |
| Allen, 2018 | a* | a* | b* | b* | b* | a* | d | 7 | High |
| Arendt, 2021 | a* | a* | a* | c | b* | a* | b* | 6 | High |
| Endo, 2018 | d | a* | b* | c | b* | a* | b* | 5 | Medium |
| Kekki, 2021 | a* | a* | d | c | b* | a* | b* | 5 | Medium |
| Lin, 2020 | a* | a* | a* | b* | b* | a* | d | 6 | High |
| Kallas-Koeman, 2012 | a* | a* | b* | c | b* | a* | c | 5 | Medium |
| Murphy, 2021 | a* | a* | a* | a*/b* | b* | a* | d | 7 | High |
| Oppermann, 2020 | d | a* | a* | c | b* | a* | b* | 5 | Medium |
| Rais, 2019 | a* | a* | a* | c | b* | a* | a* | 6 | High |
| Rowan, 2009 | a* | a* | a* | c | b* | a* | b* | 6 | High |
| Rowe, 2021 | a* | a* | a* | c | b* | a* | a* | 6 | High |
| Søholm, 2021 | a* | a* | a* | c | b* | a* | b* | 6 | High |
| Yee, 2011 | a* | a* | a* | b* | b* | a* | d | 6 | High |
| Soepnel, 2019 | a* | a* | b* | c | b* | a* | b* | 6 | High |
| Asbjornsdottir, 2021 | a* | a* | a* | c | b* | a* | b* | 6 | High |
| Asbjornsdottir, 2013 | a* | a* | a* | b* | b* | a* | a* | 7 | High |
| Feig, 2022 | a* | a* | a* | c | b* | a* | a* | 7 | High |
| Kapustin, 2023 | a* | a* | a* | c | b* | a* | a* | 6 | High |
| Kapustin, 2022 | a* | a* | a* | c | b* | a* | a* | 6 | High |
| Kjerpeseth, 2023 | a* | a* | a* | b* | b* | a* | a* | 7 | High |
| Longmore, 2022 | a* | a* | b* | b* | b* | a* | b* | 6 | High |
| Ahmed, 2022 | a* | a* | a* | c | b* | a* | a* | 6 | High |
| Alrais, 2022 | a* | a* | a* | b* | b* | a* | a* | 7 | High |
| Cordero, 2022 | a* | a* | a* | b* | b* | a* | a* | 7 | High |
| Stafl, 2023 | a* | a* | a* | b* | b* | a* | a* | 7 | High |
| Sushko, 2023 | a* | a* | b* | b* | c | a* | a* | 6 | High |
| Cesta, 2023 | a* | a* | a* | b* | b* | a* | a* | 7 | High |
| Nørgaard, 2016 | a* | a* | c | c | b* | a* | a* | 5 | Medium |
| McLean, 2023 | a* | a* | a* | b* | a* | a* | a* | 7 | High |
| Olmos, 2009 | a* | a* | a* | c | b* | a* | a* | 6 | High |
| Feghali, 2017 | a* | a* | a* | b* | b* | a* | a* | 7 | High |
| Racine, 2021 | a* | a* | a* | b* | b* | a* | a* | 7 | High |
| Bartal, 2019 | a* | a* | a* | b* | b* | a* | a* | 7 | High |
| Wong, 2013 | a* | a* | a* | c | b* | a* | a* | 6 | High |
| Egan, 2016 | a* | a* | a* | c | a* | a* | a* | 6 | High |
| Yamamoto, 2018 | b* | a* | a* | c | b* | a* | b* | 6 | High |
| Murphy, 2010 | a* | a* | a* | a*/b* | b* | a* | b* | 8 | High |
| Ásbjörnsdóttir, 2019 | a* | a* | a* | a*/b* | b* | a* | b* | 8 | High |
| Do, 2021 | a* | a* | a* | b* | b* | a* | b* | 7 | High |
| Perichart-Perera, 2009 | a* | b | a* | b* | b* | a* | b* | 7 | High |
| Nadeau, 2021 | a* | a* | a* | b* | b* | a* | b* | 7 | High |
| **Total** | **n*=56**  **96.6%** | **n*= 56**  **96.6%** | **n*= 56**  **96.6%** | **n*= 29**  **50.0%** | **n*= 57**  **98.3%** | **n*= 58**  **100%** | **n*= 50**  **86.2%** | **Range 5-8** | **Medium n=6 (10.3%)**  **High n=52 (89.7%)** |

^a^ Newcastle-Ottawa question numbers 1-7, answers and associated number of stars (*) are detailed in Supplementary Table 18. ^b^ Minimum number of possible stars to be awarded = 0, maximum number of possible stars to be awarded = 8. ^c^ Categories were allocated as: Low = 0-2 stars, Medium = 3-5 stars, High = 6-8 stars

**Supplementary Table 5**. Findings on associations between metformin and maternal and child health outcomes

| **First author, year** | **Exposure description** | **Pregnancy outcome** | **Total sample size** | **Result** | **Confounder adjustments** |
| --- | --- | --- | --- | --- | --- |
| ***Outcome - NICU/SCBU admission*** | | | | | |
| Maple-Brown. 2019 | Metformin | SCBU admission | 38 non- indigenous women | OR (95% CI) 0.66 (0.14, 3.14), p=0.6 | Maternal age, body mass index and using insulin medication in the third trimester |
| Maple-Brown, 2019 | Metformin | SCBU admission | 234 indigenous women | OR (95% CI) 0.77 (0.26, 2.25), p=0.63 | Maternal age, body mass index and using insulin medication in the third trimester |
| Racine, 2021 | Metformin | NICU Admission | 254 | aOR (95% CI) 1.01 (0.56-1.82), p=0.97 | Pre-pregnancy BMI, chronic HTN, and history of prior hypertensive disorders of pregnancy |
| ***Outcome - Caesarean section*** | | | | | |
| Maple-Brown, 2019 | Metformin | Caesarean section | 38 non- indigenous women | OR (95% CI) 0.53 (0.1, 3.3), p=0.51 | Maternal age, body mass index and using insulin medication in the third trimester |
| Maple-Brown, 2019 | Metformin | Caesarean section | 234 indigenous women | OR (95% CI) 0.64 (0.20, 2.1), p= 0.46 | Maternal age, body mass index and using insulin medication in the third trimester |
| Lin, 2020 | Metformin vs Insulin | Primary caesarean section | 596 | OR (95% CI) 0.57 (0.40–0.82), p=NR | Age, duration of T2D, hypertension, hyperlipidaemia, retinopathy, and aspirin use |
| Lin, 2020 | Metformin vs Insulin among those with T2D, < 3 years duration | Primary caesarean section | 427 | OR (95% CI) 0.50 (0.30–0.83, p=NR | Age, duration of T2D, hypertension, hyperlipidaemia, retinopathy, and aspirin use |
| ***Outcome - Hypertension*** | | | | | |
| Racine, 2021 | Metformin | *Hypertension Composite | 254 | aOR (95% CI) 0.53 (0.29-0.96), p=0.4 | Pre-pregnancy BMI, chronic HTN, and history of prior hypertensive disorders of pregnancy |
| Racine, 2021 | Metformin | *Hypertension Composite | 180 | aOR (95% CI) 0.64 (0.32-1.27), p=0.2 | Pre-pregnancy BMI, and history of prior hypertensive disorders of pregnancy, i.e without chronic HTN |
| Racine, 2021 | Metformin | Gestational hypertension | 254 | aOR (95% CI) 1.65 (0.59-5.14), p=0.39 | Adjusted for pre-pregnancy BMI, chronic HTN, and history of prior hypertensive disorders of pregnancy |
| Racine, 2021 | Metformin | Preeclampsia with severe features | 42 | aOR (95% CI) 0.38 (0.18-0.81), p=0.01 | As above |
| Lin, 2020 | Metformin vs Insulin | Pregnancy related hypertension | 626 | aOR (95%CI) 0.92 (0.61–1.40), p=NR | Age, duration of T2D, hypertension, hyperlipidaemia, retinopathy, and aspirin use |
| Lin, 2020 | T2D, <3 years duration - metformin | Pregnancy related hypertension | 427 | aOR (95% CI) 1.08 (0.55–2.11) | As above |
| ***Outcome - Delivery <32 weeks*** | | | | | |
| Lin, 2020 | Metformin vs Insulin | Very preterm delivery (< 32 weeks) | 626 | aOR (95%CI) 1.01 (0.42–2.46), p=NR | Age, duration of T2D, hypertension, hyperlipidaemia, retinopathy, and aspirin use |
| ***Outcome - Delivery <34 weeks*** | | | | | |
| Racine, 2021 | Metformin | Preterm delivery (<34 weeks | 254 | aOR (95% CI)1.47 (0.50-4.27) | Adjusted for pre-pregnancy BMI, chronic HTN, and history of prior hypertensive disorders of pregnancy |
| ***Outcome - Delivery <37 weeks*** | | | | | |
| Maple-Brown, 2019 | Metformin | Delivery <37 weeks | 38 non- indigenous women | OR (95% CI) 0.87 (0.08, 9.15), p= 0.9 | Maternal age, body mass index and using insulin medication in the third trimester |
| Maple-Brown, 2019 | Metformin | Delivery <37 weeks | 234 indigenous women | OR (95% CI) 0.71 (0.27, 1.88), p=0.49 | As above |
| Lin, 2020 | Metformin vs insulin | Delivery <37 weeks | 626 | OR (95% CI) 1.05 (0.69–1.59), p=nr | Age, duration of T2D, hypertension, hyperlipidaemia, retinopathy, and aspirin use |
| Lin, 2020 | T2D, <3 years duration – metformin vs insulin | Delivery <37 weeks | 427 | OR (95% CI) 0.79 (0.43–1.47), p=NR | Age, duration of T2D, hypertension, hyperlipidaemia, retinopathy, and aspirin use |
| Racine, 2021 | Metformin | Delivery <37 weeks | 254 | OR (95% CI) 1.05 (0.56-1.97), p=0.88 | Adjusted for pre-pregnancy BMI, chronic HTN, and history of prior hypertensive disorders of pregnancy |
| ***Outcome - Pre-eclampsia*** | | | | | |
| Lin, 2020 | Metformin vs insulin | Preeclampsia | 626 | aOR (95% CI) 0.92 (0.57-1.49), p=NR | Age, duration of T2D, hypertension, hyperlipidaemia, retinopathy, and aspirin use |
| Lin, 2020 | T2D, <3 years duration – metformin vs insulin | Preeclampsia | 427 | aOR (95% CI) 0.97 (0.44-2.14), p=NR | Age, duration of T2D, hypertension, hyperlipidaemia, retinopathy, and aspirin use |
| Racine, 2021 | Metformin | Preeclampsia with severe features | 254 | aOR (95% CI) 0.38 (0.18-0.81), p=0.01 | Adjusted for pre-pregnancy BMI, chronic HTN, and history of prior hypertensive disorders of pregnancy |
| Racine, 2021 | Metformin | Preeclampsia with severe features | 180 | aOR (95% CI) 0.35 (0.13-0.94), p=0.04 | Pre-pregnancy BMI, and history of prior hypertensive disorders of pregnancy, ie without chronic HTN |
| Racine, 2021 | Metformin | Preeclampsia without severe features | 254 | aOR (95% CI 0.89 (0.28-2.77), p=0.84 | Pre-pregnancy BMI, chronic HTN, and history of prior hypertensive disorders of pregnancy |
| Racine, 2021 | Metformin | Preeclampsia without severe features | 180 | aOR (95% CI 1.35 (0.38-4.79), p=0.64 | Pre-pregnancy BMI, and history of prior hypertensive disorders of pregnancy, ie without chronic HTN |
| ***Outcome - SGA*** | | | | | |
| Maple-Brown, 2019 | Metformin | SGA | 38 non- indigenous | ORs (95% CI) 0.0232 (0.0011 to 0.4751), p=0.0146 | Maternal age, body mass index and using insulin medication in the third trimester |
| Maple-Brown, 2019 | Metformin | SGA | 234 indigenous women | ORs (95% CI) 1.6000 (0.2003 to 12.7778), p=0.6575 | As above |
| Feig, 2022 | Metformin | SGA | 460 | ORs (95% CI) 2.1089 (1.1020 to 4.0357), p=0.0242 |  |
| Lin, 2020 | Metformin vs insulin | SGA | 626 | aORs (95% CI) 0.98 (0.51–1.90), p=NR | Age, duration of T2D, hypertension, hyperlipidaemia, retinopathy, and aspirin use |
| Rowan, 2009 | Metformin | SGA | 159 | ORs (95% CI) 0.7855 (0.3539 to 1.7431), p= 0.5527 |  |
| ***Outcome - Low birth weight <2500g*** | | | | | |
| Lin, 2020 | Metformin vs insulin | LBW (<2500g) | 626 | ORs (95% CI) 1.30 (0.75–2.25) |  |
| Lin, 2020 | T2D, <3 years duration – metformin | LBW (<2500g) | 427 | ORs (95% CI) 1.27 (0.52–3.09) |  |
| ***Outcome - IUGR*** | | | | | |
| Racine, 2021 | Metformin | Restricted fetal growth | 254 | aOR (95% CI) 4.11 (0.84-20.2) | Pre-pregnancy BMI, chronic HTN, and history of prior hypertensive disorders of pregnancy |
| ***Outcome - LGA*** | | | | | |
| Maple-Brown, 2019 | Metformin | LGA | 38 non- indigenous women | ORs (95% CI) 0.6667 (0.1281 to 3.4699), p= 0.63 | Maternal age, body mass index and using insulin medication in the third trimester |
| Maple-Brown, 2019 | Metformin | LGA | 234 indigenous women | ORs (95% CI) 0.7177 (0.3102 to 1.6606), p= 0.4383 | Maternal age, body mass index and using insulin medication in the third trimester |
| Lin, 2020 | Metformin vs insulin | LGA | 626 | ORs (95% CI) 0.99 (0.69–1.42), p=NR | Age, duration of T2D,, hypertension, hyperlipidaemia, retinopathy, and aspirin use |
| Lin, 2020 | T2D, <3 years duration – metformin | LGA | 427 | ORs (95% CI) 0.79 (0.47–1.33). p=NR | As above |
| Rowan, 2009 | Metformin | LGA | 180 | ORs (95% CI) 0.6182 (0.3147 to 1.2144), p=0.1627 |  |
| ***Outcome - High birth weight*** | | | | | |
| Lin, 2020 | Metformin vs insulin | High birth weight (> 4000 g) | 626 | aOR (95% CI) 0.91 (0.54–1.53) | Age, hypertension, hyperlipidaemia, retinopathy, and aspirin use |
| ***Outcome - Adverse fetal outcomes*** | | | | | |
| Kjerpeseth, 2023 | Metformin monotherapy | Any major congenital malformations | 3730 | OR (95% CI) 0.77 (0.55 to 1.08), p= 0.131 |  |
| Kjerpeseth, 2023 | Metformin and insulin | Any major congenital malformations | 1171 | OR (95% CI) 1.21 (0.71 to 2.07), p= 0.48 |  |
| Lin, 2020 | Metformin vs insulin | Congenital malformations | 626 | aOR (95% CI) 0.51 (0.27–0.94), p=0.032 | Age, duration of T2D, hypertension, hyperlipidaemia, retinopathy, and aspirin use |
| Lin, 2020 | T2D, <3 years duration – metformin | Congenital malformations | 427 | aOR (95% CI) 0.40 (0.17–0.93), p=0.033 | As above |
| Kjerpeseth, 2023 | Metformin monotherapy | Major cardiac malformations | 3730 | OR (95% CI) 0.89 (0.52 to 1.53), p=0.665 |  |
| Kjerpeseth 2023 | Metformin and insulin | Major cardiac malformations | 1171 | OR (95% CI) 1.86 (0.87 to 3.99), p= 0.11 |  |
| Racine, 2021 | Metformin | Respiratory Distress Syndrome | 254 | OR (95% CI) 1.17 (0.38-3.6), p= 0.82 | Pre-pregnancy BMI, chronic HTN, and history of prior hypertensive disorders of pregnancy |
| Lin, 2020 | Metformin vs insulin | Stillbirth | 626 | aOR (95% CI) 0.82 (0.28–2.36), p=NR | Age, duration of T2D, hypertension, hyperlipidaemia, retinopathy, and aspirin use |
| Lin, 2020 | Type 2 diabetes <3 years duration – metformin | Stillbirth | 427 | aOR (95% CI) 0.23 (0.06–0.92), p=NR | As above |
| Racine, 2021 | Metformin | Fetal/neonatal death | 254 | aOR (95% CI) 0.65 (0.03-11.8), p= 0.78 | Pre-pregnancy BMI, chronic HTN, and history of prior hypertensive disorders of pregnancy |
| Racine, 2021 | Metformin | Hypoglycaemia | 254 | aOR (95% CI) 1.38 (0.67-2.83), p=0.37 | As above |
| Lin, 2020 | Metformin vs insulin | Apgar score <7 at 5 minutes | 626 | aOR (95% CI) 1.55 (0.31–7.67), p=NR | Age, duration of T2D,, hypertension, hyperlipidaemia, retinopathy, and aspirin use |
| Racine, 2021 | Metformin | Apgar score <7 at 5 minutes | 254 | OR (95% CI) 0.89 (0.32-2.51), p=0.83 | Pre-pregnancy BMI, chronic HTN, and history of prior hypertensive disorders of pregnancy |
| Soepnel, 2019 | Therapy type - Metformin vs insulin/metformin/glibenclamide | Maternal weight gain over pregnancy | 71 | Median (IQR) Exposed group (metformin monotherapy) 3.5 (0.45 - 6.4) vs non-exposed group 7.1 (2.5 - 11), p=0.008 |  |
| Maple-Brown, 2019 | Metformin | Birth weight z score | 38 non- indigenous women | β (95% CI) 1.2 (0.1, 2.3), p=0.037 | Maternal age, body mass index and using insulin medication in the third trimester, and parity |
| Maple-Brown, 2019 | Metformin | Birth weight z score | 234 indigenous women | β (95% CI) −0.5 (−1.2, 0.2), p=0.15 | Maternal age, body mass index and using insulin medication in the third trimester, and parity |
| Soepnel, 2019 | Therapy type - Metformin vs insulin/metformin/glibenclamide | Mean BGL <7.1 in third trimester | 63 | OR (95% CI) 0.41 (0.04 to 3.90), p=P = 0.4371 |  |

Abbreviations: aOR (Adjusted Odds Ratio); APGAR (refers to the scoring system used by medical professionals to assess the health of a newborn baby shortly after birth and stands for Appearance, Pulse, Grimace, Activity, and Respiration); BGL (Blood Glucose Level); CI (Confidence Interval); BMI (Body Mass Index); HTN (hypertension); IQR (inter-quartile range); IUGR (intrauterine growth restriction); LGA (large for gestational age); NICU (Neonatal Intensive Care Unit); OR (Odds Ratio); SCBU (Special Care Baby Unit); SGA (small for gestational age); T2D (type 2 diabetes).

*Hypertension Composite: gestational hypertension, preeclampsia with or without severe features, Hemolysis, Elevated Liver enzymes and Low Platelets (HELLP) syndrome, or eclampsia.

**Supplementary Table 6**. Findings on associations between insulin and maternal and child health outcomes

| **First author, year** | **Exposure description** | **Pregnancy outcome** | **Total sample size** | **Result** | **Confounder adjustments** |
| --- | --- | --- | --- | --- | --- |
| ***Outcome - APGAR score*** | | | | | |
| Bartal, 2019 | Basal insulin NPH vs. glargine or detemir - (no disaggregation of analogs) | 5 minutes APGAR score <5 | 233 (n=119, NPH; n=114, analog) | aRR (95% CI)3.24 (0.43-24.53), p=0.08 | Age, race, ethnicity, diabetes medications pre-pregnancy, metformin and year of delivery |
| ***Outcome - Caesarean delivery*** | | | | | |
| Nadeau, 2021 | Insulin dose ≥2 units/kg at delivery | Caesarean delivery | 160 | OR (95% CI) 1.5200 (0.7963 to 2.9016), p=0.2043 |  |
| Bartal, 2019 | Basal insulin NPH vs. glargine or detemir - (no disaggregation of analogs) | Caesarean delivery | 233 (n=119, NPH; n=114, analog) | aRR (95% CI) 0.93 (0.75-1.15), p=0.3 | Age, race, ethnicity, diabetes medications pre-pregnancy, metformin and year of delivery |
| Lin, 2020 | Switching from pre-pregnancy metformin to insulin in pregnancy <12 weeks' gestation | Caesarean delivery | 318 | aOR (95% CI) 1.14 (0.80–1.63), p=NR | Age, duration of type 2 diabetes, hypertension, hyperlipidaemia, retinopathy, and aspirin use |
| Lin, 2020 | Type 2 diabetes <3 years duration - switching from pre-pregnancy metformin to insulin in pregnancy <12 weeks' gestation | Caesarean delivery | 154 | aOR (95% CI) 1.01: 0.59–1.74), p=NR | Age, duration of type 2 diabetes, hypertension, hyperlipidaemia, retinopathy, and aspirin use |
| Bartal, 2019 | Basal insulin NPH vs. glargine or detemir - (no disaggregation of analogs) | Caesarean delivery | 233 (n=119, NPH; n=114, analog) | aRR (95% CI) 0.44: 0.25-0.78), p=0.01 | Age, race, ethnicity, diabetes medications pre-pregnancy, metformin and year of delivery |
| ***Outcome - Congenital anomaly*** | | | | | |
| Nadeau, 2021 | Insulin dose ≥2 units/kg at delivery | Congenital Anomalies | 160 | OR (95% CI) 1.242 (0.3829 to 4.0316), p=0.7178 |  |
| Lin, 2020 | Switching from pre-pregnancy metformin to insulin in pregnancy <12 weeks gestation | Congenital Anomalies | 318 | aOR (95% CI) 0.75 (0.41–1.37), p=NR | Age, duration of type 2 diabetes, hypertension, hyperlipidaemia, retinopathy, and aspirin use |
| Lin, 2020 | Type 2 diabetes <3 years duration - switching from pre-pregnancy metformin to insulin in pregnancy <12 weeks' gestation | Congenital Anomalies | 154 | aOR (95% CI) 0.60 (0.26–1.37), p=NR | hypertension, hyperlipidaemia, retinopathy, and aspirin use |
| ***Outcome - Hyperbilirubinemia*** | | | | | |
| Bartal, 2019 | Basal insulin NPH vs. glargine or detemir - (no disaggregation of analogs) | Hyperbilirubinemia | 233 (n=119, NPH; n=114, analog) | aRR (95% CI) 0.59 (0.34-1.05), p=0.24 | Age, race, ethnicity, diabetes medications pre-pregnancy, metformin and year of delivery |
| ***Outcome - Hyperglycaemia*** | | | | | |
| Rowe, 2021 | Pregnancy regimen Intravenous Insulin following steroid treatment | % of time with BGL <3.8mmol/l - h | 40 | % (IQR) Exposed group 2 (1-2) vs Unexposed group 3 (3-4) p=0.09 |  |
| Rowe, 2021 | Pregnancy regimen Intravenous Insulin following steroid treatment | %>10 mmol/l | 40 | % (IQR) Exposed group 6 (0 - 12) vs Unexposed group 4 (4 - 12) p=0.44 |  |
| Nadeau, 2021 | Insulin dose ≥2 units/kg at delivery | Baseline HbA1c (%) | 160 | Mean (SD) Exposed group 8.4 (±2.0) vs Unexposed group 7.3 (±1.9), p=NR |  |
| Rowe, 2021 | Pregnancy regimen Intravenous Insulin following steroid treatment | BGL > 10 mmol/l for 90 mins | 40 | OR (95% CI) 1.75 (0.49-6.22), p=0.387 |  |
| Rowe, 2021 | Pregnancy regimen Intravenous Insulin following steroid treatment | BGL > 10 mmol/l for 90 mins -2 or more episodes | 40 | OR (95% CI) 1.29 (0.27 to 6.07), p=0.75 |  |
| Rowe, 2021 | Pregnancy regimen Intravenous Insulin following steroid treatment | Mean BGL | 40 | Exposed group 6.9 (6.1 - 7.2) vs Unexposed group 7.0 (6.5-7.3), p=0.51 |  |
| Rowe, 2021 | Pregnancy regimen Intravenous Insulin following steroid treatment | TIR - % BGL 3.8 - 7.0 mmol/L (study target) | 40 | %, IQR, Exposed group 69 (46 - 84) vs Unexposed group 58 (44 - 69), p= 0.24 |  |
| Rowe, 2021 | Pregnancy regimen Intravenous Insulin following steroid treatment | TIR - % BGL 4 - 7.8 mmol/L (JBDS target) | 40 | %, IQR, Exposed group 77 (66 - 88) vs Unexposed group 73 (62 - 84), p= 0.36 |  |
| ***Outcome - Hypertensive disorders*** | | | | | |
| Morikawa, 2020 | Insulin dose ≥80 units/day at delivery | Hypertensive disorder of pregnancy | 109 | OR (95% CI) 7.43 (2.21-25.0), 0.0016 |  |
| Nadeau, 2021 | Insulin dose ≥2 units/kg at delivery | Hypertensive disorders of pregnancy | 233 (n=119, NPH; n=114, analog) | OR (95% CI) 0.8377 (0.4265 to 1.6456), p= 0.6072 |  |
| Bartal, 2019 | Basal insulin NPH vs. glargine or detemir - (no disaggregation of analogs) | Preeclampsia | 233 (n=119, NPH; n=114, analog) | aOR (95% CI) 1.27 (0.38-4.27), p= 0.7 | Age, race, ethnicity, diabetes medications pre-pregnancy, metformin and year of delivery |
| Lin, 2020 | Type 2 diabetes switching from pre-pregnancy metformin to insulin in pregnancy <12 weeks' gestation | Preeclampsia | 318 | aOR (95% CI) 1.24 (0.75–2.05), p=NR | Age, duration of type 2 diabetes, hypertension, hyperlipidaemia, retinopathy, and aspirin use |
| Lin, 2020 | Type 2 diabetes <3 years duration - switching from pre-pregnancy metformin to insulin in pregnancy <12 weeks gestation | Preeclampsia | 154 | aOR (95% CI) 1.39 (0.67–2.88), p=NR | Age, duration of type 2 diabetes, hypertension, hyperlipidaemia, retinopathy, and aspirin use |
| Bartal, 2019 | Basal insulin NPH vs. glargine or detemir - (no disaggregation of analogs) | preeclampsia with severe features | 233 (n=119, NPH; n=114, analog) | aOR (95% CI) 0.93 (0.53-1.66), p=0.81 | Age, race, ethnicity, diabetes medications pre-pregnancy, metformin and year of delivery |
| Lin, 2020 | Type 2 diabetes switching from pre-pregnancy metformin to insulin in pregnancy <12 weeks gestation | Pregnancy-related hypertension | 318 | aOR (95% CI) 1.26 (0.83–1.92), p=NR | Age, duration of type 2 diabetes, hypertension, hyperlipidaemia, retinopathy, and aspirin use |
| Lin, 2020 | Type 2 diabetes <3 years duration - switching from pre-pregnancy metformin to insulin in pregnancy <12 weeks gestation | Pregnancy-related hypertension | 154 | aOR (95% CI) 1.65 (0.86–3.16), p=NR | Age, duration of type 2 diabetes, hypertension, hyperlipidaemia, retinopathy, and aspirin use |
| ***Outcome - Hypoglycemia*** | | | | | |
| Rowe, 2021 | Intravenous Insulin following steroid treatment -generic (adult IVI) versus pregnancy algorithm (pregnancy-IVI) | Any BGL<3.8 mmol/L on pregnancy IVI | 40 | OR (95% CI) 0.97 (0.22 to 4.32), p=0.97 |  |
| Rowe, 2021 | Intravenous Insulin following steroid treatment -generic (adult IVI) versus pregnancy algorithm (pregnancy-IVI) | Duration (hours) BGL<3.8mmol/l | 40 | IQR Exposed group 0.8 (0.5 - 1.0 vs Unexposed group 1.5 (1.0 - 2.0), p=0.08 |  |
| Bartal, 219 | Basal insulin NPH vs. glargine or detemir - (no disaggregation of analogs) | hypoglycaemia | 233 (n=119, NPH; n=114, analog) | aRR (95% CI) 0.72 (0.35-1.45), p=0.15 | Age, race, ethnicity, diabetes medications pre-pregnancy, metformin and year of delivery |
| Nadeau, 2021 | Insulin dose ≥2 units/kg at delivery | Hypoglycaemia | 160 | OR (95% CI) 0.96 (0.5132 to 1.7957), p=0.8983 |  |
| ***Outcome - LGA*** | | | | | |
| Bartal, 2019 | Basal insulin NPH vs. glargine or detemir - (no disaggregation of analogs) | LGA | 233 (n=119, NPH; n=114, analog) | Mean (SD) Exposed group 3186.1 (±751.9) vs Unexposed group 2993.9 (±800.6), p=0.06 |  |
| Lin, 2020 | Type 2 diabetes switching from pre-pregnancy metformin to insulin in pregnancy <12 weeks gestation | LGA | 318 | aOR (95% CI) 1.39 (0.96–2.02), p=NR | Age, duration of type 2 diabetes, hypertension, hyperlipidaemia, retinopathy, and aspirin use |
| Lin, 2020 | Type 2 diabetes <3 years duration - switching from pre-pregnancy metformin to insulin in pregnancy <12 weeks gestation | LGA | 154 | aOR (95% CI) 1.43 (0.83–2.49), p=NR | Age, duration of type 2 diabetes, hypertension, hyperlipidaemia, retinopathy, and aspirin use |
| Bartal. 2019 | Basal insulin NPH vs. glargine or detemir - (no disaggregation of analogs) | LGA | 233 (n=119, NPH; n=114, analog) | aOR (95% CI) 1.56 (0.89-2.73), p=0.7 | Age, race, ethnicity, diabetes medications pre-pregnancy, metformin and year of delivery |
| Nadeau, 2021 | Insulin dose ≥2 units/kg at delivery | LGA | 160 | OR (95% CI) 0.6731 (0.3430 to 1.3209), p=0.2498 |  |
| Lin, 2020 | Type 2 diabetes switching from pre-pregnancy metformin to insulin in pregnancy <12 weeks gestation | LGA (birth weight > 4000 g) | 318 | aOR (95% CI) 1.47 (0.92–2.36), p=NR | Age, duration of type 2 diabetes, hypertension, hyperlipidaemia, retinopathy, and aspirin use |
| Kapustin, 2022 | Insulin treated type 2 diabetes | LGA | 229 | OR (95% CI) 0.85 (0.42 to 1.72), p0.66 |  |
| ***Outcome - Neonatal hypoglycemia*** | | | | | |
| Rowe, 2021 | Intravenous Insulin following steroid treatment -generic (adult IVI) versus pregnancy algorithm (pregnancy-IVI) | Neonatal hypoglycaemia | 15 | Mean (SD) Exposed group 2.1 ± 0.9 vs Unexposed group 2.6 ± 0.8, p=0.28 |  |
| Rowe, 2021 | Intravenous Insulin (IVI) following steroid treatment -generic (adult IVI) versus pregnancy algorithm (pregnancy-IVI) | Neonatal hypoglycaemia (<2.5mmol/l in first 48hrs) | 15 | OR (95% CI) 2.22 (0.28 to 17.63), p=0.4499 |  |
| Bartal, 2019 | Basal insulin NPH vs. glargine or detemir - (no disaggregation of analogs) | Neonatal hypoglycaemia | 233 (n=119, NPH; n=114, analog) | aOR (95% CI) 1.30 (0.88-1.92) | Age, race, ethnicity, diabetes medications pre-pregnancy, metformin and year of delivery |
| ***Outcome - Neonatal morbidity*** | | | | | |
| Nadeau, 2021 | Insulin dose ≥2 units/kg at delivery | Neonatal morbidity |  | OR (95% CI) 1.2174 (0.6299 to 2.3527), p=0.5584 |  |
| ***Outcome - NICU admission*** | | | | | |
| Bartal, 2019 | Basal insulin NPH vs. glargine or detemir - (no disaggregation of analogs) | NICU admission | 233 (n=119, NPH; n=114, analog) | aOR (95% CI) 1.06 (0.77-1.46), p=0.18 | Age, race, ethnicity, diabetes medications pre-pregnancy, metformin and year of delivery |
| Nadeau, 2021 | Insulin dose ≥2 units/kg at delivery | NICU admission | 160 | OR (95% CI) 1.0213 (0.5409 to 1.9284), p=0.9483 |  |
| ***Outcome - Perinatal death*** | | | | | |
| Kapustin, 2022 | Insulin treated type 2 diabetes | Perinatal death | 229 | OR (95% CI) 1.49 (0.85 to 2.64), p=0.16 |  |
| Bartal, 2019 | Basal insulin NPH vs. glargine or detemir - (no disaggregation of analogs) | Perinatal death | 233 (n=119, NPH; n=114, analog) | aRR (95% CI) 1.8 (0.27-13.32), p=0.37 | Age, race, ethnicity, diabetes medications pre-pregnancy, metformin and year of delivery |
| Nadeau, 2021 | Insulin dose ≥2 units/kg at delivery | Perinatal death | 160 | OR (95% CI) 0.6056 (0.0538 to 6.8176), p=0.6847 |  |
| ***Outcome - Preterm birth*** | | | | | |
| Bartal, 2019 | Basal insulin NPH vs. glargine or detemir - (no disaggregation of analogs) | Preterm birth (gestational age) | 233 (n=119, NPH; n=114, analog) | Mean (SD) Exposed group 36.2 (±2.1) vs Unexposed group 36.1 (±2.5), p=0.75 |  |
| Bartal, 2019 | Basal insulin NPH vs. glargine or detemir - (no disaggregation of analogs) | Preterm birth | 233 (n=119, NPH; n=114, analog) | aRR (95% CI) 0.93 (0.68-1.26), p=0.39 | Age, race, ethnicity, diabetes medications pre-pregnancy, metformin and year of delivery |
| Lin, 2020 | Type 2 diabetes switching from pre-pregnancy metformin to insulin in pregnancy <12 weeks gestation | Preterm birth | 318 | aOR (95% CI) 1.21 (0.84–1.75), p=NR | Age, duration of type 2 diabetes, hypertension, hyperlipidaemia, retinopathy, and aspirin use |
| Lin, 2020 | Type 2 diabetes <3 years duration - switching from pre-pregnancy metformin to insulin in pregnancy <12 weeks gestation | Preterm birth | 154 | aOR (95% CI) 1.08 (0.60–1.95), p=NR | Age, duration of type 2 diabetes, hypertension, hyperlipidaemia, retinopathy, and aspirin use |
| Nadeau, 2021 | Insulin dose ≥2 units/kg at delivery | Preterm birth | 160 | OR (95% CI) 1.1829 (0.6003 to 2.3308), p=0.6275 |  |
| Lin, 2020 | Type 2 diabetes switching from pre-pregnancy metformin to insulin in pregnancy <12 weeks gestation | Preterm birth | 318 | aOR (95% CI) 0.81 (0.33–2.02), p=NR | Age, duration of type 2 diabetes, hypertension, hyperlipidaemia, retinopathy, and aspirin use |
| ***Outcome – Respiratory Distress Syndrome*** | | | | | |
| Bartal, 2019 | Basal insulin NPH vs. glargine or detemir - (no disaggregation of analogs) | Respiratory Distress Syndrome | 233 (n=119, NPH; n=114, analog) | aOR (95% CI) 0.58 (0.22-1.55),p=0.24 | Age, race, ethnicity, diabetes medications pre-pregnancy, metformin and year of delivery |
| Nadeau, 2021 | Insulin dose ≥2 units/kg at delivery | Respiratory Distress Syndrome | 160 | OR (95% CI) 1.0755 (0.5275 to 2.1927), p=0.8413 |  |
| ***Outcome - SGA*** | | | | | |
| Lin, 2020 | Type 2 diabetes switching from pre-pregnancy metformin to insulin in pregnancy <12 weeks gestation | SGA & related growth concerns | 318 | aOR (95% CI) 1.32 (0.76–2.29), p=NR | Age, duration of type 2 diabetes, hypertension, hyperlipidaemia, retinopathy, and aspirin use |
| Lin, 2020 | Type 2 diabetes <3 years duration - switching from pre-pregnancy metformin to insulin in pregnancy <12 weeks gestation | SGA & related growth concerns | 154 | aOR (95% CI) 1.26 (0.49–3.22), p=NR | Age, duration of type 2 diabetes, hypertension, hyperlipidaemia, retinopathy, and aspirin use |
| Nadeau, 2021 | Insulin dose ≥2 units/kg at delivery | SGA & related growth concerns | 160 | OR (95% CI) 0.9130 (0.1976 to 4.2186), p= 0.9073 |  |
| ***Outcome - Shoulder dystocia*** | | | | | |
| Bartal, 2019 | Basal insulin NPH vs. glargine or detemir - (no disaggregation of analogs) | Shoulder dystocia | 233 (n=119, NPH; n=114, analog) | aOR (95% CI) 1.41 (0.31-6.43), p=0.66 | Age, race, ethnicity, diabetes medications pre-pregnancy, metformin and year of delivery |
| Kapustin, 2022 | Insulin treated type 2 diabetes | Shoulder dystocia |  | OR (95% CI)0.55 (0.15 to 2.12), p=0.388 |  |
| ***Outcome – Gestational weight gain*** | | | | | |
| Asbjornsdottir, 2013 | Insulin treatment at first visit | GWG <5kgs | 58 | OR (95% CI) 0.8800 (0.2518 to 3.0755), p= 0.8413 |  |
| Asbjornsdottir, 2013 | Insulin treatment at first visit | GWG >5kgs | 58 | OR (95% CI) 2.4306  (0.6264 to 9.4312), p= 0.1992 |  |

Abbreviations: aOR (Adjusted Odds Ratio); APGAR (refers to the scoring system used by medical professionals to assess the health of a newborn baby shortly after birth and stands for Appearance, Pulse, Grimace, Activity, and Respiration); aRR (Adjusted Risk Ratio); BGL (Blood Glucose Level); BMI (Body Mass Index); CI (Confidence Interval); GWG (Gestational Weight Gain); HBA1c (HBA1c (haemoglobin A1c, a blood test that measures the average blood sugar level over the past two to three months); IVI (Intravenous Insulin); IQR (Inter Quartile Range); LGA (Large for Gestational Age); NICU (Neonatal Intensive Care Unit); NPH (neutral protamine hagedorn); OR (Odds Ratio); SD (Standard Deviation); SGA (Small for Gestational Age).

**Supplementary Table 7**. Findings on associations between other oral hypoglycaemic medications and maternal and child health outcomes

| **First author, year** | **Exposure description** | **Pregnancy outcome** | **Total sample size** | **Result** | **Confounder adjustments** |
| --- | --- | --- | --- | --- | --- |
| ***Sulfonylurea - Outcome - Macrosomia*** | | | | | |
| Feghali, 2017 | Use of oral hypoglycaemic agents use vs use of insulin | Macrosomia | 198 (67 on OHA and 131 on insulin) | aOR (95% CI) 0.48 (0.11-2.14), p=NS | Adjusted model for macrosomia and hypertensive disorders of pregnancy includes maternal age, race, pre-pregnancy BMI, chronic hypertension, disease duration, and first-trimester haemoglobin A1c. |
| Shannon, 2016 | Glyburide use vs no glyburide use | Macrosomia | 4166 | OR (95% CI) 1.93 (1.51–2.47), p<0.0001 |  |
| ***Various medications – Outcome - congenital anomaly*** | | | | | |
| Roland, 2005 | Oral hypoglycaemic use vs no oral hypoglycaemic use | Congenital anomaly | 146 | aOR (95% CI) 1.8 (1.0–3.3), p=0.04 | Ethnicity, maternal age, diabetes type, oral hypoglycaemic agents, insulin use, HbA1cin first trimester, body mass index (BMI), folic acid, pre-pregnancy care, gestational age and smoking at conception. |
| Feghali, 2017 | Oral hypoglycaemic use vs use of insulin | Congenital anomaly | 198 (67 on OHA and 131 on insulin) | OR (95% CI) 0.34 (0.07-1.56), p=NS |  |
| Cesta, 2023 | Sulfonylureas use vs Insulin | Cardiac malformation | 51826 | aOR (95% CI) 1.05 (0.75-1.48) | Birth year, maternal age, obesity, country. |
| Cesta, 2023 | Sulfonylureas use vs use of insulin | Any major congenital malformation | 51826 | aOR (95% CI) 1.18 (0.94-1.48) | Birth year, maternal age, obesity, country. |
| Cesta, 2023 | GLP-1 receptor agonists use vs use of insulin | Any major congenital malformation | 51826 | aOR (95% CI) 0.95 (0.72-1.26) | Birth year, maternal age, obesity, country. |
| Cesta, 2023 | GLP-1 receptor agonists use vs use of insulin | Cardiac malformation | 51826 | aOR (95% CI) 0.68 (0.42-1.12), | Birth year, maternal age, obesity, country. |
| Cesta, 2023 | DPP-4 inhibitors use vs use of insulin | Cardiac malformation | 51826 | aOR (95% CI) 0.90 (0.58-1.39), | Birth year, maternal age, obesity, country. |
| Cesta, 2023 | DPP-4 inhibitors use vs use of insulin | Any major congenital malformation | 51826 | aOR (95% CI) 0.83 (0.64-1.06), | Birth year, maternal age, obesity, country. |
| Cesta, 2023 | SGLT2 inhibitors use vs use of insulin | Cardiac malformation | 51826 | aOR (95% CI) 1.10 (0.63-1.92), | Birth year, maternal age, obesity, country. |
| Cesta, 2023 | SGLT2 inhibitors use vs use of insulin | Any major congenital malformation | 51826 | aOR (95% CI) 0.98 (0.65-1.46) | Birth year, maternal age, obesity, country. |
| ***Outcome - Other*** | | | | | |
| Feghali, 2017 | Use of oral hypoglycaemic agents use vs use of insulin | Gestational Age at delivery | 198 (67 on OHA and 131 on insulin) | Mean (SD) Exposed group 37.3 (±2.6) vs Unexposed group 36.8 (±2.6), p=NS |  |
| Feghali, 2017 | Use of oral hypoglycaemic agents use vs use of insulin | Preterm birth <37 weeks | 198 (67 on OHA and 131 on insulin) | aOR (95% CI) 0.92 (0.44-1.92), p=NS | Adjusted model for prematurity includes nulliparity, pre-pregnancy body mass index (BMI), gestational weight gain, and glycemic control |
| Feghali, 2017 | Use of oral hypoglycaemic agents use vs use of insulin | Birthweight (g) | 198 (67 on OHA and 131 on insulin) | Mean (SD) Exposed group 3,177 (±685) vs Unexposed group 3,243 (±818), p=NS |  |
| Feghali, 2017 | Use of oral hypoglycaemic agents use vs use of insulin | SGA | 198 (67 on OHA and 131 on insulin) | OR (95% CI) 1.58 (0.56-4.45), p=NS |  |
| Feghali, 2017 | Use of oral hypoglycaemic agents use vs use of insulin | LGA | 198 (67 on OHA and 131 on insulin) | OR (95% CI) 0.44 (0.20-0.99), p=0.05 |  |
| Feghali, 2017 | Use of oral hypoglycaemic agents use vs use of insulin | Hypertensive disorders of pregnancy | 198 (67 on OHA and 131 on insulin) | aOR (95% CI) 0.72 (0.27-1.92), p=NS | Adjusted model for macrosomia and hypertensive disorders of pregnancy includes maternal age, race, pre-pregnancy BMI, chronic hypertension, disease duration, and first-trimester haemoglobin A1c. |
| Feghali, 2017 | Use of oral hypoglycaemic agents use vs use of insulin | Neonatal morbidity | 198 (67 on OHA and 131 on insulin) | aOR (95% CI) 0.89 (0.35-2.23), p=NS | Adjusted model for neonatal morbidity includes maternal age, race, pre-pregnancy BMI, chronic hypertension, disease duration, first-trimester haemoglobin A1c and prematurity |
| Feghali, 2017 | Use of oral hypoglycaemic agents use vs use of insulin | Caesarean delivery | 198 (67 on OHA and 131 on insulin) | OR (95% CI) 0.44 (0.24-0.80), p=0.008 |  |
| Feghali, 2017 | Use of oral hypoglycaemic agents use vs use of insulin | Primary caesarean delivery | 198 (67 on OHA and 131 on insulin) | OR (95% CI) 0.64 (0.34-1.21), p=NS |  |
| Feghali, 2017 | Use of oral hypoglycaemic agents use vs use of insulin | Stillbirth | 198 (67 on OHA and 131 on insulin) | OR (95% CI) 6.09 (0.62-59.75), p=NS |  |
| Feghali, 2017 | Use of oral hypoglycaemic agents use vs use of insulin | Shoulder dystocia | 198 (67 on OHA and 131 on insulin) | OR (95% CI) 2.00 (0.39-10.19), p=NS |  |
| Feghali, 2017 | Use of oral hypoglycaemic agents use vs use of insulin | NICU admission | 198 (67 on OHA and 131 on insulin) | OR (95% CI) 0.64 (0.33-1.25), p=NS |  |
| Feghali, 2017 | Use of oral hypoglycaemic agents use vs use of insulin | Neonatal hypoglycaemia | 198 (67 on OHA and 131 on insulin) | OR (95% CI) 0.73 (0.32-1.68), p=NS |  |
| Feghali, 2017 | Use of oral hypoglycaemic agents use vs use of insulin | RDS | 198 (67 on OHA and 131 on insulin) | OR (95% CI) 0.54 (0.19-1.54), p=NS |  |
| Feghali, 2017 | Use of oral hypoglycaemic agents use vs use of insulin | Hyperbilirubinaemia | 198 (67 on OHA and 131 on insulin) | OR (95% CI) 1.13 (0.45-2.85), p=NS |  |
| Feghali, 2017 | Use of oral hypoglycaemic agents use vs use of insulin | Gestational weight gain (lbs) | 198 (67 on OHA and 131 on insulin) | Mean (SD) Exposed group 22.4 (±18.8) vs Unexposed group 30.4 (±17.7), p=0.005 |  |
| Feghali, 2017 | Use of oral hypoglycaemic agents use vs use of insulin | insufficient gestational weight gain | 198 (67 on OHA and 131 on insulin) | OR (95% CI) 2.25 (1.05-4.86), p=0.04 |  |
| Feghali, 2017 | Use of oral hypoglycaemic agents use vs use of insulin | Excessive gestational weight gain | 198 (67 on OHA and 131 on insulin) | OR (95% CI) 0.44 (0.24-0.80), p=0.007 |  |

Abbreviations: CI (Confidence Interval); DPP-4 (dipeptidyl peptidase-4); GLP-1 (glucagon-like peptide-1); LGA (Large for Gestational Age); NPH (neutral protamine hagedorn); (OHA (Oral Hypoglycaemic Agents); OR (Odds Ratio); NS (Not Significant); RDS (Respiratory Distress Syndrome); SD (Standard Deviation); SGA (Small for Gestational Age); SGLT2 (sodium-glucose cotransporter-2).

**Supplementary Table 8**. Findings on associations between other non-diabetes medications and maternal and child health outcomes

| **First author, year** | **Exposure description** | **Pregnancy outcome** | **Total sample size** | **Result** | **Confounder adjustments** |
| --- | --- | --- | --- | --- | --- |
| ***Aspirin use - Outcome gestational age at delivery*** | | | | | |
| Søholm, 2021 | Prophylactic aspirin antenatally vs no prophylactic aspirin | Preterm delivery (<37 weeks) | 86 | n (%) Exposed group 6 (35) vs Unexposed group 15 (22), p=NS | Gestational age and sex |
| Søholm, 2021 | Asthma medication early pregnancy vs no asthma medication | Preterm delivery (<37 weeks) | 49 | aOR (95% CI) 2.59 (0.38 to 17.92), p=NS | Gestational age and sex |
| Søholm, 2021 | Antidepressant medication in early pregnancy vs no antidepressant medication | Preterm delivery (<37 weeks) | 49 | aOR (95% CI) 0.44 (0.02 to 9.19), p=NS | Gestational age and sex |
| Søholm, 2021 | Thyroid medication early pregnancy vs no thyroid medication | Preterm delivery (<37 weeks) | 49 | aOR (95% CI) 0.32 (0.04 to 2.87), p=NS | Gestational age and sex |
| Do, 2021 | Prophylactic aspirin antenatally vs no prophylactic aspirin | Gestational age at delivery (weeks) | 153 | Median (IQR) Exposed group 263 (259 - 269) vs Unexposed group 263 (260 - 270), p=NS |  |
| Do, 2021 | Prophylactic aspirin antenatally vs no prophylactic aspirin | Preterm delivery (<37 weeks) | 153 | OR (95% CI) 1.27 (0.59 to 2.76), p=NS |  |
| Do, 2021 | Prophylactic aspirin antenatally vs no prophylactic aspirin | Preterm delivery (<34 weeks) | 153 | OR (95% CI) 1.30 (0.31 to 5.41), p=NS |  |
| ***Other outcomes*** | | | | | |
| Do, 2021 | Prophylactic aspirin antenatally vs no prophylactic aspirin | Birth weight (g) | 153 | Mean ± SD Exposed group 3319 ± 745 vs Unexposed group 3189 ± 692, p=NS |  |
| Do, 2021 | Prophylactic aspirin antenatally vs no prophylactic aspirin | Birth weight (z score) | 153 | Mean ± SD Exposed group 0.6 ± 1.8 vs Unexposed group 0.3 ± 1.3, p=NS | Gestational age and sex |
| Do, 2021 | Prophylactic aspirin antenatally vs no prophylactic aspirin | SGA | 153 | aOR (95% CI) 1.71 (0.64 to 4.62), p=NS | Birth weight < 10th percentile |
| Do, 2021 | Prophylactic aspirin antenatally vs no prophylactic aspirin | LGA | 153 | aOR (95% CI) 2.14 (1.02 to 4.51), p=NS | Birth weight >90th percentile |
| Do, 2021 | Prophylactic aspirin antenatally vs no prophylactic aspirin | Preeclampsia | 153 | OR (95% CI) 1.33 (0.50 to 3.55), p=NS |  |
| Do, 2021 | Prophylactic aspirin antenatally vs no prophylactic aspirin | Delivery with Preeclampsia <37 weeks | 153 | OR (95% CI) 2.39 (0.67 to 8.54), p=NS |  |
| Do, 2021 | Prophylactic aspirin antenatally vs no prophylactic aspirin | Delivery with Preeclampsia <34 weeks | 153 | OR (95% CI) 6.41 (0.30 to 135.71), p=NS |  |
| Do, 2021 | Prophylactic aspirin antenatally vs no prophylactic aspirin | Gestational hypertension | 153 | OR (95% CI) 1.2252 (0.52 to 2.89), p=NS |  |

Abbreviations: CI (Confidence Interval); IQR (Inter-quartile Range); LGA (Large for Gestational Age); OR (Odds Ratio); SD (Standard Deviation); SGA (Small for Gestational Age).

**Supplementary Table 9**. Findings on associations between preconception care and maternal and child health outcomes

| **First author, year** | **Exposure description** | **Pregnancy outcome** | **Total sample size** | **Result** | **Confounder adjustments** |
| --- | --- | --- | --- | --- | --- |
| ***Outcome - HbA1c first trimester*** | | | | | |
| Cyganek, 2011 | Preconception care vs no preconception care | HbA1c first trimester (%) | 70 | Mean (95% CI) Exposed group 5.7 (4.5, 6.9) vs Unexposed group 6.4 (5.6, 7.3), p=0.02 |  |
| Kallas-Koeman, 2012 | Preconception care vs no preconception care | HbA1c at referral (%) | 186 | Mean ± SD Exposed group 7.5 ± 2.1 vs Unexposed group 7.3 ± 1.8, p=NS |  |
| Kallas-Koeman, 2012 | Preconception care vs no preconception care | HbA1c first trimester (%) | 114 | Mean ± SD Exposed group 7.6 ± 1.8 vs Unexposed group 6.4 ± 1.1, p=0.007 |  |
| Yamamoto, 2018 | Community-based preconception care vs before introduction of the preconception care programme | HbA1c at first contact (mmol/mol) | 308 (before PPC = 178, during PPC = 130) | Mean ± SD Exposed group 50.4 ± 14.8 vs Unexposed group 52.2 ± 14.9, p=NS |  |
| Yamamoto, 2018 | Community-based preconception care vs before introduction of the preconception care programme | HbA1c at first contact (%) | 308 (before PPC = 178, during PPC = 130) | Mean ± SD Exposed group 6.8 ± 3.5 vs Unexposed group 6.9 ± 3.5, p=NS |  |
| Murphy, 2010 | Regional preconception care programme vs no preconception care | HbA1c first trimester (%) | 271 | Median (IQR) Exposed group 6.4 (5.5-7.7) vs Unexposed group 6.8 (5.7-8.8), p=0.007 |  |
| Wong, 2013 | Specialist diabetes care prior to pregnancy vs No specialist diabetes care in 12 months prior to pregnancy (specialists included endocrinologists or general physicians with a special interest in diabetes) | HbA1c first trimester | 117 | Mean ± SD Exposed group 7.7 ± 2.5 vs Unexposed group 8.2 ± 2.4, p=NS |  |
| Egan, 2016 | Pre-pregnancy programme delivered using a standardized proforma according to NICE guidance vs not attending pre-pregnancy care programme | First trimester HbA1c <6.5% | 146 | OR (95% CI) 2.67 (1.25 to 5.72), P=0.01 |  |
| Egan, 2016 | Pre-pregnancy programme delivered using a standardized proforma according to NICE guidance vs not attending pre-pregnancy care programme | First trimester HbA1c <6.1% | 146 | OR (95% CI) 4.76 (2.16 to 10.46), p<0.001 |  |
| Yamamoto, 2018 | Community-based preconception care vs before introduction of the preconception care programme | First trimester HbA1c <= 48 mmol/mol | 308 | OR (95% CI) 1.77 (1.12 to 2.79), p=0.02 |  |
| ***Outcome - HbA1c second trimester*** | | | | | |
| Cyganek, 2011 | Preconception care vs no preconception care | HbA1c second trimester (%) | 70 | Mean (95% CI) Exposed group 5.5 (4.7, 6.4) vs Unexposed group 5.7 (5.0, 6.3), p=NS |  |
| Wong, 2013 | Specialist diabetes care prior to pregnancy vs No specialist diabetes care in 12 months prior to pregnancy (specialists included endocrinologists or general physicians with a special interest in diabetes) | HbA1c second trimester | 117 | Mean ± SD Exposed group 6.3 ± 1.3 vs Unexposed group 6.5 ± 1.4, p=NS |  |
| ***Outcome - HbA1c third trimester*** | | | | | |
| Cyganek, 2011 | Pre-pregnancy planning vs No pre-pregnancy planning | HbA1c third trimester (%) | 70 | Mean (95% CI) Exposed group 5.6 (5.1, 6.1) vs Unexposed group 5.6% (4.8, 6.3), p=NS |  |
| Murphy, 2010 | Regional preconception care programme vs no preconception care | HbA1c third trimester (%) | 271 | Median (IQR) Exposed group 6.1 (5.2-6.9) vs Unexposed group 6.2 (5.2-7.3), p=NS |  |
| Wong, 2013 | Specialist diabetes care prior to pregnancy vs No specialist diabetes care in 12 months prior to pregnancy (specialists included endocrinologists or general physicians with a special interest in diabetes) | HbA1c third trimester | 117 | Mean ± SD Exposed group 6.0 ± 0.6 vs Unexposed group 6.2 ± 0.7, p=NS |  |
| ***Outcome - Folic acid use preconception any dose*** | | | | | |
| Egan, 2016 | Pre-pregnancy programme delivered using a standardized proforma according to NICE guidance vs not attending pre-pregnancy care programme | Preconception folic acid use | 146 | OR (95% CI) 14.64 (3.35 to 63.95), p=0.0004 |  |
| Kallas-Koeman, 2012 | Preconception care vs no preconception care | Folic acid use at initial visit | 213 | OR (95% CI) 0.34 (0.17 to 0.71), p<0.01 |  |
| Yamamoto, 2018 | Community-based preconception care vs before introduction of the preconception care programme | Preconception folic acid any dose | 287 | OR (95% CI) 1.65 (1.03 to 2.66), p=0.04 |  |
| ***Outcome - Folic acid use preconception 5mg dose*** | | | | | |
| Yamamoto, 2018 | Community-based preconception care vs before introduction of the preconception care programme | Preconception folic acid 5mg | 284 | OR (95% CI) 2.34 (1.41 to 3.91), p=0.001 |  |
| Murphy, 2010 | Regional preconception care programme vs no preconception care | Preconception folic acid 5mg | 271 | OR (95% CI) 24.28 (10.64 to 55.42), p<0.001 |  |
| ***Outcome - Hypertensive disorder of pregnancy*** | | | | | |
| Morikawa, 2020 | Pre-pregnancy treatment vs no treatment pre-pregnancy | Hypertensive disorder of pregnancy | 109 | OR (95% CI) N/A, p=NS |  |
| Egan, 2016 | Pre-pregnancy programme delivered using a standardized proforma according to NICE guidance vs not attending pre-pregnancy care programme | Hypertensive disorder of pregnancy | 146 | OR (95% CI) 0.53 (0.20 to 1.340), p=NS |  |
| Egan, 2016 | Pre-pregnancy programme delivered using a standardized proforma according to NICE guidance vs not attending pre-pregnancy care programme | Preeclampsia | 146 | OR (95% CI) 0.54 (0.11 to 2.58), p=NS |  |
| ***Outcome - Macrosomia or high birthweight*** | | | | | |
| Shannon, | Seeing an endocrinologist prior to and during pregnancy vs No endocrinologist review | Macrosomia | 4166 | OR (95% CI) 0.92 (0.72 to 1.18), p=NS |  |
| Egan, 2016 | Pre-pregnancy programme delivered using a standardized proforma according to NICE guidance vs not attending pre-pregnancy care programme | Macrosomia | 129 | OR (95% CI) 1.44 (0.58 to 3.59), p=NS |  |
| Wong, 2013 | Specialist diabetes care prior to pregnancy vs No specialist diabetes care in 12 months prior to pregnancy (specialists included endocrinologists or general physicians with a special interest in diabetes) | Birth weight >90th centile | 117 | OR (95% CI) Exposed group 0.91 (0.34 - 2.43) vs Unexposed group, p=NS |  |
| ***Outcome - Delivery <37 weeks*** | | | | | |
| Wong, 2013 | Specialist diabetes care prior to pregnancy vs No specialist diabetes care in 12 months prior to pregnancy (specialists included endocrinologists or general physicians with a special interest in diabetes) | Preterm birth <37 weeks | 117 | OR (95% CI) 2.46 (0.66-9.21), p=NS |  |
| Egan, 2016 | Pre-pregnancy programme delivered using a standardized proforma according to NICE guidance vs not attending pre-pregnancy care programme | Preterm birth <37 weeks | 129 | OR (95% CI) 1.19 (0.39 to 3.68), p=NS |  |
| Yamamoto, 2018 | Community-based preconception care vs before introduction of the preconception care programme | Preterm birth <37 weeks | 295 | OR (95% CI) 1.47 (0.86 to 2.52), p=NS |  |
| ***Outcome - Delivery <34 weeks*** | | | | | |
| Egan, 2016 | Pre-pregnancy programme delivered using a standardized proforma according to NICE guidance vs not attending pre-pregnancy care programme | Preterm birth <34 weeks | 129 | OR (95% CI) 1.44 (0.34 to 6.09), p=NS |  |
| Yamamoto, 2018 | Community-based preconception care vs before introduction of the preconception care programme | Preterm birth <34 weeks | 295 | OR (95% CI) 0.66 (0.19 to 2.24), p=NS |  |
| Wong, 2013 | Specialist diabetes care prior to pregnancy vs No specialist diabetes care in 12 months prior to pregnancy (specialists included endocrinologists or general physicians with a special interest in diabetes) | Birth weight <10th centile | 117 | OR (95% CI) 0.36 (0.04-2.97), p=NS |  |
| ***Outcome - Congenital anomaly*** | | | | | |
| Egan, 2016 | Pre-pregnancy programme delivered using a standardized proforma according to NICE guidance vs not attending pre-pregnancy care programme | Congenital malformation | 146 | OR (95% CI) 1.42 (0.13 to 16.11), p=NS |  |
| Yamamoto, 2018 | Community-based preconception care vs before introduction of the preconception care programme | Congenital malformation | 289 | OR (95% CI) 1.40 (0.34 to 5.72), p=NS |  |
| Wong, 2013 | Specialist diabetes care prior to pregnancy vs No specialist diabetes care in 12 months prior to pregnancy (specialists included endocrinologists or general physicians with a special interest in diabetes) | Congenital anomaly | 117 | OR (95% CI) Exposed group 0.53 (0.06-4.56) vs Unexposed group, p=NS |  |
| Murphy, 2010 | Regional preconception care programme vs no preconception care | Congenital malformation | 222 | OR (95% CI) 0.18 (0.01 to 3.05), p=NS |  |
| ***Outcome - Perinatal mortality*** | | | | | |
| Yamamoto, 2018 | Community-based preconception care vs before introduction of the preconception care programme | Perinatal mortality | 282 | OR (95% CI) 2.21 (0.36 to 13.44), p=NS |  |
| Murphy, 2010 | Regional preconception care programme vs no preconception care | Perinatal mortality | 222 | OR (95% CI) 0.77 (0.04 to 16.35), p=NS |  |
| Wong, 2013 | Specialist diabetes care prior to pregnancy vs No specialist diabetes care in 12 months prior to pregnancy (specialists included endocrinologists or general physicians with a special interest in diabetes) | Perinatal death | 117 | OR (95% CI) 1.66 (0.47-5.84), p=NS |  |
| ***Outcome - Neonatal death*** | | | | | |
| Egan, 2016 | Pre-pregnancy programme delivered using a standardized proforma according to NICE guidance vs not attending pre-pregnancy care programme | Neonatal death | 146 | OR (95% CI) 2.79 (0.05 to 143.18), p=NS |  |
| Yamamoto, 2018 | Community-based preconception care vs before introduction of the preconception care programme | Neonatal death | 278 | OR (95% CI) 0.49 (0.02 to 12.15), p=NS |  |
| Murphy, 2010 | Regional preconception care programme vs no preconception care | Neonatal death | 222 | OR (95% CI) 3.90 (0.08 to 199.29), p=NS |  |
| ***Outcome - Stillbirth*** | | | | | |
| Egan, 2016 | Pre-pregnancy programme delivered using a standardized proforma according to NICE guidance vs not attending pre-pregnancy care programme | Stillbirth | 146 | OR (95% CI) 0.55 (0.03 to 11.67), p=NS |  |
| Yamamoto, 2018 | Community-based preconception care vs before introduction of the preconception care programme | Stillbirth | 299 | OR (95% CI) 4.20 (0.43 to 40.81), p=NS |  |
| Murphy, 2010 | Regional preconception care programme vs no preconception care | Stillbirth | 222 | OR (95% CI) 0.77 (0.04 to 16.35), p=NS |  |
| ***Outcome - Miscarriage*** | | | | | |
| Egan, 2016 | Pre-pregnancy programme delivered using a standardized proforma according to NICE guidance vs not attending pre-pregnancy care programme | Miscarriage | 146 | OR (95% CI) 0.93 (0.28 to 3.08), p=NS |  |
| Yamamoto, 2018 | Community-based preconception care vs before introduction of the preconception care programme | Miscarriage | 314 | OR (95% CI) 0.87 (0.28 to 2.72), p=NS |  |
| ***Outcome - LGA*** | | | | | |
| Egan, 2016 | Pre-pregnancy programme delivered using a standardized proforma according to NICE guidance vs not attending pre-pregnancy care programme | LGA | 129 | OR (95% CI) 1.79 (0.75 to 4.29), p=NS |  |
| Yamamoto, 2018 | Community-based preconception care vs before introduction of the preconception care programme | LGA | 295 | OR (95% CI) 1.22 (0.69 to 2.16), p=NS |  |
| Yamamoto, 2018 | Community-based preconception care vs before introduction of the preconception care programme | Extremely LGA | 295 | OR (95% CI) 1.12 (0.53 to 2.36), p=NS |  |
| ***Outcome - SGA*** | | | | | |
| Egan, 2016 | Pre-pregnancy programme delivered using a standardized proforma according to NICE guidance vs not attending pre-pregnancy care programme | SGA | 129 | OR (95% CI) 1.05 (0.26 to 4.22), p=NS |  |
| Yamamoto, 2018 | Community-based preconception care vs before introduction of the preconception care programme | SGA | 295 | OR (95% CI) 0.60 (0.30 to 1.20), p=NS |  |
| ***Outcome – maternal weight or BMI at booking*** | | | | | |
| Murphy, 2010 | Regional preconception care programme vs no preconception care | BMI at booking | 271 | Median (IQR) Exposed group 33.9 (26-40) vs Unexposed group 32.2 (24-43), p=NS |  |
| Yamamoto, 2018 | Community-based preconception care vs before introduction of the preconception care programme | BMI at booking | 318 | Mean ± SD Exposed group 33.9 ± 7.2 vs Unexposed group 34.3 ± 8.0, p=NS |  |
| Yamamoto, 2018 | Community-based preconception care vs before introduction of the preconception care programme | Weight at booking | 318 | Mean ± SD Exposed group 90.8 ± 21.5 vs Unexposed group 91.2 ± 24.5, p=NS |  |
| Yamamoto, 2018 | Community-based preconception care vs before introduction of the preconception care programme | BMI at booking: normal (<24.9 kg/m2) | 318 | OR (95% CI) 0.77 (0.38 to 1.59), p=NS |  |
| Yamamoto, 2018 | Community-based preconception care vs before introduction of the preconception care programme | BMI at booking: overweight (25.0-29.9 kg/m2) | 318 | OR (95% CI) 1.35 (0.76 to 2.38), p=NS |  |
| Yamamoto, 2018 | Community-based preconception care vs before introduction of the preconception care programme | BMI at booking: obese (=>30.0 kg/m2) | 318 | OR (95% CI) 0.91 (0.56 to 1.48), p=NS |  |
| ***Outcome - Other*** | | | | | |
| Morikawa, 2020 | Pre-pregnancy treatment vs no treatment pre-pregnancy | Fetal growth restriction | 109 | OR (95% CI) 340 (19.0-6077.0), p=NS |  |
| Egan, 2016 | Pre-pregnancy programme delivered using a standardized proforma according to NICE guidance vs not attending pre-pregnancy care programme | Excessive gestational weight gain | 129 | OR (95% CI) 1.48 (0.67 to 3.25), p=NS |  |
| Kallas-Koeman, 2012 | preconception care vs preconception care | Oral hypoglycemic agent use | 135 | OR (95% CI) 1.75 (0.60 to 5.12), p=NS |  |
| Kallas-Koeman, 2012 | preconception care vs preconception care | Insulin use | 135 | OR (95% CI) 1.53 (0.62 to 3.79), p=NS |  |
| Yamamoto, 2018 | Community-based preconception care vs before introduction of the preconception care programme | Termination of pregnancy | 314 | OR (95% CI) 0.28 (0.01 to 5.80), p=NS |  |
| Egan, 2016 | Pre-pregnancy programme delivered using a standardized proforma according to NICE guidance vs not attending pre-pregnancy care programme | Shoulder dystocia | 146 | OR (95% CI) 0.55 (0.03 to 11.67), p=NS |  |
| Yamamoto, 2018 | Community-based preconception care vs before introduction of the preconception care programme | Gestational age at delivery | 295 | Mean ± SD Exposed group 37.3 ± 1.8 vs Unexposed group 37.4 ± 1.9, p=NS |  |
| Yamamoto, 2018 | Community-based preconception care vs before introduction of the preconception care programme | Birthweight | 299 | Mean ± SD Exposed group 3249.7 ± 655.1 vs Unexposed group 3235.2 ± 665.5, p=NS |  |
| Egan, 2016 | Pre-pregnancy programme delivered using a standardized proforma according to NICE guidance vs not attending pre-pregnancy care programme | Caesarean | 129 | OR (95% CI) 2.16 (0.93 to 5.01), p=NS |  |
| Egan, 2016 | Pre-pregnancy programme delivered using a standardized proforma according to NICE guidance vs not attending pre-pregnancy care programme | Neonatal hypoglycaemia requiring hospital admission | 127 | OR (95% CI) 0.79 (0.16 to 3.98), p=NS |  |
| Egan, 2016 | Pre-pregnancy programme delivered using a standardized proforma according to NICE guidance vs not attending pre-pregnancy care programme | Neonatal intensive care | 127 | OR (95% CI) 0.66 (0.29 to 1.49), p=NS |  |
| Yamamoto, 2018 | Community-based preconception care vs before introduction of the preconception care programme | Gestational age at booking | 318 | Mean ± SD Exposed group 9.8 ± 4.9 vs Unexposed group 10.5 ± 4.5, p=NS |  |
| Yamamoto, 2018 | Community-based preconception care vs before introduction of the preconception care programme | Booking prior to 8 weeks | 317 | OR (95% CI) 1.33 (0.84 to 2.11), p=NS |  |
| Yamamoto, 2018 | Community-based preconception care vs before introduction of the preconception care programme | On at least one potentially harmful medication | 300 | OR (95% CI) 0.73 (0.38 to 1.42), p=NS |  |
| Yamamoto, 2018 | Community-based preconception care vs before introduction of the preconception care programme | Two or more potentially harmful medications | 300 | OR (95% CI) 0.36 (0.07 to 1.76), p=NS |  |
| Yamamoto, 2018 | Community-based preconception care vs before introduction of the preconception care programme | Optimal pregnancy preparation (first HbA1c <=48 mmol/mol, on folic acid 5mg daily prior to last menstrual period, booking at <=8 weeks gestation, and no harmful medications prior to last menstrual period | 257 | OR (95% CI) 2.90 (1.21 to 6.93), p=0.02 |  |
| Yamamoto, 2018 | Community-based preconception care vs before introduction of the preconception care programme | Live birth | 314 | OR (95% CI) 0.98 (0.38 to 2.52), p=NS |  |
| Yamamoto, 2018 | Community-based preconception care vs before introduction of the preconception care programme | Advanced neonatal care | 295 | OR (95% CI) 1.39 (0.86 to 2.26), p=NS |  |
| Yamamoto, 2018 | Community-based preconception care vs before introduction of the preconception care programme | Serious adverse outcome (malformation with or without termination of pregnancy, stillbirth or neonatal death) | 276 | OR (95% CI) 1.73 (0.57 to 5.29), p=NS |  |
| Wong, 2013 | Specialist diabetes care prior to pregnancy vs No specialist diabetes care in 12 months prior to pregnancy (specialists included endocrinologists or general physicians with a special interest in diabetes) | NICU admission | 117 | OR (95% CI) Exposed group 2.07 (0.48-8.97), p=NS |  |
| Wong, 2013 | Specialist diabetes care prior to pregnancy vs No specialist diabetes care in 12 months prior to pregnancy (specialists included endocrinologists or general physicians with a special interest in diabetes) | NICU Length of Stay (days) | 117 | Mean ± SD Exposed group 5.6 ± 1.9 vs Unexposed group 6.8 ± 7.4, p=NS |  |
| Wong, 2013 | Specialist diabetes care prior to pregnancy vs No specialist diabetes care in 12 months prior to pregnancy (specialists included endocrinologists or general physicians with a special interest in diabetes) | Insulin therapy pre-conception | 117 | OR (95% CI) 0.13 (0.05-0.34) , p<0.0001 |  |
| Wong, 2013 | Specialist diabetes care prior to pregnancy vs No specialist diabetes care in 12 months prior to pregnancy (specialists included endocrinologists or general physicians with a special interest in diabetes) | Pregnancy planning | 117 | OR (95% CI) 0.67 (0.27-1.64), p=NS |  |
| Wong, 2013 | Specialist diabetes care prior to pregnancy vs No specialist diabetes care in 12 months prior to pregnancy (specialists included endocrinologists or general physicians with a special interest in diabetes) | Smoking | 117 | OR (95% CI) 0.25 (0.03 - 1.97), p=NS |  |
| Wong, 2013 | Specialist diabetes care prior to pregnancy vs No specialist diabetes care in 12 months prior to pregnancy (specialists included endocrinologists or general physicians with a special interest in diabetes) | Vit D (25-OH, nmol/L) | 117 | Mean ± SD Exposed group 63.6 ± 28.0 vs Unexposed group 48.9 ± 19.9, p=0.02 |  |
| Wong, 2013 | Specialist diabetes care prior to pregnancy vs No specialist diabetes care in 12 months prior to pregnancy (specialists included endocrinologists or general physicians with a special interest in diabetes) | Gestational weight gain (Kg) | 117 | Mean ± SD Exposed group 8.3 ± 15.7 vs Unexposed group 15 ± 9.5, p=0.02 |  |

Abbreviations: BMI (Body Mass Index); CI (Confidence Interval); SD (Standard Deviation); HBA1c (haemoglobin A1c, a blood test that measures the average blood sugar level over the past two to three months); IQR (Inter-quartile Range); LGA (Large for Gestational Age); NICE (National Institute for Health and Care Excellence); OR (Odds Ratio); PPC (Pre-pregnancy Care; SGA (Small for Gestational Age).

**Supplementary Table 10**. Findings on associations between antenatal care and maternal and child health outcomes

| **First author, year** | **Exposure description** | **Pregnancy outcome** | **Total sample size** | **Result** | **Confounder adjustments** |
| --- | --- | --- | --- | --- | --- |
| ***Outcome - Pre-eclampsia*** | | | | | |
| Allen, 2018 | Antenatal care: no antenatal care vs. presented in 1st trimester | Preeclampsia | 33,777 | aOR (95% CI): 1.21 (0.73–1.98)  P=0.46 | Parity, maternal age, maternal race/ethnicity, maternal education level, insurance type and chronic hypertension |
| Allen, 2018 | Antenatal care: presented in 3rd trimester vs. presented in the 1st trimester | Preeclampsia | 33,777 | aOR (95% CI): 1.00 (0.75–1.34)  P=0.997 | Parity, maternal age, maternal race/ethnicity, maternal education level, insurance type and chronic hypertension |
| Allen, 2018 | Antenatal care: no antenatal care vs. presented in 1st trimester | Preeclampsia (term delivery) | 33,777 | aOR (95% CI): 1.96 (1.12–3.43)  P=0.02 | Parity, maternal age, maternal race/ethnicity, maternal education level, insurance type and chronic hypertension |
| Allen, 2018 | Antenatal care: presented in 3rd trimester vs. presented in the 1st trimester | Preeclampsia (term delivery) | 33,777 | aOR (95% CI): 1.22 (0.86–1.74)  P=0.26 | Parity, maternal age, maternal race/ethnicity, maternal education level, insurance type and chronic hypertension |
| Oppermann, 2020 | Planned pregnancy: assumed, not explicit vs unplanned pregnancy | Preeclampsia | 128 | OR (95% CI): 0.11 (0.02-0.63)  P=0.01 |  |
| Perichart-Perera, 2009 | Medical Nutrition Therapy (MNT) programme: Individual nutrition counselling with an intensive education component vs. routine care (historical reference cohort) | Preeclampsia | 96 (n=47 control, n=49 MNT) | OR (95% CI): 0.12 (0.01-1.00)  P=0.02 |  |
| ***Outcome - Caesarean delivery*** | | | | | |
| Allen, 2018 | Antenatal care: no antenatal care vs. presented in 1st trimester | Caesarean delivery | 33,777 | aOR (95% CI): 0.82 (0.59–1.13)  P=0.22 | Parity, maternal age, maternal race/ethnicity, maternal education level, insurance type and chronic hypertension |
| Allen, 2018 | Antenatal care: presented in 3rd trimester vs. presented in the 1st trimester | Caesarean delivery | 33,777 | aOR (95% CI): 0.93 (0.77–1.11)  P=0.40 | Parity, maternal age, maternal race/ethnicity, maternal education level, insurance type and chronic hypertension |
| Allen, 2018 | Antenatal care: no antenatal care vs. presented in 1st trimester | Caesarean delivery | 33,777 | aOR (95% CI): 0.89 (0.62–1.29)  P=0.55 | Parity, maternal age, maternal race/ethnicity, maternal education level, insurance type and chronic hypertension |
| Allen, 2018 | Antenatal care: presented in 3rd trimester vs. presented in the 1st trimester | Caesarean delivery | 33,777 | aOR (95% CI): 0.93 (0.75–1.15)  P=0.52 | Parity, maternal age, maternal race/ethnicity, maternal education level, insurance type and chronic hypertension |
| Stafl, 2023 | One or more missed appointments vs. no missed appointments | Caesarean delivery | 902 | aOR (95% CI): 0.89 (0.66-1.19)  P=NR | Maternal age, smoking, Pampalon Maternal Deprivation Index and pre-pregnancy weight ≥ 91 kg |
| Ásbjörnsdóttir, 2019 | Motivational interviewing (Intervention cohort) vs. routine care (historical reference cohort) | Caesarean delivery | 189 | OR (95% CI): 0.83 (0.47-1.47)  P=0.52 |  |
| ***Preterm delivery*** | | | | | |
| Allen, 2018 | Antenatal care: no antenatal care vs. presented in 1st trimester | Preterm delivery | 33,777 | aOR (95% CI): 1.55 (1.03–2.32)  P=0.03 | Parity, maternal age, maternal race/ethnicity, maternal education level, insurance type and chronic hypertension |
| Allen, 2018 | Antenatal care: presented in 3rd trimester vs. presented in the 1st trimester | Preterm delivery | 33,777 | aOR (95% CI): 1.52 (1.22–1.89)  P<0.0001 | Parity, maternal age, maternal race/ethnicity, maternal education level, insurance type and chronic hypertension |
| Stafl, 2023 | One or more missed appointments vs. no missed appointments | Preterm delivery | 902 | aOR (95% CI): 0.98 (0.68-1.42) | Maternal age, smoking, Pampalon Maternal Deprivation Index and pre-pregnancy weight ≥ 91 kg |
| Stafl, 2023 | One or more missed appointments vs. no missed appointments | Early preterm delivery | 902 | aOR (95% CI): 0.49 (0.22-1.11) | Maternal age, smoking, Pampalon Maternal Deprivation Index and pre-pregnancy weight ≥ 91 kg |
| Ásbjörnsdóttir, 2019 | Motivational interviewing (Intervention cohort) vs. routine care (historical reference cohort) | Preterm delivery (<37 weeks) | 189 | OR (95% CI): 1.16 (0.55-2.42)  P=0.70 |  |
| Perichart-Perera, 2009 | Medical Nutrition Therapy (MNT) programme: Individual nutrition counselling with an intensive education component vs. routine care (historical reference cohort) | Prematurity (<37 weeks) | 96 (n=47 control, n=49 MNT) | OR (95% CI): 1.43 (0.42-4.91)  P=0.56 |  |
| ***Outcome - Macrosomia/ LGA*** | | | | | |
| Allen, 2018 | Antenatal care: no antenatal care vs. presented in 1st trimester | Macrosomia | 33,777 | aOR (95% CI): 1.33 (0.91–1.95)  P=0.14 | Parity, maternal age, maternal race/ethnicity, maternal education level, insurance type and chronic hypertension |
| Allen, 2018 | Antenatal care: presented in 3rd trimester vs. presented in the 1st trimester | Macrosomia | 33,777 | aOR (95% CI): 1.02 (0.81–1.28)  P=0.87 | Parity, maternal age, maternal race/ethnicity, maternal education level, insurance type and chronic hypertension |
| Allen, 2018 | Antenatal care: no antenatal care vs. presented in 1st trimester | Macrosomia (term delivery) | 33,777 | aOR (95% CI): 1.46 (0.96–2.20)  P=0.08 | Parity, maternal age, maternal race/ethnicity, maternal education level, insurance type and chronic hypertension |
| Allen, 2018 | Antenatal care: presented in 3rd trimester vs. presented in the 1st trimester | Macrosomia (term delivery) | 33,777 | aOR (95% CI): 1.08 (0.84–1.39)  P=0.53 | Parity, maternal age, maternal race/ethnicity, maternal education level, insurance type and chronic hypertension |
| Stafl, 2023 | One or more missed appointments vs. no missed appointments | LGA | 902 | aOR (95% CI): 1.61 (1.13-2.28)  P<0.05 | Maternal age, smoking, Pampalon Maternal Deprivation Index and pre-pregnancy weight ≥ 91 kg |
| Stafl, 2023 | One or more missed appointments vs. no missed appointments | Extreme LGA | 902 | aOR (95% CI): 1.69 (1.02-2.81)  P=NR | Maternal age, smoking, Pampalon Maternal Deprivation Index and pre-pregnancy weight ≥ 91 kg |
| Ásbjörnsdóttir, 2019 | Motivational interviewing (Intervention cohort) vs. routine care (historical reference cohort) | MNT (Medical Nutrition Therapy)  (Nordic curves) | 189 | aOR (95% CI): 0.56 (0.29- 1.09)  P=0.09 | Gestational age and infant sex |
| Perichart-Perera, 2009 | Medical Nutrition Therapy (MNT) programme: Individual nutrition counselling with an intensive education component vs. routine care (historical reference cohort) | Macrosomia (>4000g) | 96 (n=47 control, n=49 MNT) | OR (95% CI): 0.35 (0.06-1.98)  P=0.22 |  |
| ***Outcome - SGA*** | | | | | |
| Stafl, 2023 | One or more missed appointments vs. no missed appointments | SGA | 902 | aOR (95% CI): 0.88 (0.51-1.51)  P=NR | Maternal age, smoking, Pampalon Maternal Deprivation Index and pre-pregnancy weight ≥ 91 kg |
| Ásbjörnsdóttir, 2019 | Motivational interviewing (Intervention cohort) vs. routine care (historical reference cohort) | SGA | 189 | aOR (95% CI): 2.23 (0.74- 6.68)  P=0.15 | Gestational age and infant sex |
| ***Outcome - Shoulder dystocia*** | | | | | |
| Allen, 2018 | Antenatal care: no antenatal care vs. presented in 1st trimester | Shoulder dystocia | 33,777 | aOR (95% CI): 1.60 (0.70–3.65)  P=0.26 | Parity, maternal age, maternal race/ethnicity, maternal education level, insurance type and chronic hypertension |
| Allen, 2018 | Antenatal care: presented in 3rd trimester vs. presented in the 1st trimester | Shoulder dystocia | 33,777 | aOR (95% CI): 1.30 (0.78–2.16)  P=0.31 | Parity, maternal age, maternal race/ethnicity, maternal education level, insurance type and chronic hypertension |
| Allen, 2018 | Antenatal care: no antenatal care vs. presented in 1st trimester | Shoulder dystocia | 33,777 | aOR (95% CI): 1.57 (0.64–3.89)  P=0.32 | Parity, maternal age, maternal race/ethnicity, maternal education level, insurance type and chronic hypertension |
| Allen, 2018 | Antenatal care: presented in 3rd trimester vs. presented in the 1st trimester | Shoulder dystocia | 33,777 | aOR (95% CI): 1.28 (0.73–2.26)  P=0.38 | Parity, maternal age, maternal race/ethnicity, maternal education level, insurance type and chronic hypertension |
| Ásbjörnsdóttir, 2019 | Motivational interviewing (Intervention cohort) vs. routine care (historical reference cohort) | Shoulder dystocia | 189 | OR (95% CI): 0.95 (0.06 to 15.38)  P=0.97 |  |
| ***Outcome - Intrauterine/ neonatal death*** | | | | | |
| Allen, 2018 | Antenatal care: no antenatal care vs. presented in 1st trimester | Intrauterine fetal death | 33,777 | aOR (95% CI): 11.37 (6.10–21.16)  P<0.0001 | Parity, maternal age, maternal race/ethnicity, maternal education level, insurance type and chronic hypertension |
| Allen, 2018 | Antenatal care: presented in 3rd trimester vs. presented in the 1st trimester | Intrauterine fetal death | 33,777 | aOR (95% CI): 5.60 (3.49–8.97)  P<0.0001 | Parity, maternal age, maternal race/ethnicity, maternal education level, insurance type and chronic hypertension |
| Allen, 2018 | Antenatal care: no antenatal care vs. presented in 1st trimester | Intrauterine fetal death (term delivery) | 33,777 | aOR (95% CI): 18.93 (8.69–41.23)  P<0.0001 | Parity, maternal age, maternal race/ethnicity, maternal education level, insurance type and chronic hypertension |
| Allen, 2018 | Antenatal care: presented in 3rd trimester vs. presented in the 1st trimester | Intrauterine fetal death (term delivery) | 33,777 | aOR (95% CI): 8.66 (4.67–16.02)  P<0.0001 | Parity, maternal age, maternal race/ethnicity, maternal education level, insurance type and chronic hypertension |
| Ásbjörnsdóttir, 2019 | Motivational interviewing (Intervention cohort) vs. routine care (historical reference cohort) | Perinatal mortality | 189 | OR (95% CI): 0.95 (0.06 to 15.38)  P=0.97 |  |
| Perichart-Perera, 2009 | Medical Nutrition Therapy (MNT) programme: Individual nutrition counselling with an intensive education component vs. routine care (historical reference cohort) | Intrauterine fetal death | 96 (n=47 control, n=49 MNT) | OR (95% CI): 0.96 (0.12-1.09)  P=0.96 |  |
| Perichart-Perera, 2009 | Medical Nutrition Therapy (MNT) programme: Individual nutrition counselling with an intensive education component vs. routine care (historical reference cohort) | Neonatal death | 96 (n=47 control, n=49 MNT) | OR (95% CI): 0.13 (0.01 to 2.56)  P=0.18 |  |
| ***Outcome - Glycaemic control - mother/ neonate*** | | | | | |
| Sushko, 2023 | Self-care (diet, general) using Summary of Diabetes Self-Care Activities (SDSCA) scale | Improvement in HbA1C | 56 | Mean change (95% CI): -0.04 (-0.03 to 0.15)  P=0.67 | Participant age, diabetes duration, ethnicity, education level, household income, and insurance coverage |
| Sushko, 2023 | Self-care (diet, specific) using Summary of Diabetes Self-Care Activities (SDSCA) scale | Improvement in HbA1C | 56 | Mean change (95% CI): -0.05 (-0.22 to 0.12)  P=0.58 | Participant age, diabetes duration, ethnicity, education level, household income, and insurance coverage |
| Sushko, 2023 | Self-care (exercise) using Summary of Diabetes Self-Care Activities (SDSCA) scale | Improvement in HbA1C | 56 | Mean change (95% CI): -0.06 (-0.19 to 0.08)  P=0.43 | Participant age, diabetes duration, ethnicity, education level, household income, and insurance coverage |
| Sushko, 2023 | Self-care (glucose monitoring) using Summary of Diabetes Self-Care Activities (SDSCA) scale | Improvement in HbA1C | 56 | Mean change (95% CI): 0.09 (-0.08 to 0.28)  P=0.28 | Participant age, diabetes duration, ethnicity, education level, household income, and insurance coverage |
| Sushko, 2023 | Self-care (foot care) using Summary of Diabetes Self-Care Activities (SDSCA) scale | Improvement in HbA1C | 56 | Mean change (95% CI): 0.06 (-0.05 to 0.18)  P=0.28 | Participant age, diabetes duration, ethnicity, education level, household income, and insurance coverage |
| Murphy, 2021 | First antenatal contact: after 10 weeks vs. before 10 weeks | late pregnancy HbA1c <6.5% (48 mmol/mol) | Not reported | aOR (95% CI): 0.77 (0.66-0.91) | Diabetes duration, first trimester HbA1c |
| Ásbjörnsdóttir, 2019 | Motivational interviewing (Intervention cohort) vs. routine care (historical reference cohort) | HbA1c at early pregnancy visit (%) | 189 | Mean (SD) (HbA1c %):  Motivational interviewing: 6.7 (1.1)  Routine care: 6.5 (1.3)  P=0.32 |  |
| Ásbjörnsdóttir, 2019 | Motivational interviewing (Intervention cohort) vs. routine care (historical reference cohort) | HbA1c at early pregnancy visit (mmol/mol) | 189 | Mean (SD) (HbA1c mmol/mol):  Motivational interviewing: 50 (12)  Routine care: 48 (14)  P=0.32 |  |
| Ásbjörnsdóttir, 2019 | Motivational interviewing (Intervention cohort) vs. routine care (historical reference cohort) | HbA1c at late pregnancy visit (%) | 189 | Mean (SD) (HbA1c %):  Motivational interviewing: 5.9 (0.5)  Routine care: 6.0 (0.6)  P=0.34 |  |
| Ásbjörnsdóttir, 2019 | Motivational interviewing (Intervention cohort) vs. routine care (historical reference cohort) | HbA1c at late pregnancy visit  (mmol/mol) | 189 | Mean (SD) (HbA1c mmol/mol):  Motivational interviewing: 41 (6)  Routine care: 42 (7)  P=0.34 |  |
| Ásbjörnsdóttir, 2019 | Motivational interviewing (Intervention cohort) vs. routine care (historical reference cohort) | Difference HbA1c (%) | 189 | Median (IQR) (HbA1c %):  Motivational interviewing: -0.6 (-1.3, -0.2)  Routine care: -0.2 (-1.0, 0.1)  P=0.01 |  |
| Ásbjörnsdóttir, 2019 | Motivational interviewing (Intervention cohort) vs. routine care (historical reference cohort) | Difference HbA1c (mmol/mol) | 189 | Median (IQR) (HbA1c mmol/mol):  Motivational interviewing: -7 (-14, -2)  Routine care: -2 (-11, 1)  P=0.01 |  |
| Ásbjörnsdóttir, 2019 | Motivational interviewing (Intervention cohort) vs. routine care (historical reference cohort) | Neonatal hypoglycemia (<2.2 mmol/L) | 189 | OR (95% CI): 1.02 (0.46 to 2.25)  P=0.96 |  |
| Ásbjörnsdóttir, 2019 | Motivational interviewing (Intervention cohort) vs. routine care (historical reference cohort) | Ketonuria at late pregnancy visit | 189 | OR (95% CI): 0.62 (0.10 to 3.83)  P=0.61 |  |
| Ásbjörnsdóttir, 2019 | Motivational interviewing (Intervention cohort) vs. routine care (historical reference cohort) | More than 1 episode of hypoglycemia in past week at late pregnancy visit | 189 | OR (95% CI): 3.19 (1.63 to 6.27)  P= 0.0007 |  |
| ***Weight-related*** | | | | | |
| Ásbjörnsdóttir, 2019 | Motivational interviewing (Intervention cohort) vs. routine care (historical reference cohort) | Weight at late pregnancy visit (Kg) | 189 | Mean (SD) (Kg weight):  Motivational interviewing: 101.3 (21.3)  Routine care: 100.3 (23.8)  P=0.78 |  |
| Ásbjörnsdóttir, 2019 | Motivational interviewing (Intervention cohort) vs. routine care (historical reference cohort) | Total gestational weight gain (Kg) | 189 | Mean (SD) (Kg weight):  Motivational interviewing: 9.2 (5.8)  Routine care: 10.2 (5.8)  P=0.25 |  |
| Ásbjörnsdóttir, 2019 | Motivational interviewing (Intervention cohort) vs. routine care (historical reference cohort) | Total gestational weight gain per week (Kg) | 189 | Mean (SD) (Kg weight):  Motivational interviewing: 0.26 (0.16)  Routine care: 0.28 (0.16)  P=0.31 |  |
| Ásbjörnsdóttir, 2019 | Motivational interviewing (Intervention cohort) vs. routine care (historical reference cohort) | Gestational weight gain according to IOM recommendations: insufficient | 189 | OR (95% CI): 1.21 (0.64-2.28)  P=0.56 |  |
| Ásbjörnsdóttir, 2019 | Motivational interviewing (Intervention cohort) vs. routine care (historical reference cohort) | Gestational weight gain according to IOM recommendations: appropriate | 189 | OR (95% CI): 1.51 (0.80-2.85)  P=0.20 |  |
| Ásbjörnsdóttir, 2019 | Motivational interviewing (Intervention cohort) vs. routine care (historical reference cohort) | Gestational weight gain according to IOM recommendations: excessive | 189 | OR (95% CI): 0.69 (0.38-1.26)  P=0.23 |  |
| Ásbjörnsdóttir, 2019 | Motivational interviewing (Intervention cohort) vs. routine care (historical reference cohort) | Weight loss during pregnancy | 189 | OR (95% CI): 1.20 (0.31-4.60)  P=0.79 |  |
| ***Outcome - NICU admission*** | | | | | |
| Stafl, 2023 | One or more missed appointments vs. no missed appointments | NICU admission | 902 | aOR (95% CI): 1.00 (0.72-1.40) | Maternal age, smoking, Pampalon Maternal Deprivation Index and pre-pregnancy weight ≥ 91 kg |
| Perichart-Perera, 2009 | Medical Nutrition Therapy (MNT) programme: Individual nutrition counselling with an intensive education component vs. routine care (historical reference cohort) | NICU admission | 96 (n=47 control, n=49 MNT) | OR (95% CI): 0.21 (0.03-0.51)  P<0.001 |  |
| ***Outcome - Neonatal growth*** | | | | | |
| Ásbjörnsdóttir, 2019 | Motivational interviewing (Intervention cohort) vs. routine care (historical reference cohort) | Birthweight (g) | 189 | Mean (SD) (birthweight):  Motivational interviewing: 3167 (659)  Routine care: 3324 (636)  P=0.1 |  |
| Ásbjörnsdóttir, 2019 | Motivational interviewing (Intervention cohort) vs. routine care (historical reference cohort) | Birthweight (z score) | 189 | Mean (SD) (birthweight):  Motivational interviewing: 0.24 (1.36)  Routine care: 0.61 (1.38)  P=0.06 |  |
| Ásbjörnsdóttir, 2019 | Motivational interviewing (Intervention cohort) vs. routine care (historical reference cohort) | Birth length (cm) | 189 | Mean (SD) (birth length):  Motivational interviewing: 50.4 (3)  Routine care: 50.5 (2)  P=0.85 |  |
| Ásbjörnsdóttir, 2019 | Motivational interviewing (Intervention cohort) vs. routine care (historical reference cohort) | Abdominal circumference (cm) | 189 | Mean (SD) (abdominal circumference):  Motivational interviewing: 32.2 (2)  Routine care: 32.8 (2)  P=0.15 |  |
| Ásbjörnsdóttir, 2019 | Motivational interviewing (Intervention cohort) vs. routine care (historical reference cohort) | Head circumference (cm) | 189 | Mean (SD) (head circumference):  Motivational interviewing: 34.4 (2)  Routine care: 34.4 (2)  P=0.9 |  |
| ***Outcome - Insulin use*** | | | | | |
| Ásbjörnsdóttir, 2019 | Motivational interviewing (Intervention cohort) vs. routine care (historical reference cohort) | Insulin treatment at late pregnancy visit | 189 | OR (95% CI): 2.17 (0.87-5.39)  P=0.096 |  |
| Ásbjörnsdóttir, 2019 | Motivational interviewing (Intervention cohort) vs. routine care (historical reference cohort) | Insulin dose at late pregnancy visit | 189 | Median (IQR) (insulin IU/Kg):  Motivational interviewing: 0.95 (0.66, 1.51)  Routine care: 0.74 (0.40, 1.28)  P=0.03 |  |
| ***Perinatal/infant health*** | | | | | |
| Ásbjörnsdóttir, 2019 | Motivational interviewing (Intervention cohort) vs. routine care (historical reference cohort) | Perinatal morbidity (major congenital malformation, neonatal hypoglycemia, jaundice and/or transient tachypnea) | 189 | OR (95% CI): 0.80 (0.44-1.45)  P=0.46 |  |
| Ásbjörnsdóttir, 2019 | Motivational interviewing (Intervention cohort) vs. routine care (historical reference cohort) | Major congenital malformations | 189 | OR (95% CI): 0.62 (0.10-3.83)  P=0.61 |  |
| Ásbjörnsdóttir, 2019 | Motivational interviewing (Intervention cohort) vs. routine care (historical reference cohort) | Jaundice | 189 | OR (95% CI): 1.50 (0.63-3.53)  P=0.35 |  |
| Ásbjörnsdóttir, 2019 | Motivational interviewing (Intervention cohort) vs. routine care (historical reference cohort) | Transient tachypnea | 189 | OR (95% CI): 0.47 (0.21-1.09)  P=0.077 |  |
| ***Outcome - Gestational Age*** | | | | | |
| Ásbjörnsdóttir, 2019 | Motivational interviewing (Intervention cohort) vs. routine care (historical reference cohort) | Gestational age at delivery (days) | 189 | Median (IQR) (gestational age):  Motivational interviewing: 263 (260, 270)  Routine care: 266 (259, 268)  P=0.98 |  |

Abbreviations: CI (Confidence Interval); HBA1c (haemoglobin A1c, a blood test that measures the average blood sugar level over the past two to three months); IOM (Institute of Medicine); IQR (Inter-quartile Range); LGA (Large for Gestational Age); MNT (Medical Nutrition Therapy); NICU (Neonatal Intensive Care Unit); OR (Odds Ratio); SD (Standard Deviation); SDSCA (Summary of Diabetes Self-Care Activities); SGA (Small for Gestational Age).

**Supplementary Table 11**. Findings on associations between planned c-section and maternal and child health outcomes

| **First author, year** | **Exposure description** | **Pregnancy outcome** | **Total sample size** | **Result** | **Confounder adjustments** |
| --- | --- | --- | --- | --- | --- |
| Alrais, 2021 | Planned C-section: Attempted Vaginal delivery vs Planned C-section | *Neonatal hypoglycemia | 215 | aOR (95% CI): 1.05 (0.75-1.45) | Nulliparous, gestational age at delivery, maternal blood glucose before delivery |
| Alrais, 2021 | Planned C-section: Attempted Vaginal delivery vs Planned C-section | **Composite neonatal outcome | 215 | aOR (95% CI): 1.37 (1.07-1.75) | Nulliparous, gestational age at delivery, maternal blood glucose before delivery |
| Alrais, 2021 | Planned C-section: Attempted Vaginal delivery vs Planned C-section | Should dystocia | 215 | aOR (95% CI): 0.0683 (0.037-1.250) | Nulliparous, gestational age at delivery, maternal blood glucose before delivery |
| Alrais, 2021 | Planned C-section: Attempted Vaginal delivery vs Planned C-section | LGA | 215 | OR (95% CI): 1.76 (0.93-3.36) |  |
| Alrais, 2021 | Planned C-section: Attempted Vaginal delivery vs Planned C-section | NICU admission | 215 | OR (95% CI): 1.69 (0.98-2.91) |  |
| Alrais, 2021 | Planned C-section: Attempted Vaginal delivery vs Planned C-section | Respiratory distress syndrome | 215 | OR (95% CI): 6.13 (1.76-21.32) |  |
| Alrais, 2021 | Planned C-section: Attempted Vaginal delivery vs Planned C-section | 5 min APGAR <5 | 215 | aOR (95% CI): 10.84 (0.6030-194.9592) | Nulliparous, gestational age at delivery, maternal blood glucose before delivery |

Abbreviations: APGAR (refers to the scoring system used by medical professionals to assess the health of a newborn baby shortly after birth and stands for Appearance, Pulse, Grimace, Activity, and Respiration); CI (Confidence Interval); C-Section (Caesarean Section); LGA (Large for Gestational Age); NICU (Neonatal Intensive Care Unit); OR (Odds Ratio).

* Neonatal Hypoglycemia: blood glucose <40mg/dl <24h of age, or <50ml/dl >24 hour of age

** Composite neonatal outcome: including one of: shoulder dystocia, large for gestational age, NICU admission, or respiratory distress syndrome

**Supplementary Table 12**. Findings on associations between maternal BMI and maternal and child health outcomes

| **First author, year** | **Exposure description** | **Pregnancy outcome** | **Total sample size** | **Result** | **Confounder adjustments** |
| --- | --- | --- | --- | --- | --- |
| ***Outcome - Gestational weight gain*** | | | | | |
| Colatrella, 2009 | Preconception BMI: overweight vs. normal weight | Gestational weight gain (kg) | 49 | Mean (SD) (kg GWG):  Overweight: 7.3 (6.6)  Normal weight: 10.1 (3.3)  P=NS |  |
| Nørgaard, 2016 | Preconception BMI: overweight vs. normal weight | Gestational weight gain (kg) | 39 | Mean (SD) (kg GWG):  Overweight: 11.7 (7.2)  Normal weight: 13.4 (6.4)  P=NR |  |
| Colatrella, 2009 | Preconception BMI: obesity vs. normal weight | Gestational weight gain (kg) | 50 | Mean (SD) (kg GWG):  Obesity: 7.1 (5.2)  Normal weight: 10.1 (3.3)  P=NS |  |
| Nørgaard, 2016 | Preconception BMI: obesity vs. normal weight | Gestational weight gain (kg) | 82 | Mean (SD) (kg GWG):  Obesity: 11.5 (6.8)  Normal weight: 13.4 (6.4)  P=NR |  |
| Nørgaard, 2016 | Preconception BMI: overweight vs. normal weight | Excessive gestational weight gain | 39 | OR (95% CI): 2.75 (0.68-11.11) |  |
| Nørgaard, 2016 | Preconception BMI: obesity vs. normal weight | Excessive gestational weight gain | 82 | OR (95% CI): 4.92 (1.43-16.94) |  |
| ***Outcome - Pre-eclampsia*** | | | | | |
| Colatrella, 2009 | Preconception BMI: overweight vs. normal weight | Pre-eclampsia | 49 | OR (95% CI): 0.88 (0.05-14.92) |  |
| Persson, 2016 | BMI: overweight vs. normal weight | Pre-eclampsia | 886 | aOR (95% CI): 0.84 (0.33-2.16) | Maternal BMI, height, age, parity, education, smoking, country of birth |
| Oppermann, 2020 | BMI: overweight/obesity vs. underweight/normal weight | Pre-eclampsia | 119 | OR (95% CI): 0.51 (0.08-3.22) |  |
| Colatrella, 2009 | Preconception BMI: obesity vs. normal weight | Pre-eclampsia | 50 | OR (95% CI): 0.85 (0.05-14.33) |  |
| Persson, 2016 | BMI: obesity vs. normal weight | Pre-eclampsia | 886 | aOR (95% CI): 1.23 (0.56-2.72 | Maternal BMI, height, age, parity, education, smoking, country of birth. |
| Kapustin, 2023 | BMI: obesity vs. no obesity | Pre-eclampsia | 214 | RR (95% CI): 2.25 (1.20-4.22) |  |
| ***Outcome - Hypertension*** | | | | | |
| Colatrella, 2009 | Preconception BMI (kg/m2) | Hypertension in pregnancy | 76 | aOR (95% CI): 5.25 (1.3-21.6) | Weight increase, age, parity, smoking and 1st, 2nd and 3rd trimester HbA1c. |
| Colatrella, 2009 | Preconception BMI: overweight vs. normal weight | Hypertension overall | 76 | OR (95% CI): 2.9 (0.7 - 12.9) | Weight increase, age, parity, smoking and 1st, 2nd and 3rd trimester HbA1c. |
| Colatrella, 2009 | Preconception BMI: obesity vs. normal weight | Hypertension overall | 76 | OR (95% CI): 9.7 (2.3-40.8) | Weight increase, age, parity, smoking and 1st, 2nd and 3rd trimester HbA1c. |
| Colatrella, 2009 | Preconception BMI: overweight vs. normal weight | Hypertension in pregnancy | 49 | OR (95% CI): 2.96 (0.68-12.91) | Weight increase, age, parity, smoking and 1st, 2nd and 3rd trimester HbA1c. |
| Colatrella, 2009 | Preconception BMI: obesity vs. normal weight | Hypertension in pregnancy | 50 | OR (95% CI): 9. 70 (2.31-40.76) | Weight increase, age, parity, smoking and 1st, 2nd and 3rd trimester HbA1c. |
| Colatrella, 2009 | Preconception BMI: overweight vs. normal weight | Chronic Hypertension | 49 | OR (95% CI): 5.24 (0.56-48.65) | Weight increase, age, parity, smoking and 1st, 2nd and 3rd trimester HbA1c. |
| Colatrella, 2009 | Preconception BMI: obesity vs. normal weight | Chronic hypertension | 50 | OR (95% CI): 7.70 (0.87-68.19) | Weight increase, age, parity, smoking and 1st, 2nd and 3rd trimester HbA1c. |
| Colatrella, 2009 | Preconception BMI: overweight vs. normal weight | Pregnancy-induced hypertension | 49 | OR (95% CI): 1.83 (0.15-21.66) | Weight increase, age, parity, smoking and 1st, 2nd and 3rd trimester HbA1c. |
| Colatrella, 2009 | Preconception BMI: obesity vs. normal weight | Pregnancy-induced hypertension | 50 | OR (95% CI): 9.26 (1.06-80.94) | Weight increase, age, parity, smoking and 1st, 2nd and 3rd trimester HbA1c. |
| ***Outcome - Birthweight*** | | | | | |
| Colatrella, 2009 | Preconception BMI: overweight vs. normal weight | Birth weight (g) | 49 | Mean (SD) (birthweight):  Overweight: 2919 (801)  Normal weight: 2870 (480)  P=not significant |  |
| Nørgaard, 2016 | Preconception BMI: overweight vs. normal weight | Birth weight (g) | 39 | Mean (SD) (birthweight):  Overweight: 3,221 ± 567  Normal weight: 3,113 ± 767  P=NR |  |
| Nørgaard, 2016 | Preconception BMI: overweight vs. normal weight | Birth weight (SD-score) | 39 | Mean (SD) (birthweight):  Overweight: 0.25 (1.2)  Normal weight: -0.09 (1.5)  P=NR |  |
| Colatrella, 2009 | Preconception BMI: obesity vs. normal weight | Birth weight (g) | 50 | Mean (SD) (birthweight):  Obesity: 3525 (455)  Normal weight: 2870 (480)  P=0.01 |  |
| Nørgaard, 2016 | Preconception BMI: obesity vs. normal weight | Birth weight (g) | 82 | Mean (SD) (birthweight):  Obesity: 3,296 (839)  Normal weight: 3,113 (767)  P=NR |  |
| Nørgaard, 2016 | Preconception BMI: obesity vs. normal weight | Birth weight (SD-score) | 82 | Mean (SD) (birthweight):  Obesity: 0.63 (1.8)  Normal weight: -0.09 (1.5)  P=NR |  |
| ***Outcome - High birth weight*** | | | | | |
| Colatrella, 2009 | Preconception BMI: overweight vs. normal weight | Macrosomia >4kg | 49 | OR (95% CI): 14.90 (0.79 to 28.01) |  |
| Nørgaard, 2016 | Preconception BMI: overweight vs. normal weight | LGA | 39 | aOR (95% CI): 3.06 (0.54-17.23) | Gender and gestational age. |
| Colatrella, 2009 | Preconception BMI: obesity vs. normal weight | Macrosomia >4kg | 50 | OR (95% CI): 20.48 (1.11-377.86) |  |
| Nørgaard, 2016 | Preconception BMI: obesity vs. normal weight | LGA | 82 | OR (95% CI): 3.04 (0.63-14.66) | Gender and gestational age. |
| Ladfors, 2017 | Early pregnancy BMI (kg/m^2^) | LGA >2 SD of mean | 87 | OR (95% CI): 1.09 (1.00-1.19) |  |
| Alexander, 2019 | Preconception BMI (kg/m^2^) | LGA (birth weight and sex corrected) | 253 | aOR (95% CI): 1.05 (0.99-1.11) | Ethnicity, parity, insulin, hypertension, preconception BMI, first trimester A1C, delivery A1C. |
| Rowan, 2009 | BMI at booking (kg/m^2^) | LGA | 180 | Mean (SD) (BMI):  No LGA: 33.9 (7.0)  LGA: 36.4 (7.6)  P=NR |  |
| Olmos.2009 | Preconception BMI (kg/m^2^) | LGA (newborn weight centile ≥90) | 51 | Mean (SD) (BMI):  No LGA: 28.85 (1.02)  LGA: 32.15 (1.56)  P=0.032 |  |
| Olmos, 2009 | Admission BMI (kg/m^2^) | LGA (newborn weight centile ≥90) | 51 | Mean (SD) (BMI):  No LGA: 30.69 (1.05)  LGA: 32.65 (1.03)  P=0.04 |  |
| Olmos, 2009 | Antepartum BMI (kg/m^2^) | LGA (newborn weight centile ≥90) | 51 | Mean (SD) (BMI):  No LGA: 32.08 (1.122)  LGA: 34.7 (1.13)  P=0.111 |  |
| Rowan, 2009 | Weight at booking (kg) | LGA | 180 | Mean (SD) (weight):  No LGA: 90.5 (21)  LGA: 99.7 (21.3)  P=NR |  |
| Ladfors, 2017 | Weight in early pregnancy (kg) | LGA >2 SD of mean | 87 | OR (95% CI): 1.04 (1.01-1.07) |  |
| ***Outcome - Low birth weight*** | | | | | |
| Rowan, 2009 | Weight at booking (kg) | SGA | 159 | Mean (SD) (weight):  No SGA: 90.5 (21)  SGA: 89.2 ± 22.9  P=NR |  |
| Rowan, 2009 | BMI at booking (kg/m2) | SGA | 159 | Mean (SD) (BMI):  No SGA: 33.9 (7.0)  SGA: 32.6 (6.7)  P=NR |  |
| Feig, 2022 | Preconception BMI (kg/m2) | SGA | 460 | Mean (SD) (BMI):  No SGA: 33.9 (7.4)  SGA: 32.4 (6.4)  P=0.2 |  |
| Nørgaard, 2016 | Preconception BMI: overweight vs. normal weight | SGA | 39 | aOR (95% CI): 0.41 (0.06-2.81) | Gender and gestational age. |
| Nørgaard, 2016 | Preconception BMI: obesity vs. normal weight | SGA | 82 | aOR (95% CI): 0.5977 (0.13-2.56) | Gender and gestational age. |
| ***Outcome - Gestational age*** | | | | | |
| Nørgaard, 2016 | Preconception BMI: overweight vs. normal weight | Preterm delivery <37 weeks | 39 | OR (95% CI): 1.05 (0.16-7.13) |  |
| Nørgaard, 2016 | Preconception BMI: obesity vs. normal weight | Preterm delivery <37 weeks | 82 | OR (95% CI): 1.72 (0.35-8.51) |  |
| Søholm, 2021 | Preconception BMI (kg/m2) | Gest age at delivery (preterm <37/40) | 86 | Mean (SD) (BMI):  No preterm: 32.9 (7)  Preterm: 32 (4)  P=0.77 | Gender and gestational age. |
| Nørgaard, 2016 | Preconception BMI: overweight vs. normal weight | Gestational age at delivery (days) | 39 | Mean (SD) (days):  Overweight: 264 (10)  Normal weight: 264 (16)  p=NR |  |
| Nørgaard, 2016 | Preconception BMI: obesity vs. normal weight | Gestational age at delivery (days) | 82 | Mean (SD) (days):  Overweight: 261 (18)  Normal weight: 264 (16)  p=NR |  |
| ***Outcome - Glycemia*** | | | | | |
| Murphy, Bell, 2017 | BMI at booking (kg/m2) | Achieving HbA1c <48 mmol/l target | 1386 | Mean (BMI):  Not in target: 34.0  In target: 31.9  p=<0.001 |  |
| Murphy, Howgate, 2021 | BMI: underweight vs. normal weight | attainment of target glycaemic control | 635 | aOR (95% CI): 0.86 (0.36−2.10) | Race or ethnicity, deprivation quintile, diabetes duration. |
| Murphy, Howgate, 2021 | BMI: overweight vs. normal weight | attainment of target glycaemic control | 2130 | aOR (95% CI): 0.80 (0.66−0.97) | Race or ethnicity, deprivation quintile, diabetes duration. |
| Murphy, Howgate, 2021 | BMI: obesity (30.0-34.9 kg/m2) vs. normal weight | attainment of target glycaemic control | 2320 | aOR (95% CI): 0.65 (0.54−0.79) | Race or ethnicity, deprivation quintile, diabetes duration. |
| Murphy, Howgate, 2021 | BMI: obesity (35.0-39.9 kg/m2) vs. normal weight | attainment of target glycaemic control | 1840 | aOR (95% CI): 0.55 (0.45−0.68) | Race or ethnicity, deprivation quintile, diabetes duration. |
| Murphy, Howgate, 2021 | BMI: obesity (≥40.0 kg/m2) vs. normal weight | attainment of target glycaemic control | 1720 | aOR (95% CI): 0.52 (0.42−0.64) | Race or ethnicity, deprivation quintile, diabetes duration. |
| Nørgaard, 2016 | Preconception BMI: overweight vs. normal weight | HbA1c 1st visit (%, mmol/mol) | 39 | Mean (SD) (%, mmol/mol):  Overweight: 6.5 (1.5)  Normal weight: 6.5 (1.4)  p=NR |  |
| Nørgaard, 2016 | Preconception BMI: obesity vs. normal weight | HbA1c 1st visit (%, mmol/mol) | 82 | Mean (SD) (%, mmol/mol):  Obesity: 6.7 (1.1)  Normal weight: 6.5 (1.4)  p=NR |  |
| Nørgaard, 2016 | Preconception BMI: overweight vs. normal weight | HbA1c last visit (%, mmol/mol) | 39 | Mean (SD) (%, mmol/mol):  Overweight: 6.0 (0.6)  Normal weight: 6.0 (0.8)  p=NR |  |
| Nørgaard, 2016 | Preconception BMI: obesity vs. normal weight | HbA1c last visit (%, mmol/mol) | 82 | Mean (SD) (%, mmol/mol):  Obesity: 6.0 (0.7)  Normal weight: 6.0 (0.8)  p=NR |  |
| ***Outcome - Infant health*** | | | | | |
| Endo, 2018 | Preconception BMI (kg/m2) | Perinatal complications | 11 | Mean (SD) BMI):  Complications: 31.1 (3.3)  No complications: 23.2 (1.0)  p=0.001 |  |
| Rais, 2019 | BMI (kg/m2) | LGA placenta (vs appropriate for gestational age (AGA) placenta) | 136 | Mean (SD) BMI):  LGA placenta: 38.1 (9.2)  AGA placenta: 37.2 (7.7)  p=0.58 |  |
| Roland, 2005 | BMI (kg/m2) | Congenital anomaly | 146 | OR (95% CI): 1.09 (1.01-1.18) |  |
| Colatrella, 2009 | Preconception BMI: overweight vs. normal weight | Congenital malformation | 49 | OR (95% CI): 0.87 (0.11-6.76) |  |
| Colatrella, 2009 | Preconception BMI: obesity vs. normal weight | Congenital malformation | 50 | OR (95% CI): 0.84 (0.10-6.48) |  |
| Kapustin, 2023 | BMI: obesity vs no obesity | Neonatal hypoglycemia | 214 | RR (95% CI): 3.37 (1.27-8.99) |  |
| Kapustin, 2023 | BMI: obesity vs no obesity | Excessive fetal adiposity | 214 | RR (95% CI): 1.83 (1.07-2.12) |  |
| Nørgaard, 2016 | Preconception BMI: overweight vs. normal weight | Neonatal jaundice | 39 | OR (95% CI): 1.42 (0.11-17.23) |  |
| Nørgaard, 2016 | Preconception BMI: obesity vs. normal weight | Neonatal jaundice | 82 | OR (95% CI): 1.77 (0.20-15.59) |  |
| ***Outcome - Treatment and care-related interventions*** | | | | | |
| Nørgaard, 2016 | Preconception BMI: overweight vs. normal weight | Insulin dose, first visit (IU/kg/24-h | 39 | Mean (SD) (IU/kg/24-h):  Overweight: 0.31 (0.4)  Normal weight: 0.21 (0.3)  p=NR |  |
| Nørgaard, 2016 | Preconception BMI: obesity vs. normal weight | Insulin dose, first visit (IU/kg/24-h | 82 | Mean (SD) (IU/kg/24-h):  Obesity: 0.26 (0.3)  Normal weight: 0.21 (0.3)  p=NR |  |
| Nørgaard, 2016 | Preconception BMI: overweight vs. normal weight | Insulin dose, last visit (IU/kg/24-h) | 39 | Mean (SD) (IU/kg/24-h):  Overweight: 0.86 (0.7)  Normal weight: 1.0 (0.8)  p=NR |  |
| Nørgaard, 2016 | Preconception BMI: obesity vs. normal weight | Insulin dose, last visit (IU/kg/24-h) | 82 | Mean (SD) (IU/kg/24-h):  Obesity: 1.0 (0.7)  Normal weight: 1.0 (0.8)  p=NR |  |
| Nadeau, 2021 | BMI at 1^st^ prenatal visit (kg/m2) | Insulin dose ≥2 units/kg at delivery | 160 | Mean (SD) (BMI):  Insulin dose ≥2 units/kg: 38.6 (9.2)  Insulin dose <2 units/kg: 36.6 (8.2)  p=0.15 |  |
| Abell, 2017 | BMI: overweight vs. normal weight | Special care nursery admission | 57 | OR (95% CI): 6.14 (1.19-31.81) |  |
| Abell, 2017 | BMI: obesity vs. normal weight | Special care nursery admission | 107 | OR (95% CI): 3.69 (1.05-12.99) |  |
| Isabey, 2021 | BMI (kg/m2) | Caesarean section | 192 | Mean (SD) (BMI):  Caesarean section: 39.3 (6.6)  Spontaneous vaginal delivery: 36.1 (7.3)  p=NR |  |
| Nørgaard, 2016 | Preconception BMI: overweight vs. normal weight | Caesarean section | 39 | OR (95% CI): 0.68 (0.18-2.53) |  |
| Nørgaard, 2016 | Preconception BMI: obesity vs. normal weight | Caesarean section | 82 | OR (95% CI): 1.21 (0.40-3.63) |  |
| Stafl, 2023 | Preconception weight ≥91 kg vs. <91kg | Missed 1 or more antenatal appointments | 902 | aOR (95% CI): 1.53 (1.16-2.03) | Maternal age, smoking, Deprivation Index. |
| Rasmussen, 2010 | Preconception BMI (kg/m2) | Progression of retinopathy | 80 | Mean (SD) (BMI):  Progression: 30.7 (4.6)  No progression: 32.4 (7.3)  p=0.31 |  |

Abbreviations: AGA (Appropriate for Gestational Age); BMI (Body Mass Index); CI (Confidence Interval) GWG (Gestational Weight Gain); HBA1c (haemoglobin A1c, a blood test that measures the average blood sugar level over the past two to three months); LGA (Large for Gestational Age); NR (Not Reported); OR (Odds Ratio); RR (Relative Risk); SD (Standard Deviation); SGA (Small for Gestational Age).

BMI, body mass index; CI, confidence interval; GWG, gestational weight gain; LGA, large for gestational age; nr, not reported; ns, not significant; OR, odds ratio; RR, relative risk; SD, standard deviation; SGA, small for gestational age.

Normal weight defined as 18.5-24.9kg/m^2^, overweight as 25.0-29.9 kg/m^2^ and obesity as ≥30 kg/m^2^ unless specified otherwise.

**Supplementary Table 13**. Findings on associations between gestational weight gain and maternal and child health outcomes

| **First author, year** | **Exposure description** | **Pregnancy outcome** | **Total sample size** | **Result** | **Confounder adjustments** |
| --- | --- | --- | --- | --- | --- |
| ***Outcome - LGA/ macrosomia*** | | | | | |
| Yee, 2011 | Weight change less than IOM guidelines vs. weight change within IOM guidelines | LGA >90th percentile | 212 | aOR (95% CI): 0.85 (0.49-1.48) | Age, race/ethnicity, parity, education. |
| Yee, 2011 | Weight change less than IOM guidelines vs. weight change within IOM guidelines | Birthweight >4000g | 147 | aOR (95% CI): 1.28 (0.66-2.48) | Age, race/ethnicity, parity, education. |
| Parellada,2014 | Excessive GWG vs. no excessive GWG | Macrosomia >4kg | 142 | OR (95% CI): 14.04 (3.08-63.88) |  |
| Yee, 2011 | Weight change less than IOM guidelines vs. weight change within IOM guidelines | Birthweight >4000g | 314 | aOR (95% CI): 2.59 (1.56-4.30) | Age, race/ethnicity, parity, education. |
| Yee  (meta-analysis), 2011 | Weight change less than IOM guidelines vs. weight change within IOM guidelines | LGA >90th percentile | 442 | aOR (95% CI): 2.00 (1.33-3.00) | Age, race/ethnicity, parity, education. |
| Parellada  (meta-analysis), 2014 | Excessive GWG vs. no excessive GWG | LGA >90th centile | 142 | OR (95% CI): 3.68 (1.75-7.73) |  |
| Ladfors  (meta-analysis), 2017 | Excessive GWG vs. no excessive GWG | LGA >2 SD of mean | 87 | OR (95% CI): 3.52 (1.25-9.93) |  |
| Alexander  (meta-analysis), 2019 | Excessive GWG vs. no excessive GWG | LGA (birthweight and sex corrected) | 253 | aOR (95% CI): 2.26 (0.85-5.97) | Ethnicity, parity, insulin, hypertension, preconception BMI, first trimester A1C, delivery A1C. |
| Alexander, 2019 | Excessive GWG vs. no excessive GWG | LGA (GROW) | 253 | aOR (95% CI): 1.04 (0.91-1.18) | Ethnicity, parity, insulin, hypertension, preconception BMI, first trimester A1C, delivery A1C. |
| Olmos, 2009 | Weight change per week (kg) | Macrosomia (>90th percentile) | 51 | Mean (kg):  Macrosomia: 0.233  No macrosomia: 0.21  P=0.752 |  |
| Olmos, 2009 | Change in BMI per week | Macrosomia (>90th percentile) | 51 | Mean (BMI):  Macrosomia: 0.187  No macrosomia: 0.147  P=0.066 |  |
| Olmos, 2009 | Pregnancy weight gain (kg) | Macrosomia (>90th percentile) | 51 | Mean (kg):  Macrosomia: 8.59  No macrosomia: 8.54  P=0.985 |  |
| Ladfors, 2017 | Gestational weight gain (kg) | LGA birth weight >2 SD of mean | 87 | OR (95% CI): 1.13 (1.03-1.24) |  |
| Ladfors, 2017 | Gestational weight gain (kg) | LGA birth weight >2 SD of mean | 87 | aOR (95% CI): 1.18 (1.05-1.32) | Smoking, age, parity, BMI. |
| Asbjornsdottir, 2013 | Maternal weight gain >5kgs vs. weight gain ≤5kgs | LGA (>90th percentile) | 58 | OR (95% CI): 4.8 (0.97-23.85) |  |
| Asbjornsdottir, 2013 | Maternal weight gain >5kgs vs. weight gain ≤5kgs | Macrosomia (birth weight >4000g) | 58 | OR (95% CI): 8.88 (0.48-163.05) |  |
| ***Outcome - SGA*** | | | | | |
| Yee, 2011 | Weight change less than IOM guidelines vs. weight change within IOM guidelines | SGA <10th percentile | 58 | aOR (95% CI): 1.95 (0.80-4.74) | Age, race/ethnicity, parity, education. |
| Feig. 2022 | Weight change less than IOM guidelines vs. weight change within or above IOM guidelines | SGA | 451 | OR (95% CI): 1.60 (0.79-3.26) |  |
| Parellada  (meta-analysis), 2014 | Excessive GWG vs. no excessive GWG | SGA <10th centile | 142 | OR (95% CI): 0.56 (0.18-1.73) |  |
| Yee  (meta-analysis), 2011 | Weight change less than IOM guidelines vs. weight change within IOM guidelines | SGA <10th percentile | 64 | aOR (95% CI): 0.94 (0.43-2.07) | Age, race/ethnicity, parity, education. |
| Feig  (meta-analysis), 2022 | Weight change less than IOM guidelines vs. weight change within or above IOM guidelines | SGA | 451 | OR (95% CI): 0.72 (0.38-1.35) |  |
| Asbjornsdottir, 2013 | Maternal weight gain >5kgs vs. weight gain ≤5kgs | SGA (<10th percentile) | 58 | OR (95% CI): 0.50 (0.10-2.54) |  |
| ***Outcome - Birthweight*** | | | | | |
| Parellada, 2014 | Excessive GWG vs. no excessive GWG | Birthweight (g) | 142 | Median (range) (g):  Excessive GWG: 3712 (1070-4816)  No excessive GWG: 3258 (720-4558)  P=0.001 |  |
| Asbjornsdottir, 2013 | Maternal weight gain >5kgs vs. weight gain ≤5kgs | Birth weight (g) | 58 | Median (range) (g):  Excessive GWG: 3364 (1070 - 4432)  No excessive GWG: 3134 (1278 - 3870)  P=0.12 |  |
| Parellada, 2014 | Total GWG (kg) | Birthweight z score | 142 | β coefficient (95% CI): 0.1 (0.06 to 0.14) increase in infant birth weight z score for each kg increase in total GWG | Preconception BMI, smoking, HbA1c, insulin dose at last visit, ethnicity, parity. |
| ***Outcome - Pre-eclampsia*** | | | | | |
| Parellada, 2014 | Excessive GWG vs. no excessive GWG | Pre-eclampsia | 142 | OR (95% CI): 2.77 (0.49-15.65) |  |
| Oppermann. 2020 | Excessive GWG vs. no excessive GWG | Pre-eclampsia | 117 | OR (95% CI): 1.17 (0.50-2.75) |  |
| Asbjornsdottir, 2013 | Maternal weight gain >5kgs vs. weight gain ≤5kgs | Pre-eclampsia | 58 | OR (95% CI): 4.20 (0.21-82.39) |  |
| Oppermann, 2020 | Total GWG (kg) | Pre-eclampsia | 113 | Mean (SD) (kg):  Pre-eclampsia: 9.8 (4.7)  No pre-eclampsia: 7.8 (7.0)  P=0.151 |  |
| ***Outcome - Caesarean section*** | | | | | |
| Parellada, 2014 | Excessive GWG vs. no excessive GWG | Emergency caesarean | 142 | OR (95% CI): 2.38 (0.95-5.94) |  |
| Parellada, 2014 | Excessive GWG vs. no excessive GWG | Elective caesarean | 142 | OR (95% CI): 0.91 (0.43-1.91) |  |
| Yee, 2011 | Weight change less than IOM guidelines vs. weight change within IOM guidelines | All caesarean | 762 | aOR (95% CI): 1.47 (1.03-2.10) | Age, race/ethnicity, parity, education. |
| Yee, 2011 | Weight change greater than IOM guidelines vs. weight change within IOM guidelines | Primary caesarean | 370 | aOR (95% CI): 1.62 (1.03-2.57) | Age, race/ethnicity, parity, education. |
| Yee, 2011 | Weight change greater than IOM guidelines vs. weight change within IOM guidelines | All caesarean | 474 | aOR (95% CI): 1.10 (0.69-1.74) | Age, race/ethnicity, parity, education. |
| Yee, 2011 | Weight change less than IOM guidelines vs. weight change within IOM guidelines | Primary caesarean | 217 | aOR (95% CI): 1.49 (0.83-2.68) | Age, race/ethnicity, parity, education. |
| Asbjornsdottir, 2013 | Maternal weight gain >5kgs vs. weight gain ≤5kgs | Emergency caesarean | 58 | OR (95% CI): 5.86 (0.69-49.62) |  |
| Asbjornsdottir, 2013 | Maternal weight gain >5kgs vs. weight gain ≤5kgs | Elective caesarean | 58 | OR (95% CI): 0.95 (0.29-3.11) |  |
| ***Outcome - Preterm delivery / gestational age*** | | | | | |
| Parellada, 2014 | Excessive GWG vs. no excessive GWG | Preterm delivery | 142 | OR (95% CI): 1.07 (0.39-2.90) |  |
| Søholm, 2021 | Excessive GWG vs. no excessive GWG | Preterm delivery (<37 weeks gestation) | 86 | OR (95% CI): 6.09 (1.78-20.75) |  |
| Parellada, 2014 | Excessive GWG vs. no excessive GWG | Gestational age at delivery (days) | 142 | Median (range) (days)  No excessive GWG: 266 (184-284)  Excessive GWG: 265 (206-280)  P=0.81 |  |
| Søholm, 2021 | GWG (grams/week) | Preterm delivery (<37 weeks gestation) | 86 | Mean (SD) (grams/week)  No preterm delivery: 265 (179)  Preterm delivery: 424 (219)  P=0.003 |  |
| Asbjornsdottir, 2013 | Maternal weight gain >5kgs vs. weight gain ≤5kgs | Gestational age at delivery (days) | 58 | Median (range) (days)  No excessive GWG: 268 (221-284)  Excessive GWG: 262 (206-280)  P=0.039 |  |
| Asbjornsdottir, 2013 | Maternal weight gain >5kgs vs. weight gain ≤5kgs | Preterm delivery | 58 | OR (95% CI): 2.75 (0.53-14.02) |  |
| ***Outcome - Growth*** | | | | | |
| Parellada, 2014 | Excessive GWG vs. no excessive GWG | Length at birth cm | 142 | Median (range):  No excessive GWG: 50 (32-56)  Excessive GWG: 52(46-57)  P=0.262 |  |
| Parellada, 2014 | Excessive GWG vs. no excessive GWG | Ponderal index | 142 | Median (range):  No excessive GWG: 25.2 (19.3-32.4)  Excessive GWG: 25.9 (20.1-32)  P=0.071 |  |
| Asbjornsdottir, 2013 | Maternal weight gain >5kgs vs. weight gain ≤5kgs | Ponderal Index (kg/m3) | 58 | Median (range):  No excessive GWG: 23.9 (20.4 - 29.2)  Excessive GWG: 25.8 (20.1 - 30.1)  P=0.099 |  |
| ***Outcome - mortality*** | | | | | |
| Parellada, 2014 | Excessive GWG vs. no excessive GWG | Perinatal mortality | 142 | OR (95% CI): 0.25 (0.01-5.48) |  |
| Yee, 2011 | Weight change greater than IOM guidelines vs. weight change within IOM guidelines | IUFD | 20 | aOR (95% CI): 1.10 (0.07-16.66) | Age, race/ethnicity, parity, education. |
| Asbjornsdottir, 2013 | Maternal weight gain >5kgs vs. weight gain ≤5kgs | Perinatal mortality | 58 | OR (95% CI): 0.40 (0.02-6.79) |  |
| Yee, 2011 | Weight change greater than IOM guidelines vs. weight change within IOM guidelines | IUFD | 33 | aOR (95% CI): 9.18 (0.60-141.39) | Age, race/ethnicity, parity, education. |
| ***Outcome – NICU admission*** | | | | | |
| Yee, 2011 | Weight change greater than IOM guidelines vs. weight change within IOM guidelines | NICU admission | 114 | aOR (95% CI): 1.89 (0.67-5.38) | Age, race/ethnicity, parity, education. |
| Yee, 2011 | Weight change greater than IOM guidelines vs. weight change within IOM guidelines | NICU admission | 153 | aOR (95% CI): 1.14 (0.53-2.47) | Age, race/ethnicity, parity, education. |
| Asbjornsdottir, 2013 | Maternal weight gain >5kgs vs. weight gain ≤5kgs | NICU admission | 56 | OR (95% CI): 2.33 (0.56-9.59) |  |
| ***Outcome - Other perinatal outcomes*** | | | | | |
| Parellada, 2014 | Excessive GWG vs. no excessive GWG | Composite perinatal morbidity | 142 | OR (95% CI): 1.97 (1.00-3.89) |  |
| Parellada, 2014 | Excessive GWG vs. no excessive GWG | Jaundice | 142 | OR (95% CI): 1.03 (0.36-2.96) |  |
| Parellada, 2014 | Excessive GWG vs. no excessive GWG | Transient tachypneoa of the newborn | 142 | OR (95% CI): 1.18 (0.40-3.46) |  |
| Parellada, 2014 | Excessive GWG vs. no excessive GWG | Neonatal hypoglycaemia | 142 | OR (95% CI): 2.31 (1.06-4.99) |  |
| Endo, 2018 | GWG (kg) | Perinatal complications | 11 | Mean (SD) (kg):  No complications: 7.7 (5.8)  Complications: 4.3 (2.7)  P=0.27 |  |
| Asbjornsdottir, 2013 | Maternal weight gain >5kgs vs. weight gain ≤5kgs | Perinatal morbidity | 58 | OR (95% CI): 4.43 (1.33-14.72) |  |
| Asbjornsdottir, 2013 | Maternal weight gain >5kgs vs. weight gain ≤5kgs | Major congenital malformation | 58 | OR (95% CI): 0.40 (0.02-6.79) |  |
| Asbjornsdottir, 2013 | Maternal weight gain >5kgs vs. weight gain ≤5kgs | Jaundice | 56 | OR (95% CI): 8.63 (0.46-158.92) |  |
| Asbjornsdottir, 2013 | Maternal weight gain >5kgs vs. weight gain ≤5kgs | Neonatal hypoglycaemia | 56 | OR (95% CI): 4.33 (1.06-17.57) |  |
| Asbjornsdottir | Maternal weight gain >5kgs vs. weight gain ≤5kgs | TTN (transient tachypnoea of the newborn) | 56 | OR (95% CI): 1.23 (0.22-6.87) |  |
| ***Outcome – diabetes control*** | | | | | |
| Parellada, 2014 | Excessive GWG vs. no excessive GWG | HbA1c 37 weeks | 142 | Median (range) (mmol/mol):  No excessive GWG: 41 (28-83)  Excessive GWG: 42 (31-64)  P=0.12 |  |
| Parellada, 2014 | Excessive GWG vs. no excessive GWG | Insulin at 37 weeks | 142 | OR (95% CI): 7.50 (0.92-60.89) |  |
| Parellada, 2014 | Excessive GWG vs. no excessive GWG | Insulin dose at 37 weeks U/kg/24h | 142 | Median (range) (U/kg/24h):  No excessive GWG: 0.78 (0.00–2.81)  Excessive GWG: 1.11 (0.00-2.60)  P=0.003 |  |
| Parellada, 2014 | Excessive GWG vs. no excessive GWG | Ketones 37 weeks | 142 | OR (95% CI): 0.43 (0.01-10.89) |  |
| Asbjornsdottir, 2013 | Maternal weight gain >5kgs vs. weight gain ≤5kgs | HBA1c at last visit (%) | 58 | Median (range) (%):  No excessive GWG: 5.7 (5.4-6.6)  Excessive GWG: 6.0 (4.8-8.2)  P=0.62 |  |
| Asbjornsdottir, 2013 | Maternal weight gain >5kgs vs. weight gain ≤5kgs | Insulin treatment at last visit | 58 | OR (95% CI): 0.31 (0.01-6.41) |  |
| Asbjornsdottir, 2013 | Maternal weight gain >5kgs vs. weight gain ≤5kgs | Insulin dose at last visit (IU/kg) | 58 | Median (range) (IU/kg):  No excessive GWG: 0.72 (0.12-1.80)  Excessive GWG: 1.29 (0.50-2.75)  P=0.003 |  |
| Rasmussen, 2010 | GWG to 34 weeks (kg) | Progression of retinopathy | 80 | Mean (SD) (GWG):  No progression: 11.9 (6.1)  Progression to retinopathy: 12.0 (6.7)  P=0.95 |  |

Abbreviations: BMI (Body Mass Index); CI (Confidence Interval); GWG (Gestational Weight Gain); HBA1c (haemoglobin A1c, a blood test that measures the average blood sugar level over theAssociations between glycaemic control and maternal and child health outcomes included past two to three months); IOM (Institute of Medicine); LGA (Large for Gestational Age); OR (Odds Ratio); SD (Standard Deviation); SGA (Small for Gestational Age); TTN (Transient Tachypnoea of the Newborn)

**Supplementary Table 14**. Findings on associations between glycaemic control and maternal and child health outcomes

| **First author, year** | **Exposure description** | **Pregnancy outcome** | **Total sample size** | **Result** | **Confounder adjustments** |
| --- | --- | --- | --- | --- | --- |
| ***Outcome - Caesarean delivery*** | | | | | |
| Abell, 2017 | HbA1c (%) | Caesarean section | 107 | aOR (95% CI): 1.03 (0.70-1.50) | Maternal age, maternal BMI, parity, smoking |
| Owens, 2015 | Glycaemic control: HbA1c (%) | Caesarean section - Elective | 108 | Mean (SD) (HbA1c %)  No elective caesarean section: 6.3 (1.1)  Elective caesarean section: 6.3 (0.8) |  |
| Owens, 2015 | Glycaemic control: HbA1c (%) | Caesarean section - Emergency | 108 | Mean (SD) (HbA1c %)  No emergency caesarean section: 6.3 (1.0)  Emergency caesarean section: 6 (1) |  |
| ***Outcome - Congenital anomaly*** | | | | | |
| Owens, 2015 | Glycaemic control: HbA1c (%) | Congenital Malformation | 108 | Mean (SD) (HbA1c %)  No congenital anomaly: 7.3 (1.8)  Congenital anomaly: 6.4 (0.7) |  |
| Abell, 2017 | HbA1c (%) | Congenital Malformation | 107 | aOR (95% CI): 1.56 (0.81-3.00) | Maternal age, maternal BMI |
| Murphy, 2021 | First trimester HbA1c:  Not reported assume <48mmol/mol (6.5%) vs >=48mmol/mol (6.5%) | Congenital Anomalies | Not reported | aOR (95% CI): 1.64 (1.23-2.21) | Not taking 5mg preconception folic acid |
| Ahmed, 2022 | HbA1c control:  Good HbA1c control (<7.4%) vs Poor HBA1c control (7.5-9.4%) | Any fetal anomaly | 172 | OR (95% CI): 2.27 (1.05-5.01) |  |
| Ahmed, 2022 | HbA1c control:  Good HbA1c control (<7.4%) vs Very poor HbA1c control (9.5%-11.4%) | Any fetal anomaly | 151 | OR (95% CI): 3.26 (1.23-8.64) |  |
| Ahmed, 2022 | HbA1c control:  Good HbA1c control (<7.4%) vs Worst HbA1c control (>11.5%) | Any fetal anomaly | 145 | OR (95% CI): 5.59 (1.84-17.01) |  |
| Arendt, 2021 | HbA1c (%):  <5.5 vs 5.5-<6.5 | Major malformations | 289 | Prevalence (95% CI): 5.2 (0.5; 5.1) |  |
| Arendt, 2021 | HbA1c (%):  <5.5 vs 6.5-<7.5 | Major malformations | 181 | Prevalence (95% CI): 4.6 (0.0; 9.7) |  |
| Arendt, 2021 | HbA1c (%):  <5.5 vs 7.5-<8.5 | Major malformations | 143 | Prevalence (95% CI): 11.1 (0.0; 23.0) |  |
| Arendt, 2021 | HbA1c (%):  <5.5 vs >=8.5 | Major malformations | 132 | Prevalence (95% CI): 12.5 (0.0; 28.7) |  |
| Ahmed, 2022 | HbA1c control:  Good HbA1c control (<7.4%) vs Poor HBA1c control (7.5-9.4%) | Chief (serious) anomaly (cardiovascular, central nervous system related, gastrointestinal related, ENT or facial, multiple reason, dermatological) | 172 | OR (95% CI): 2.51 (1.07-6.0) |  |
| Ahmed, 2022 | HbA1c control:  Good HbA1c control (<7.4%) vs Very poor HbA1c control (9.5%-11.4%) | Chief (serious) anomaly (cardiovascular, central nervous system related, gastrointestinal related, ENT or facial, multiple reason, dermatological) | 151 | OR (95% CI): 3.64 (1.28-10.4) |  |
| Ahmed, 2022 | HbA1c control:  Good HbA1c control (<7.4%) vs Worst HbA1c control (>11.5%) | Chief (serious) anomaly (cardiovascular, central nervous system related, gastrointestinal related, ENT or facial, multiple reason, dermatological) | 145 | OR (95% CI): 5.8 (1.77-18.91) |  |
| Ahmed, 2022 | HbA1c control:  Good HbA1c control (<7.4%) vs Poor HBA1c control (7.5-9.4%) | Inconsequential anomaly (cardiovascular, central nervous system related, gastrointestinal related, ENT or facial, multiple reason, dermatological) | 172 | OR (95% CI): 1.48 (0.36-5.88) |  |
| Ahmed, 2022 | HbA1c control:  Good HbA1c control (<7.4%) vs Very poor HbA1c control (9.5%-11.4%) | Inconsequential anomaly (cardiovascular, central nervous system related, gastrointestinal related, ENT or facial, multiple reason, dermatological) | 151 | OR (95% CI): 2.27 (0.45-11.6) |  |
| Ahmed, 2022 | HbA1c control:  Good HbA1c control (<7.4%) vs Worst HbA1c control (>11.5%) | Inconsequential anomaly (cardiovascular, central nervous system related, gastrointestinal related, ENT or facial, multiple reason, dermatological) | 145 | OR (95% CI): 1.91 (0.32-16.5) |  |
| ***Outcome - Hypertension*** | | | | | |
| Abell, 2017 | HbA1c (%) | Gestational hypertension | 107 | aOR (95% CI): 2.23 (1.27-3.90) | Maternal age, maternal BMI |
| Colatrella, 2009 | HbA1c (%) second trimester | Gestational hypertension | 76 | aOR (95% CI): 3.2 (0.33-31.1)  p=0.05 | Pre-pregnancy BMI, weight increase, age, parity, smoking, 1st, 2nd, 3rd trimester HbA1c |
| Morikawa, 2020 | HbA1c (%) first trimester:  HbA1c <7.2% vs HbA1c >=7.2% | Hypertensive disorder of pregnancy | 109 | aOR (95% CI): 8.5 (2.19-33.0)  p=0.0009 | Women without chronic hypertension |
| Owens, 2015 | Glycaemic control: Mean HbA1c (%) | Gestational hypertension | 108 | Mean (SD) (HbA1c %)  No gestational hypertension: 6.2 (1.1)  Gestational hypertension: 6.4 (0.9) |  |
| ***Outcome - Pre-eclampsia*** | | | | | |
| Owens, 2015 | Glycaemic control: Mean HbA1c (%) | Pre-eclampsia | 108 | Mean (SD) (HbA1c %)  No pre-eclampsia: 5.8 (0.6)  Pre-eclampsia: 6.3 (1.0) |  |
| Abell. 2017 | HbA1c (%) | Pre-eclampsia | 107 | aOR (95% CI): 1.13 (0.65-1.96) | Maternal age, maternal BMI |
| Oppermann, 2020 | HbA1c (%) <28 weeks | Pre-eclampsia | 109 | Mean (SD) (HbA1c %)  No pre-eclampsia: 7.6 (1.5)  Pre-eclampsia: 7.5 (1.5)  p=0.713 |  |
| ***Outcome - IOL*** | | | | | |
| Abell, 2017 | HbA1c (%) | Induction of labour | 107 | aOR (95% CI): 1.05 (0.74-1.51) | Maternal age, maternal BMI, smoking |
| ***Outcome - Jaundice*** | | | | | |
| Abell, 2017 | HbA1c (%) | Jaundice | 107 | aOR (95% CI): 0.80 (0.50-1.27) | Maternal age, maternal BMI, smoking, gestation at birth |
| ***Outcome - Late pregnancy HBa1c*** | | | | | |
| Murphy, 2021 | HbA1c (%) first trimester:  Not reported assume <48mmol/mol (6.5%) vs  >=48mmol/mol (6.5%) | Late pregnancy HbA1c <6.5% (48mmol/mol) | Not reported | aOR (95% CI): 0.94 (0.94-0.95) | Diabetes duration, antenatal appointment after 10 weeks |
| ***Outcome - LGA*** | | | | | |
| McLean, 2023 | Early pregnancy average glucose mmol/L | LGA | 41 | OR (95% CI): 1.84 (1.04-3.28)  p<0.05 |  |
| McLean, 2023 | Late pregnancy metrics average glucose mmol/L | LGA | 41 | OR (95% CI): 1.57 (0.91-2.69) |  |
| Olmos, 2009 | Second trimester mean blood glucose | LGA | 51 | Mean (SD) (Mean blood glucose)  Without LGA: 107.73 (3.83)  With LGA: 103.48 (4.84)  p=0.501 |  |
| Olmos, 2009 | Third trimester mean blood glucose | LGA | 51 | Mean (SD) (Mean blood glucose)  Without LGA: 104.11 (2.94)  With LGA: 112.35 (6.08)  p=0.238 |  |
| McLean, 2023 | Early pregnancy CV (%) | LGA | 41 | OR (95% CI): 0.99 (0.91-1.09) |  |
| McLean, 2023 | Early glycaemic targets CV:  CV>36% vs CV<36% | LGA | 41 | OR (95% CI): 2.00 (0.37-10.5) |  |
| McLean, 2023 | Late pregnancy metrics CV (%) | LGA | 41 | OR (95% CI): 1.06 (0.96-1.22) |  |
| McLean, 2023 | Early glycaemic targets CV:  CV>36% vs CV<36% | LGA | 41 | OR (95% CI): 0.80 (0.18-3.44) |  |
| McLean, 2023 | Early pregnancy *GMI (%) | LGA | 41 | OR (95% CI): 2.58 (0.83-7.91) |  |
| McLean, 2023 | Early glycaemic targets *GMI:  GMI >=6.5 vs GMI<6.5 | LGA | 41 | OR (95% CI): 0.46 (0.07-2.85) |  |
| McLean, 2023 | Late pregnancy metrics *GMI (%) | LGA | 41 | OR (95% CI): 3.35 (0.73-15.3) |  |
| McLean, 2023 | Early glycaemic targets *GMI:  GMI >=6.1 vs GMI<6.1 | LGA | 41 | OR (95% CI): 0.21 (0.04-0.94)  p<0.05 |  |
| McLean, 2023 | Early glycaemic targets *GMI:  GMI >=6.5 vs GMI<6.5 | LGA | 41 | OR (95% CI): 0.33 (0.03-3.37) |  |
| Ladfors, 2017 | First trimester HbA1c (mmol/mol) | LGA >2 SD of mean | 87 | OR (95% CI): 1.006 (0.960-1.053)  p=0.81 |  |
| Ladfors, 2017 | Second trimester HbA1c (mmol/mol) | LGA >2 SD of mean | 87 | OR (95% CI): 1.036 (0.977=1.099)  p=0.24 |  |
| Ladfors, 2017 | Third trimester HbA1c (mmol/mol) | LGA >2 SD of mean | 87 | OR (95% CI): 1.013 (0.942-1.089)  p=0.722 |  |
| Alexander, 2019 | First trimester HbA1c | LGA (BW and sex adjusted) | 253 | aOR (95% CI): 0.95 (0.71-1.29)  p=0.76 | Ethnicity, parity, insulin, hypertension, pre-pregnancy BI, first trimester A1C, delivery A1C |
| Alexander, 2019 | HbA1c at delivery | LGA (BW and sex adjusted) | 253 | aOR (95% CI): 1.40 (0.86-2.30)  p=0.18 | Ethnicity, parity, insulin, hypertension, pre-pregnancy BI, first trimester A1C, delivery A1C |
| Alexander, 2019 | First trimester HbA1c | LGA (GROW) | 253 | aOR (95% CI): 0.99 (0.95-1.03)  p=0.6 | Insulin, hypertension, first trimester A1C, delivery A1C, excess weight gain |
| Alexander, 2019 | HbA1c at delivery | LGA (GROW) | 253 | aOR (95% CI): 1.10 (1.02-1.19) p=0.01 | Insulin, hypertension, first trimester A1C, delivery A1C, excess weight gain |
| Abell, 2017 | HbA1c | LGA | 107 | aOR (95% CI): 1.56 (1.07-2.26) | Maternal age, maternal BMI |
| Murphy, 2021 | First trimester HbA1c:  <48mmol/mol (6.5%) vs  >=48mmol/mol (6.5%) | LGA | NR | aOR (95% CI): 1.51 (1.28-1.78) | Maternal age, diabetes duration, third trimester HbA1c |
| Rowan, 2009 | First trimester HbA1c | LGA | 180 | Mean (SD) (HbA1c)  No LGA: 8.2 (1.9)  LGA: 7.2 (1.5) |  |
| Kapustin, 2023 | Pre-pregnancy HbA1c level:  Pre-pregnancy HbA1c >6.5% vs Pre-pregnancy HbA1c <6.5% | LGA birth (>90^th^ percentile) | 214 | RR (95% CI): 0.43 (0.19-092) |  |
| McLean, 2023 | Early pregnancy HbA1c | LGA | 41 | OR (95% CI): 1.21 (0.84-1.71) |  |
| McLean, 2023 | Early glycaemic targets early HbA1c:  HbA1c >=6.5%/48mmol/mol vs HbA1c <6.5%/48mmol/mol | LGA | 41 | OR (95% CI): 0.37 (008-1.63) |  |
| McLean, 2023 | Late pregnancy metrics third trimester HbA1c (%) | LGA | 41 | OR (95% CI): 1.59 (0.83-3.05) |  |
| McLean, 2023 | Late glycaemic targets third trimester HbA1:  HbA1c >=6.1%/4.3mmol/mol vs HbA1c <6.1%/43mmol/mol | LGA | 41 | OR (95% CI): 0.08 (0.01-0.78)  P<0.05 |  |
| Olmos, 2009 | Second trimester HbA1c | LGA | 51 | Mean (SEM)* HbA1c  No LGA: 5.86 (0.25)  LGA: 6.01 (0.28)  p=0.695 |  |
| Olmos, 2009 | Third trimester HbA1c | LGA | 51 | Mean (SEM)* HbA1c  No LGA: 5.87 (0.19)  LGA: 6.11 (0.28)  p=0.496 |  |
| Olmos, 2009 | HbA1c antepartum | LGA | 51 | Mean (SEM)* HbA1c  No LGA: 5.93 (0.2)  LGA: 6.41 (0.24)  p=0.144 |  |
| McLean, 2023 | Late pregnancy metrics SD (mmol/L) | LGA | 41 | OR (95% CI): 3.39 (0.99-11.5) |  |
| McLean, 2023 | Early pregnancy **TAR (%) | LGA | 41 | OR (95% CI): 1.04 (1.01-1.08) p<0.05 |  |
| McLean, 2023 | Early glycaemic targets **TAR:  TAR>=25% vs TAR<25% | LGA | 41 | OR (95% CI): 0.31(0.08-1.21) |  |
| McLean, 2023 | Late pregnancy metrics **TAR (%) | LGA | 41 | OR (95% CI): 1.02 (0.98-1.05) |  |
| McLean, 2023 | Early glycaemic targets **TAR:  TAR>=25% vs TAR<25% | LGA | 41 | OR (95% CI): 0.28 (0.08-1.12) |  |
| McLean, 2023 | Early pregnancy ***TBR (%) | LGA | 41 | OR (95% CI): 0.91 (0.80-1.04) |  |
| McLean, 2023 | Early glycaemic targets ***TBR:  TBR>=4% vs TBR<4% | LGA | 41 | OR (95% CI): 1.26 (0.33-4.70) |  |
| McLean, 2023 | Late pregnancy metrics ***TBR (%) | LGA | 41 | OR (95% CI): 0.94 (0.85-1.03) |  |
| McLean, 2023 | Early glycaemic targets ***TBR: TBR>=4% vs TBR<4% | LGA | 41 | OR (95% CI): 1.44 (0.41-5.03) |  |
| McLean, 2023 | Early pregnancy ****TIR (%) | LGA | 41 | OR (95% CI): 0.96 (0.92-0.99)  p<0.05 |  |
| McLean, 2023 | Early glycaemic targets **** TIR:  TIR<=70% vs TIR>70% | LGA | 41 | OR (95% CI): 0.52 (0.14-1.91) |  |
| McLean, 2023 | Late pregnancy metrics **** TIR (%) | LGA | 41 | OR (95% CI): 0.98 (0.95-1.01) |  |
| McLean, 2023 | Early glycaemic targets **** TIR:  TIR<=70% vs TIR>70% | LGA | 41 | OR (95% CI): 0.50 (0.15-1.92) |  |
| ***Outcome - LGA placenta*** | | | | | |
| Rais, 2019 | HbA1c levels | LGA placenta (vs AGA placenta) | 136 | Mean (SD) (HbA1c)  AGA placenta: 7.5 (1.7)  LGA placenta: 7.3 (1.7)  p=0.42 |  |
| Rais, 2019 | Fasting blood glucose | LGA placenta (vs AGA placenta) | 136 | Mean (SD) Fasting blood glucose  AGA placenta: 96.4 (17.3)  LGA placenta: 101.2 (16.0)  p=0.1 |  |
| Rais, 2019 | Post-prandial glucose | LGA placenta (vs AGA placenta) | 136 | Mean (SD) post-prandial glucose  AGA placenta: 116.5 (19.5)  LGA placenta: 121.4 (20)  p=0.17 |  |
| ***Outcome - Neonatal hypoglycaemia*** | | | | | |
| McLean, 2023 | Early pregnancy average glucose (mmol/L) | Neonatal hypoglycaemia | 41 | OR (95% CI): 1.67 (1.01-2.78)  p<0.05 |  |
| McLean, 2023 | Late pregnancy metrics average glucose (mmol/L) | Neonatal hypoglycaemia | 41 | OR (95% CI) 1.97 (1.08-3.61) p<0.05 |  |
| McLean, 2023 | Early pregnancy CV (%) | Neonatal hypoglycaemia | 41 | OR (95% CI): 1.07 (0.97-1.18) |  |
| McLean, 2023 | Early glycaemic targets CV:  CV>=36% vs CV<36% | Neonatal hypoglycaemia | 41 | OR (95% CI): 0.85 (0.16-4.51) |  |
| McLean, 2023 | Late pregnancy metrics CV (%) | Neonatal hypoglycaemia | 41 | OR (95% CI): 1.13 (1.01-1.26)  p<0.05 |  |
| McLean, 2023 | Early glycaemic targets CV:  CV>=36% vs CV<36% | Neonatal hypoglycaemia | 41 | OR (95% CI): 0.18 (0.03-0.59)  p<0.05 |  |
| McLean, 2023 | Early pregnancy GMI (%) | Neonatal hypoglycaemia | 41 | OR (95% CI): 3.07 (0.92-10.2) |  |
| McLean, 2023 | Early glycaemic targets GMI:  GMI>=6.5 vs GMI<6.5 | Neonatal hypoglycaemia | 41 | OR (95% CI): 0.43 (0.10-1.79) |  |
| McLean, 2023 | Late pregnancy metrics GMI (%) | Neonatal hypoglycaemia | 41 | OR (95% CI): 3.90 (0.85-17.7) |  |
| McLean, 2023 | Early glycaemic targets GMI:  GMI>=6.1 vs GMI<6.1 | Neonatal hypoglycaemia | 41 | OR (95% CI): 0.13 (0.3-0.59)  p<0.05 |  |
| McLean, 2023 | Early glycaemic targets GMI:  GMI>=6.5 vs GMI<6.5 | Neonatal hypoglycaemia | 41 | OR (95% CI): 0.78 (0.11-5.33) |  |
| Owens. 2015 | Glycaemic control (Hba1c %) | Neonatal hypoglycaemia | 108 | Mean (SD) (HbA1c)  No neonatal hypoglycaemia: 5.9 (0.5)  Neonatal hypoglycaemia: 6.7 (1.5) |  |
| Abell, 2017 | HbA1c | Hypoglycaemia | 107 | aOR (95% CI): 1.46 (1.03-2.07) | Maternal age, maternal BMI |
| McLean, 2023 | Early pregnancy HbA1c (%) | Neonatal hypoglycaemia | 41 | OR (95% CI): 1.61 (1.01-2.54)  p<0.05 |  |
| McLean, 2023 | Third trimester late pregnancy metrics HbA1c (%) | Neonatal hypoglycaemia | 41 | OR (95% CI): 2.01 (0.91-4.65) |  |
| McLean, 2023 | Third trimester late glycaemic targets HbA1c:  Hba1c>=6.1%/>43mmol/mol vs HbA1c<6.1%/<43mmol/mol | Neonatal hypoglycaemia | 41 | OR (95% CI): 0.09 (0.01-0.91)  p<0.05 |  |
| Yamamoto, 2020 | In target HbA1c:  Above target vs Within target | Neonatal hypoglycaemia | 267 | aOR (95% CI): 0.7 (0.3-1.3)  p=0.23 | Covariates not specified |
| Yamamoto, 2020 | Intrapartum glycaemia | Neonatal hypoglycaemia | 267 | aOR (95% CI): 0.7 (0.3-1.4) | HbA1c |
| McLean, 2023 | Late pregnancy metrics SD (mmol/L) | Neonatal hypoglycaemia | 41 | OR (95% CI): 5.64 (1.47-21.5)  p<0.05 |  |
| McLean, 2023 | Early glycaemic TAR:  TAR>=25% vs TAR<25% | Neonatal hypoglycaemia | 41 | OR (95% CI): 0.15 (0.03-0.65)  p<0.05 |  |
| McLean, 2023 | Late pregnancy metrics TAR (%) | Neonatal hypoglycaemia | 41 | OR (95% CI): 1.05 (1.01-1.09)  p<0.05 |  |
| McLean, 2023 | Early glycaemic targets TAR:  TAR<=25% vs TAR<25% | Neonatal hypoglycaemia | 41 | OR (95% CI): 0.11 (0.02-0.52)  p<0.05 |  |
| McLean, 2023 | Early pregnancy TAR (%) | Neonatal hypoglycaemia | 41 | OR (95% CI): 1.04 (0.99-1.06) |  |
| Yamamoto, 2020 | Overt hypoglycaemia:  In target glucose vs Overt hyperglycaemia (>=8.6mmol/mol) | Neonatal hypoglycaemia | 184 | OR (95% CI): 2.0 (0.8717-4.5890)  p=0.1019 |  |
| McLean, 2023 | Early pregnancy TBR (%) | Neonatal hypoglycaemia | 41 | OR (95% CI): 0.93 (0.83-1.05) |  |
| McLean, 2023 | Early glycaemic targets TBR:  TBR >=4% vs TBR<4% | Neonatal hypoglycaemia | 41 | OR (95% CI): 1.05 (0.28-3.51) |  |
| McLean, 2023 | Late pregnancy metrics TBR (%) | Neonatal hypoglycaemia | 41 | OR (95% CI): 0.98 (0.90-1.07) |  |
| McLean, 2023 | Early glycaemic targets TBR:  TBR>=4% vs TBR<4% | Neonatal hypoglycaemia | 41 | OR (95% CI): 2.04 (0.58-7.17) |  |
| McLean, 2023 | Early pregnancy TIR (%) | Neonatal hypoglycaemia | 41 | OR (95% CI): 0.96 (093-1.0) |  |
| McLean, 2023 | Early glycaemic targets TIR:  TIR<=70% vs TIR>70% | Neonatal hypoglycaemia | 41 | OR (95% CI): 0.61 (0.04-0.67)  p<0.05 |  |
| McLean, 2023 | Late pregnancy metrics TIR (%) | Neonatal hypoglycaemia | 41 | OR (95% CI): 0.94 (0.90-0.99)  p<0.05 |  |
| McLean, 2023 | Early glycaemic targets TIR:  TIR<=70% vs TIR>70% | Neonatal hypoglycaemia | 41 | OR (95% CI): 0.08 (0.01-0.41)  p<0.05 |  |
| Yamamoto, 2020 | At least 25% in target (all glucose within target range of 3.5-6.5 mmol/L prior to delivery):  Less than 25% in target vs At least 25% in target | Neonatal hypoglycaemia | 267 | OR (95% CI): 0.3645 (0.1522-0.8732)  p=0.0236 |  |
| Yamamoto, 2020 | At least 50% in target (all glucose within target range of 3.5-6.5 mmol/L prior to delivery):  Less than 50% in target vs At least 50% in target | Neonatal hypoglycaemia | 267 | OR (95% CI): 0.4616 (0.2106-1.0120)  p=0.0536 |  |
| Yamamoto, 2020 | In target glucose:  Out of target glucose vs In target glucose | Neonatal hypoglycaemia | 267 | OR (95% CI): 0.6474 (0.3413-1.2281)  p=0.1832 |  |
| ***Outcome - NICU admission*** | | | | | |
| Abell, 2017 | HbA1c | Special care nursery admission | 107 | aOR (95% CI): 1.46 (0.89-2.40) | Maternal age, maternal BMI |
| Abell, 2017 | HbA1c | NICU admission | 107 | aOR (95% CI): 0.86 (0.42-1.79) | Maternal age, maternal BMI |
| ***Outcome - Perinatal complications*** | | | | | |
| Endo, 2018 | Pre-gestational HbA1c (%) | Perinatal complications | 11 | Mean (SD) (HbA1c %)  No perinatal complications: 7.2 (2)  Perinatal complications: 7 (1)  p=0.89 |  |
| Endo, 2018 | Mean HbA1c during pregnancy (%) | Perinatal complications | 11 | Mean (SD) (HbA1c %)  No perinatal complications: 6.4 (1.7)  Perinatal complications: 6 (0.5)  p=0.67 |  |
| ***Outcome - Perinatal death or congenital anomaly*** | | | | | |
| Abell, 2017 | HbA1c | Perinatal death | 107 | aOR (95% CI): 2.80(1.09-7.17) | Maternal age, maternal BMI |
| Owens, 2015 | Glycaemic control HbA1c (%) | Miscarriage | 108 | Mean (SD) (HbA1c %)  No miscarriage: 6.2 (0.8)  Miscarriage: 7.7 (1.6) |  |
| Murphy, 2021 | First trimester HbA1c:  <or equal to 43 mmol/mol vs 44-52 mmol/mol | Averse pregnancy outcome (stillbirth, neonatal death, and any know congenital anomaly incl. pregnancies which miscarried or were terminated) | Not reported | OR (95% CI): 1.1408 (0.798101.6306)  p=0.4699 |  |
| Murphy, 2021 | First trimester HbA1c:  <or equal to 43 mmol/mol vs 53-63 mmol/mol | Averse pregnancy outcome (stillbirth, neonatal death, and any know congenital anomaly incl. pregnancies which miscarried or were terminated) | Not reported | OR (95% CI): 1.6546 (1.1610-2.3581)  P=0.0053 |  |
| Murphy, 2021 | First trimester HbA1c: <or equal to 43 mmol/mol vs 74-74 mmol/mol | Averse pregnancy outcome (stillbirth, neonatal death, and any know congenital anomaly incl. pregnancies which miscarried or were terminated) | Not reported | OR (95% CI): 1.5794 (1.0367-2.4062)  p=0.0333 |  |
| Murphy, 2021 | First trimester HbA1c:  <or equal to 43 mmol/mol vs 75-85 mmol/mol | Averse pregnancy outcome (stillbirth, neonatal death, and any know congenital anomaly incl. pregnancies which miscarried or were terminated) | Not reported | OR (95% CI): 2.3589 (1.5144-3.6745)  p=0.0001 |  |
| Murphy, 2021 | First trimester HbA1c:  <or equal to 43 mmol/mol vs 86 mmol/mol and over | Averse pregnancy outcome (stillbirth, neonatal death, and any know congenital anomaly incl. pregnancies which miscarried or were terminated) | Not reported | OR (95% CI): 3.5246 (2.3749-5.2308)  p<0.0001 |  |
| ***Outcome - Polyhydramnios*** | | | | | |
| Owens, 2015 | Glycaemic control Hba1c (%) | Polyhydramnios | 108 | Mean (SD) (Hba1c %)  No polyhydramnios: 6.2 (1.3)  Polyhydramnios: 6.7 (0.9) |  |
| ***Outcome - Preterm birth*** | | | | | |
| Murphy, 2021 | Glycaemic control (HbA1c):  Suboptimal early glycaemic control with suboptimal late glycaemic control vs suboptimal early glycaemic control with improved late glycaemic control (at target) | Preterm birth | 1386 | No preterm birth: 21.6%  Preterm birth: 35.7%  p<0.001 |  |
| Owens, 2015 | Glycaemic control Hba1c (%) | Preterm birth | 108 | Mean (SD) (HbA1c %)  No preterm birth: 6.2 (0.8)  Preterm birth: 6.6 (1.4) |  |
| Abell, 2017 | HbA1c | Preterm birth | 107 | aOR (95% CI): 1.32 (0.86-2.04) | Maternal age, maternal BMI, pre-eclampsia |
| Søholm, 2021 | HbA1c early pregnancy | Gestational age at delivery (preterm <37/40 weeks) | 86 | Mean (SD)  No preterm delivery: 4.9 (11)  Preterm delivery: 52 (10)  p=0.45 | Birthweight SD score was used to describe how far offspring birth weight was from the mean of the Nordic background population adjusted for gestational age and sex |
| Murphy, 2021 | First trimester HbA1c:  Not reported assume <48mmol/mol (6.5%) vs  >=48mmol/mol (6.5%) | Preterm birth before 37 weeks gestation | Not reported | aOR (95% CI): 1.71 (1.44-2.04) | Ethnicity, diabetes duration, third trimester HbA1c |
| ***Outcome - Progression of retinopathy*** | | | | | |
| Rasmussen, 2010 | HbA1c (%) | Progression of retinopathy | 80 | Mean (SD) (HbA1c %)  No progression of retinopathy: 6.4 (1)  Progression of retinopathy: 7.2 (1.2)  p=0.07 |  |
| ***Outcome - SGA*** | | | | | |
| Abell, 2017 | HbA1c | SGA | 107 | aOR (95% CI): 1.28 (0.81-2.01) | Maternal age, maternal BMI, parity |
| Rowan, 2009 | First trimester HbA1c | SGA | 159 | Mean (SD) (HbA1c):  No SGA: 8.2 (1.9)  SGA: 7.5 (1.8) |  |
| Feig, 2022 | First HbA1c in pregnancy % | SGA | 460 | Mean (SD) (HbA1c):  No SGA: 7.17 (1.71)  SGA: 7.23 (1.38)  p=0.815 |  |
| Feig, 2022 | First Hba1c in pregnancy mmol/mol | SGA | 460 | Mean (SD) (HbA1c):  No SGA: 54.9 (18.7)  SGA: 55.6 (15.1)  p=0.815 |  |
| Feig, 2022 | First HbA1c at entry % | SGA | 460 | Mean (SD) (HbA1c):  No SGA: 6.38 (1.20)  SGA: 6.30 (0.69)  p=0.711 |  |
| Feig, 2022 | First HbA1c at entry mmol/mol | SGA | 460 | Mean (SD) (HbA1c):  No SGA: 46.2 (13.1)  SGA: 45.4 (7.5)  p=0.711 |  |

Abbreviations: AGA (Appropriate for Gestational Age); BMI (Body Mass Index); CI (Confidence Interval); ENT (Ear, Nose and Throat); CGM (Continuous Glucose Monitor); CV (coefficient of variation); GMI (Glucose Management Indicator); HBA1c (haemoglobin A1c, a blood test that measures the average blood sugar level over the past two to three months); LGA (Large for Gestational Age); OR (Odds Ratio); NR (Not Reported); SD (Standard Deviation); SGA (Small for Gestational Age); TAR (Time Above Range); TBR (Time Below Range); TIR (Time in Range).

*Glucose Management Indicator (GMI) targets were based on the HbA1c targets <6.5% (<48 mmol/mol) in early and<6.1% (<43 mmol/mol) in late pregnancy.

** Time Above Range (TAR) percentage of all time with Continuous Glucose Monitor (CGM) glucose values above 7.8 mmol (>140 mg/dL).

*** Time Below Range (TBR) percentage of all time with Continuous Glucose Monitor (CGM) glucose values below 3.5 mmol/L (<63 mg/dL).

****Time in Range (TIR) percentage of all time with CGM glucose values within the pregnancy-specific target range of 3.5–7.8 mmol/L (63–140 mg/dL).

**Supplementary Table 15**. Findings on associations between blood pressure and maternal and offspring health outcomes

| **First author, year** | **Exposure description** | **Pregnancy outcome** | **Total sample size** | **Result** | **Confounder adjustments** |
| --- | --- | --- | --- | --- | --- |
| ***Outcome - Preterm birth*** | | | | | |
| Søholm, 2021 | Systolic blood pressure (early pregnancy) | Gestational age at delivery (preterm<37/40) | 86 | Mean (SD) (mmHG):  No preterm delivery: 123 (13)  Preterm delivery: 125 (10)  p=NS | Birthweight |
| Søholm, 2021 | Diastolic blood pressure (early pregnancy) | Gestational age at delivery (preterm<37/40) | 86 | Mean (SD) (mmHG):  No preterm delivery: 79 (9)  Preterm delivery: 79 (9)  p=NS | Birthweight |
| Søholm, 2021 | Antihypertensive treatment (early pregnancy) | Gestational age at delivery (preterm<37/40) | 86 | aOR (95% CI): 1.4 (0.256 - 7.63) | Birthweight |
| Søholm, 2021 | Antihypertensive treatment (late pregnancy) | Gestational age at delivery (preterm<37/40) | 77 | aOR (95% CI): 1.54 (0.498 – 4.78) | Birthweight |
| ***Outcome - Progression of retinopathy*** | | | | | |
| Rasmussen, 2010 | Systolic blood pressure | Progression of retinopathy | 80 | Mean (SD) (mmHG):  Progression of retinopathy: 125 (18)  No progression of retinopathy: 121(13)  p=NS |  |
| Rasmussen, 2010 | Diastolic blood pressure | Progression of retinopathy | 80 | Mean (SD) (mmHG):  Progression of retinopathy: 76 (10)  No progression of retinopathy: 72 (9)  p=NS |  |
| ***Outcome - Insulin dose ≥2 units/kg at delivery*** | | | | | |
| Nadeau, 2021 | Chronic hypertension | Insulin dose ≥2 units/kg at delivery | 160 | OR (95% CI): 1.129 (0.579 - 2.20) |  |

Abbreviations: NS (Not Significant); OR (Odds Ratio); SD (Standard Deviation)

**Supplementary Table 16**. Findings on associations between anxiety and/or depressive symptoms and maternal and child health outcomes

| **First author, year** | **Exposure description** | **Pregnancy outcome** | **Total sample size** | **Result** | **Confounder adjustments** |
| --- | --- | --- | --- | --- | --- |
| Asbjornsdottir, 2021 | T2D anxiety and or depression symptoms | Total gestational weight gain (kg) | 90 | Median (IQR)  No anxiety and/or depression symptoms: 10.8 (7.7-14.6)  Anxiety and/or depression symptoms: 9.1 (5.6-14.0)  p=NS |  |
| Asbjornsdottir, 2021 | T2D anxiety and or depression symptoms | Total gestational weight gain/week (kg) | 90 | Median (IQR)  No anxiety and/or depression symptoms: 0.29 (0.22-0.42)  Anxiety and/or depression symptoms: 0.28 (0.16-0.45)  p=NS |  |
| Asbjornsdottir, 2021 | T2D anxiety and or depression symptoms | Sleep early pregnancy (hrs) | 90 | Median (IQR)  No anxiety and/or depression symptoms: 8 (7-8.5)  Anxiety and/or depression symptoms: 7.5 (7-8)  p=NS |  |
| Asbjornsdottir, 2021 | T2D anxiety and or depression symptoms | Sleep late pregnancy (hrs) | 72 | Median (IQR)  No anxiety and/or depression symptoms: 7 (6-8)  Anxiety and/or depression symptoms: 8 (6-9)  p=NS |  |
| Asbjornsdottir, 2021 | T2D anxiety and or depression symptoms | HbA1c early pregnancy (mmol/mol) | 90 | Mean (SD)  No anxiety and/or depression symptoms: 49 (11)  Anxiety and/or depression symptoms: 52 (14)  p=NS |  |
| Asbjornsdottir, 2021 | T2D anxiety and or depression symptoms | HbA1c late pregnancy (mmol/mol) | 90 | Mean (SD)  No anxiety and/or depression symptoms: 40 (4)  Anxiety and/or depression symptoms: 43 (8)  P=0.04 |  |
| Asbjornsdottir, 2021 | T2D anxiety and or depression symptoms | Women on insulin treatment | 90 | OR (95% CI): 0.4706 (0.0988 to 2.2414)  p=NS |  |
| Asbjornsdottir, 2021 | T2D anxiety and or depression symptoms | Insulin dose/kg bodyweight in late pregnancy (IU/kg) | 90 | Median (IQR)  No anxiety and/or depression symptoms: 0.89 (0.65-1.45)  Anxiety and/or depression symptoms: 1.08 (0.81-1.76)  p=NS |  |
| Asbjornsdottir, 2021 | T2D anxiety and or depression symptoms | Smoking | 90 | OR (95% CI): 1 (0.2613 to 3.8264)  p=NS |  |
| Asbjornsdottir, 2021 | T2D anxiety and or depression symptoms | Gestational age at delivery (days) | 90 | Median (IQR)  No anxiety and/or depression symptoms: 267 (260-271)  Anxiety and/or depression symptoms: 262 (260-268)  P=0.08 |  |
| Asbjornsdottir, 2021 | T2D anxiety and or depression symptoms | Preterm delivery (<37 weeks) | 90 | OR (95% CI): 1.0621 (0.3630 to 3.1077)  p=NS |  |
| Asbjornsdottir, 2021 | T2D anxiety and or depression symptoms | Caesarean section | 90 | OR (95% CI): 1.3478 (0.5777 to 3.1447)  p=NS |  |
| Asbjornsdottir, 2021 | T2D anxiety and or depression symptoms | LGA (above 90th percentile using Nordic growth curves) | 90 | aOR (95% CI): 1.6429 (0.5541 to 4.8706)  p=NS | Nordic growth curves adjusted for gestational age and infant sex |
| Asbjornsdottir, 2021 | T2D anxiety and or depression symptoms | SGA (below 10th percentile using Nordic growth curves) | 90 | aOR (95% CI): 2.5 (0.6522 to 9.5831)  p=NS | Nordic growth curves adjusted for gestational age and infant sex |
| Asbjornsdottir, 2021 | T2D anxiety and or depression symptoms | Major congenital malformation | 90 | OR (95% CI): 0.2877 (0.0134 to 6.1701)  p=NS |  |
| Asbjornsdottir, 2021 | T2D anxiety and or depression symptoms | Neonatal hypoglycaemia | 86 | OR (95% CI): 0.3621 (0.0938 to 1.3977)  p=NS |  |
| Asbjornsdottir, 2021 | T2D anxiety and or depression symptoms | Neonatal jaundice | 90 | OR (95% CI): 0.8065 (0.2465 to 2.6379)  p=NS |  |
| Asbjornsdottir, 2021 | T2D anxiety and or depression symptoms | Neonatal transient tachypnoea | 90 | OR (95% CI): 1.5625 (0.3626 to 6.6960)  p=NS |  |

Abbreviations: CI (Confidence Interval); IQR (Interquartile Range); LGA (Large for Gestational Age); NS (Not Significant); OR (Odds Ratio); SGA (Small for Gestational Age); T2D (Type 2 Diabetes)

**Supplementary Table 17**. Findings on associations between smoking and maternal and child health outcomes

| **First author, year** | **Exposure description** | **Pregnancy outcome** | **Total sample size** | **Result** | **Confounder adjustments** |
| --- | --- | --- | --- | --- | --- |
| ***Outcome - LGA*** | | | | | |
| Ladfors, 2017 | Smoking during pregnancy:  Non-smokers vs smokers | LGA >2 SD of mean | 87 | OR (95% CI):  0.1198 (0.0068 to 2.1266) |  |
| Rowan, 2009 | Smoking during pregnancy:  Non-smokers vs smokers at 1^st^ antenatal visit | LGA | 180 | OR (95% CI):  0.4972 (0.1776 to 1.3917) |  |
| ***Outcome - Other*** | | | | | |
| Colatrella, 2009 | Smoking during pregnancy:  Non-smokers vs smokers | Chronic hypertension | 76 | aOR (95%CI):  0.05 (0.006- 0.05) | Pre-pregnancy BMI, weight increase, age, parity, smoking and 1st, 2nd and 3rd trimester HbA1c |
| Rasmussen, 2010 | Smoking during pregnancy:  Non-smokers vs smokers | Progression of retinopathy | 80 | OR (95% CI):  3.013 (0.7525 to 12.0644) |  |
| Kekki, 2021 | Smoking during pregnancy:  Non-smokers vs smokers | Severe birth injury | 548 | OR (95% CI):  0.80 (0.17-3.82) |  |
| Oppermann, 2020 | Smoking during pregnancy:  Non-smokers vs smokers | Preeclampsia | 127 | OR (95% CI):  0.6964 (0.1780 to 2.7248) |  |
| Søholm, 2021 | Smoking during pregnancy:  Non-smokers vs smokers | Gest age at delivery (preterm <37/40) | 86 | OR (95% CI):  0.8 (0.087 to 7.34) |  |
| Nadeau, 2021 | Smoking during pregnancy:  Non-smokers vs smokers | Insulin dose ≥2 units/kg at delivery | 160 | OR (95% CI):  1.5424 (0.6451 to 3.6875) |  |
| Rowan, 2009 | Smoking during pregnancy:  Non-smoker vs Smoker - women who were smoking at first antenatal visit | SGA | 159 | OR (95% CI):  2.5000 (1.0556 to 5.9209) |  |
| Stafl, 2023 | Smoking during pregnancy:  Non-smoker vs Smoking during pregnancy | Missed 1 or more appointments | 902 | OR (95% CI):  2.1493 (1.3542 to 3.4112) |  |
| Feig, 2022 | Smoking during pregnancy:  Never smoked vs Smoked before pregnancy | SGA | 410 | OR (95% CI):  0.5404 (0.1859 to 1.5702) |  |
| Feig, 2022 | Smoking during pregnancy: Never smoked vs Smoked during pregnancy but stopped | SGA | 365 | OR (95% CI):  0.9536 (0.2120 to 4.2883) |  |
| Feig, 2022 | Smoking during pregnancy:  Never smoked vs Smoked during pregnancy and continued | SGA | 377 | OR (95% CI):  0.2702 (0.0358 to 2.0381) |  |

Abbreviations: CI (Confidence Interval); LGA (Large for Gestational Age); OR (Odds Ratio); SGA (Small for Gestational Age).

**Supplementary Table 18**. Findings on associations between folic acid supplement use and maternal and child health outcomes

| **First author, year** | **Exposure description** | **Pregnancy outcome** | **Total sample size** | **Result** | **Confounder adjustments** |
| --- | --- | --- | --- | --- | --- |
| ***Outcome - Congenital anomalies*** | | | | | |
| Roland, 2005 | No folic acid supplementation vs folic acid supplementation | Congenital anomaly | 146 | aOR (95% CI):  0.3 (0.09–1.0) | Ethnicity, maternal age, diabetes type, oral hypoglycaemic agents, insulin use, HbA1cin first trimester, body mass index (BMI), folic acid, pre-pregnancy care, gestational age and smoking at conception. |
| Murphy, 2021 | Taking folic acid as a specific 5mg preconception supplement vs Not taking 5mg preconception folic acid | Congenital anomaly | Not reported | aOR (95% CI):  1.10 (0.76-1.58) | First trimester HbA1c >= 48mmol/mol (6.5%) |

Abbreviations: aOR (Adjusted Odds Ratio); BMI (Body Mass Index); CI (Confidence Interval); HBA1c (haemoglobin A1c, a blood test that measures the average blood sugar level over the past two to three months); OR (Odds Ratio)

**Supplementary Table 19**. Findings on associations between breastfeeding and maternal and child health outcomes

| **First author, year** | **Exposure description** | **Pregnancy outcome** | **Total sample size** | **Result** | **Confounder adjustments** |
| --- | --- | --- | --- | --- | --- |
| Longmore, 2022 | Breastfeeding at 6 months:  No predominant breastfeeding vs Predominant breastfeeding | Weight for age z score at 14 months | 57 | β coefficient (95% CI):  -0.10 (-0.55, 0.36) |  |
| Longmore, 2022 | Breastfeeding at 6 months:  No predominant breastfeeding vs Predominant breastfeeding | Weight for length z score at 14 months | 45 | β coefficient (95% CI):  -0.11 (-0.68, 0.46) |  |
| Longmore, 2022 | Breastfeeding at 6 months:  No predominant breastfeeding vs Predominant breastfeeding | Body mass index z score at 14 months | 45 | β coefficient (95% CI):  -0.10 (-0.67, 0.47) |  |
| Cordero, 2022 | Breastfeeding intention:  Intended partial breastfeed vs Intention to exclusively breastfeed | Exclusive breastfeeding at discharge | 294 | aOR (95% CI):  6.57 (2.24 - 19.28) | Chronic hypertension, preeclampsia, mothers age, race, health insurance type, smoking during pregnancy, BMI, parity, mode of delivery, prior breastfeeding, breastfeeding within two hours from birth, prematurity, AGA, SGA, LGA, admission to NICU, neonatal hypoglycaemia and infant length of stay |
| Cordero, 2022 | Breastfeeding intention:  no intention to breastfeed vs intention for any breastfeeding initiation | Breastfeeding initiation (any breastfeeding) | 294 | aOR (95% CI):  5.05 (2.54–10.04) | Chronic hypertension, preeclampsia, mothers age, race, health insurance type, smoking during pregnancy, BMI, parity, mode of delivery, prior BF, BF within two hours from birth, prematurity, AGA, SGA, LGA, admission to NICU, neonatal hypoglycaemia and infant length of stay |

Abbreviations: AGA (Appropriate for Gestational Age); aOR (Adjusted Odds Ratio); BMI (Body Mass Index); CI (Confidence Interval); LGA (Large for Gestational Age); NICU (Neonatal Intensive Care Unit); SGA (Small for Gestational Age).

**References**

Abell SK, Boyle JA, de Courten B, Soldatos G, Wallace EM, Zoungas S, et al. Impact of type 2 diabetes, obesity and glycaemic control on pregnancy outcomes. Aust N Z J Obstet Gynaecol. 2017;57(3):308-14.

Ahmed M, Mujeeb-Ur-Rehman Abro S, Khuhro BN, Bhanbhro FI, Shaikh A. Risk of Congenital Anomalies in Pregnant Women with Type 2 Diabetes. Pakistan Journal of Medical & Health Sciences. 2022;16(11):141-.

Allen AJ, Snowden JM, Lau B, Cheng Y, Caughey AB. Type-2 diabetes mellitus: does prenatal care affect outcomes? J Matern Fetal Neonatal Med. 2018;31(1):93-7.

Alexander LD, Tomlinson G, Feig DS. Predictors of Large-for-Gestational-Age Birthweight Among Pregnant Women With Type 1 and Type 2 Diabetes: A Retrospective Cohort Study. Can J Diabetes. 2019;43(8):560-6.

Alrais M, Ward C, Cornthwaite JAA, Chen HY, Chauhan SP, Sibai BM, et al. Type 2 diabetes and neonatal hypoglycemia: role of route of delivery and insulin infusion. J Matern Fetal Neonatal Med. 2022;35(25):7445-51.

Arendt LH, Pedersen LH, Pedersen L, Ovesen PG, Henriksen TB, Lindhard MS, et al. Glycemic Control in Pregnancies Complicated by Pre-Existing Diabetes Mellitus and Congenital Malformations: A Danish Population-Based Study. Clin Epidemiol. 2021;13:615-26.

Asbjörnsdóttir B, Rasmussen SS, Kelstrup L, Damm P, Mathiesen ER. Impact of restricted maternal weight gain on fetal growth and perinatal morbidity in obese women with type 2 diabetes. Diabetes Care. 2013;36(5):1102-6.

Ásbjörnsdóttir B, Vestgaard M, Ringholm L, Andersen LLT, Jensen DM, Damm P, et al. Effect of motivational interviewing on gestational weight gain and fetal growth in pregnant women with type 2 diabetes. BMJ Open Diabetes Res Care. 2019;7(1):e000733.

Ásbjörnsdóttir B, Vestgaard M, Do NC, Ringholm L, Andersen LLT, Jensen DM, et al. Prevalence of anxiety and depression symptoms in pregnant women with type 2 diabetes and the impact on glycaemic control. Diabet Med. 2021;38(3):e14506.

Fishel Bartal M, Ward C, Refuerzo JS, Ashimi SS, Joycelyn CA, Chen HY, et al. Basal Insulin Analogs versus Neutral Protamine Hagedorn for Type 2 Diabetics. Am J Perinatol. 2020;37(1):30-6.

Cesta CE, Rotem R, Bateman BT, Chodick G, Cohen JM, Furu K, et al. Safety of GLP-1 Receptor Agonists and Other Second-Line Antidiabetics in Early Pregnancy. JAMA Intern Med. 2023;184(2):144-52.

Cordero L, Stenger MR, Landon MB, Nankervis CA. Impact of excessive gestational weight gain on exclusive breastfeeding among women with Type 1 and Type 2 diabetes and obesity. PLoS One. 2022;17(11):e0277599.

Colatrella A, Braucci S, Festa C, Bianchi P, Fallucca F, Mattei L, et al. Hypertensive disorders in normal/over-weight and obese type 2 diabetic pregnant women. Exp Clin Endocrinol Diabetes. 2009;117(8):373-7.

Cyganek K, Hebda-Szydlo A, Skupien J, Katra B, Janas I, Borodako A, et al. Glycemic control and pregnancy outcomes in women with type 2 diabetes from Poland. The impact of pregnancy planning and a comparison with type 1 diabetes subjects. Endocrine. 2011;40(2):243-9.

Do NC, Vestgaard M, Ásbjörnsdóttir B, Nørgaard SK, Andersen LLT, Jensen DM, et al. Unchanged Prevalence of Preeclampsia After Implementation of Prophylactic Aspirin for All Pregnant Women With Preexisting Diabetes: A Prospective Cohort Study. Diabetes Care. 2021.

Egan AM, Danyliv A, Carmody L, Kirwan B, Dunne FP. A Prepregnancy Care Program for Women With Diabetes: Effective and Cost Saving. J Clin Endocrinol Metab. 2016;101(4):1807-15.

Endo S, Saisho Y, Miyakoshi K, Ochiai D, Matsumoto T, Kawano Y, et al. Association of Maternal Factors with Perinatal Complications in Pregnancies Complicated with Diabetes: A Single-Center Retrospective Analysis. J Clin Med. 2018;7(1).

Feghali MN, Caritis SN, Catov JM, Scifres CM. Glycemic Control and Pregnancy Outcomes in Women with Type 2 Diabetes Treated with Oral Hypoglycemic Agents. Am J Perinatol. 2017;34(7):697-704.

Feig DS, Zinman B, Asztalos E, Donovan LE, Shah PS, Sanchez JJ, et al. Determinants of Small for Gestational Age in Women With Type 2 Diabetes in Pregnancy: Who Should Receive Metformin? Diabetes Care. 2022;45(7):1532-9.

Gaudio M, Dozio N, Feher M, Scavini M, Caretto A, Joy M, et al. Trends in Factors Affecting Pregnancy Outcomes Among Women With Type 1 or Type 2 Diabetes of Childbearing Age (2004-2017). Front Endocrinol (Lausanne). 2020;11:596633.

Isabey EP, Pylypjuk CL. The Relationship between Fetal Abdominal Wall Thickness and Intrapartum Complications amongst Mothers with Pregestational Type 2 Diabetes. J Diabetes Res. 2021;2021:5544599.

Kallas-Koeman MM, Khandwala F, Donovan LE. Rate of Preconception Care in Women with Type 2 Diabetes Still Lags behind that of Women with Type 1 Diabetes. Canadian Journal of Diabetes. 2012;36:170-4.

Kapustin RV, Kopteeva EV, Alekseenkova EN, Tsybuk EM, Arzhanova ON, Kogan IY. Risk factors for shoulder dystocia during labor in women with diabetes mellitus. Obstetrics and Gynecology. 2022(9):54-63.

Kapustin R, Kopteeva E, Tiselko A, Alekseenkova E, Korenevsky A, Shelaeva E, et al. Diabetes and pregnancy study (DAPSY): a 10-year single-center cohort study of pregnancies affected by diabetes. Archives of Gynecology and Obstetrics. 2023;309(6):2643-51.

Kekki M, Tihtonen K, Salonen A, Koukkula T, Gissler M, Laivuori H, et al. Severe birth injuries in neonates and associated risk factors for injury in mothers with different types of diabetes in Finland. Int J Gynaecol Obstet. 2022;159(1):195-203.

Kjerpeseth LJ, Cesta CE, Furu K, Engeland A, Gissler M, Gulseth HL, et al. Metformin Versus Insulin and Risk of Major Congenital Malformations in Pregnancies With Type 2 Diabetes: A Nordic Register-Based Cohort Study. Diabetes Care. 2023;46(8):1556-64.

Ladfors L, Shaat N, Wiberg N, Katasarou A, Berntorp K, Kristensen K. Fetal overgrowth in women with type 1 and type 2 diabetes mellitus. PLoS One. 2017;12(11):e0187917.

Lin SF, Chang SH, Kuo CF, Lin WT, Chiou MJ, Huang YT. Association of pregnancy outcomes in women with type 2 diabetes treated with metformin versus insulin when becoming pregnant. BMC Pregnancy Childbirth. 2020;20(1):512.

Longmore DK, Titmuss A, Barr E, Barzi F, Simmonds A, Lee IL, et al. Breastfeeding and infant growth in offspring of mothers with hyperglycaemia in pregnancy: The pregnancy and neonatal diabetes outcomes in remote Australia study. Pediatr Obes. 2022;17(6):e12891.

Maple-Brown LJ, Lindenmayer G, Barzi F, Whitbread C, Connors C, Moore E, et al. Real-world experience of metformin use in pregnancy: Observational data from the Northern Territory Diabetes in Pregnancy Clinical Register. J Diabetes. 2019;11(9):761-70.

McLean A, Barr E, Tabuai G, Murphy HR, Maple-Brown L. Continuous Glucose Monitoring Metrics in High-Risk Pregnant Women with Type 2 Diabetes. Diabetes Technol Ther. 2023;25(12):836-44.

Morikawa M, Kato-Hirayama E, Mayama M, Saito Y, Nakagawa K, Umazume T, et al. Glycemic control and fetal growth of women with diabetes mellitus and subsequent hypertensive disorders of pregnancy. PLoS One. 2020;15(3):e0230488.

Murphy HR, Roland JM, Skinner TC, Simmons D, Gurnell E, Morrish NJ, et al. Effectiveness of a regional prepregnancy care program in women with type 1 and type 2 diabetes: benefits beyond glycemic control. Diabetes Care. 2010;33(12):2514-20.

Murphy HR, Bell R, Cartwright C, Curnow P, Maresh M, Morgan M, et al. Improved pregnancy outcomes in women with type 1 and type 2 diabetes but substantial clinic-to-clinic variations: a prospective nationwide study. Diabetologia. 2017;60(9):1668-77.

Murphy HR, Howgate C, O'Keefe J, Myers J, Morgan M, Coleman MA, et al. Characteristics and outcomes of pregnant women with type 1 or type 2 diabetes: a 5-year national population-based cohort study. Lancet Diabetes Endocrinol. 2021;9(3):153-64.

Nadeau HCG, Maxted ME, Madhavan D, Pierce SL, Feghali M, Scifres C. Insulin Dosing, Glycemic Control, and Perinatal Outcomes in Pregnancies Complicated by Type-2 Diabetes. Am J Perinatol. 2021;38(6):535-43.

Nørgaard SK, Do NC, Ásbjörnsdóttir B, Secher AL, Ringholm L, Damm P, et al. Prepregnancy body mass index and offspring birth weight in women with Type 1 and Type 2 diabetes. Journal of Pregnancy and Child Health. 2016;3(2).

Olmos PR, Araya-Del-Pino AP, González-Carvello CA, Laso-Ulloa P, Hodgson MI, Irribarra V, et al. Near-optimal glycemic control in Chilean women with pregestational type-2 diabetes: persistent macrosomia relates to maternal pre-pregnancy overweight. Diabetes Res Clin Pract. 2009;85(1):53-60.

Oppermann M, Alessi J, Hirakata VN, Wiegand DM, Reichelt AJ. Preeclampsia in women with pregestational diabetes - a cohort study. Hypertens Pregnancy. 2020;39(1):48-55.

Owens LA, Sedar J, Carmody L, Dunne F. Comparing type 1 and type 2 diabetes in pregnancy- similar conditions or is a separate approach required? BMC Pregnancy Childbirth. 2015;15:69.

Parellada CB, Asbjörnsdóttir B, Ringholm L, Damm P, Mathiesen ER. Fetal growth in relation to gestational weight gain in women with type 2 diabetes: an observational study. Diabet Med. 2014;31(12):1681-9.

Perichart-Perera O, Balas-Nakash M, Parra-Covarrubias A, Rodriguez-Cano A, Ramirez-Torres A, Ortega-González C, et al. A medical nutrition therapy program improves perinatal outcomes in Mexican pregnant women with gestational diabetes and type 2 diabetes mellitus. Diabetes Educ. 2009;35(6):1004-13.

Persson M, Cnattingius S, Wikström AK, Johansson S. Maternal overweight and obesity and risk of pre-eclampsia in women with type 1 diabetes or type 2 diabetes. Diabetologia. 2016;59(10):2099-105.

Racine JL, Adams JH, Antony KM, Hoppe KK, Iruretagoyena JI, Stewart KS, et al. Metformin Exposure and Risk of Hypertensive Disorders of Pregnancy in Patients with Type 2 Diabetes. Am J Perinatol. 2021;38(11):1103-8.

Rais R, Starikov R, Robert W, Has P, He M. Clinicopathological correlation of large-for-gestational age placenta in pregnancies with pregestational diabetes. Pathol Res Pract. 2019;215(3):405-9.

Rasmussen KL, Laugesen CS, Ringholm L, Vestgaard M, Damm P, Mathiesen ER. Progression of diabetic retinopathy during pregnancy in women with type 2 diabetes. Diabetologia. 2010;53(6):1076-83.

Roland JM, Murphy HR, Ball V, Northcote-Wright J, Temple RC. The pregnancies of women with Type 2 diabetes: poor outcomes but opportunities for improvement. Diabet Med. 2005;22(12):1774-7.

Rowan JA, Luen S, Hughes RC, Sadler LC, McCowan LM. Customised birthweight centiles are useful for identifying small-for-gestational-age babies in women with type 2 diabetes. Aust N Z J Obstet Gynaecol. 2009;49(2):180-4.

Rowe CW, Watkins B, Brown K, Delbridge M, Addley J, Woods A, et al. Efficacy and safety of the pregnancy-IVI, an intravenous insulin protocol for pregnancy, following antenatal betamethasone in type 1 and type 2 diabetes. Diabet Med. 2021;38(4):e14489.

Shannon MH, Wintfeld N, Liang M, Jovanovic L. Pregnancy snapshot: a retrospective, observational case-control study to evaluate the potential effects of maternal diabetes treatment during pregnancy on macrosomia. Curr Med Res Opin. 2016;32(7):1183-92.

Soepnel LM, Nicolaou V, Huddle KRL, Klipstein-Grobusch K, Levitt NS, Norris SA. Maternal and neonatal outcomes following a diabetic pregnancy within the context of HIV. Int J Gynaecol Obstet. 2019;147(3):404-12.

Søholm JC, Vestgaard M, Ásbjörnsdóttir B, Do NC, Pedersen BW, Storgaard L, et al. Potentially modifiable risk factors of preterm delivery in women with type 1 and type 2 diabetes. Diabetologia. 2021;64(9):1939-48.

Stafl L, Benham JL, Frehlich L, Donovan LE, Yamamoto JM. Missed antenatal diabetes care appointments and neonatal outcomes for pregnancies with Type 1 and Type 2 diabetes. Diabetic medicine : a journal of the British Diabetic Association. 2023;40(1):e14950.

Sushko K, Menezes HT, Butt M, Nerenberg K, Strachan P, Usman MA, et al. Trends and Self-Management Predictors of Glycemic Control During Pregnancy in Women With Preexisting Type 1 or Type 2 Diabetes: A Cohort Study. Diabetes Spectr. 2023;36(2):182-92.

Wong VW, Suwandarathne H, Russell H. Women with pre-existing diabetes under the care of diabetes specialist prior to pregnancy: are their outcomes better? Aust N Z J Obstet Gynaecol. 2013;53(2):207-10.

Yamamoto JM, Hughes DJF, Evans ML, Karunakaran V, Clark JDA, Morrish NJ, et al. Community-based pre-pregnancy care programme improves pregnancy preparation in women with pregestational diabetes. Diabetologia. 2018;61(7):1528-37.

Yamamoto JM, Donovan LE, Mohammad K, Wood SL. Severe neonatal hypoglycaemia and intrapartum glycaemic control in pregnancies complicated by type 1, type 2 and gestational diabetes. Diabet Med. 2020;37(1):138-46.

Yee LM, Cheng YW, Inturrisi M, Caughey AB. Effect of gestational weight gain on perinatal outcomes in women with type 2 diabetes mellitus using the 2009 Institute of Medicine guidelines. Am J Obstet Gynecol. 2011;205(3):257.e1-6.
